# Sex-related differences of early cardiac functional and proteomic alterations in a rat model of myocardial ischemia

**DOI:** 10.1186/s12967-021-03164-y

**Published:** 2021-12-11

**Authors:** Bálint András Barta, Mihály Ruppert, Klemens Erwin Fröhlich, Miguel Cosenza-Contreras, Attila Oláh, Alex Ali Sayour, Krisztián Kovács, Gellért Balázs Karvaly, Martin Biniossek, Béla Merkely, Oliver Schilling, Tamás Radovits

**Affiliations:** 1 Experimental Research Laboratory, Heart and Vascular Center, Faculty of Medicine, Semmelweis University, Városmajor u. 68, Budapest, 1122 Hungary; 2 Institute of Surgical Pathology, Faculty of Medicine, University of Freiburg Medical Center, Freiburg, Germany; 3 Faculty of Biology, University of Freiburg, Freiburg, Germany; 4 Spemann Graduate School of Biology and Medicine, University of Freiburg, Freiburg, Germany; 5 MeInBio Graduate School, University of Freiburg, Freiburg, Germany; 6 Department of Laboratory Medicine, Faculty of Medicine, Semmelweis University, Budapest, Hungary; 7 Center for Biological Systems Analysis, University of Freiburg, Freiburg, Germany

**Keywords:** Sex differences, Myocardial ischemia, Left ventricular function, Proteomic changes, Estrogens

## Abstract

**Background:**

Reduced cardiovascular risk in premenopausal women has been the focus of research in recent decades. Previous hypothesis-driven experiments have highlighted the role of sex hormones on distinct inflammatory responses, mitochondrial proteins, extracellular remodeling and estrogen-mediated cardioprotective signaling pathways related to post-ischemic recovery, which were associated with better cardiac functional outcomes in females. We aimed to investigate the early, sex-specific functional and proteomic changes following myocardial ischemia in an unbiased approach.

**Methods:**

Ischemia was induced in male (M-Isch) and female (F-Isch) rats with sc. injection of isoproterenol (85 mg/kg) daily for 2 days, while controls (M-Co, F-Co) received sc. saline solution. At 48 h after the first injection pressure–volume analysis was carried out to assess left ventricular function. FFPE tissue slides were scanned and analyzed digitally, while myocardial proteins were quantified by liquid chromatography–tandem mass spectrometry (LC–MS/MS) using isobaric labeling. Concentrations of circulating steroid hormones were measured with LC–MS/MS. Feature selection (PLS and PLS-DA) was used to examine associations among functional, proteomic and hormonal datasets.

**Results:**

Induction of ischemia resulted in 38% vs 17% mortality in M-Isch and F-Isch respectively. The extent of ischemic damage to surviving rats was comparable between the sexes. Systolic dysfunction was more pronounced in males, while females developed a more severe impairment of diastolic function. 2224 proteins were quantified, with 520 showing sex-specific differential regulation. Our analysis identified transcriptional, cytoskeletal, contractile, and mitochondrial proteins, molecular chaperones and the extracellular matrix as sources of disparity between the sexes. Bioinformatics highlighted possible associations of estrogens and their metabolites with early functional and proteomic alterations.

**Conclusions:**

Our study has highlighted sex-specific alterations in systolic and diastolic function shortly after ischemia, and provided a comprehensive look at the underlying proteomic changes and the influence of estrogens and their metabolites. According to our bioinformatic analysis, inflammatory, mitochondrial, chaperone, cytoskeletal, extracellular and matricellular proteins are major sources of intersex disparity, and may be promising targets for early sex-specific pharmacologic interventions.

**Graphical Abstract:**

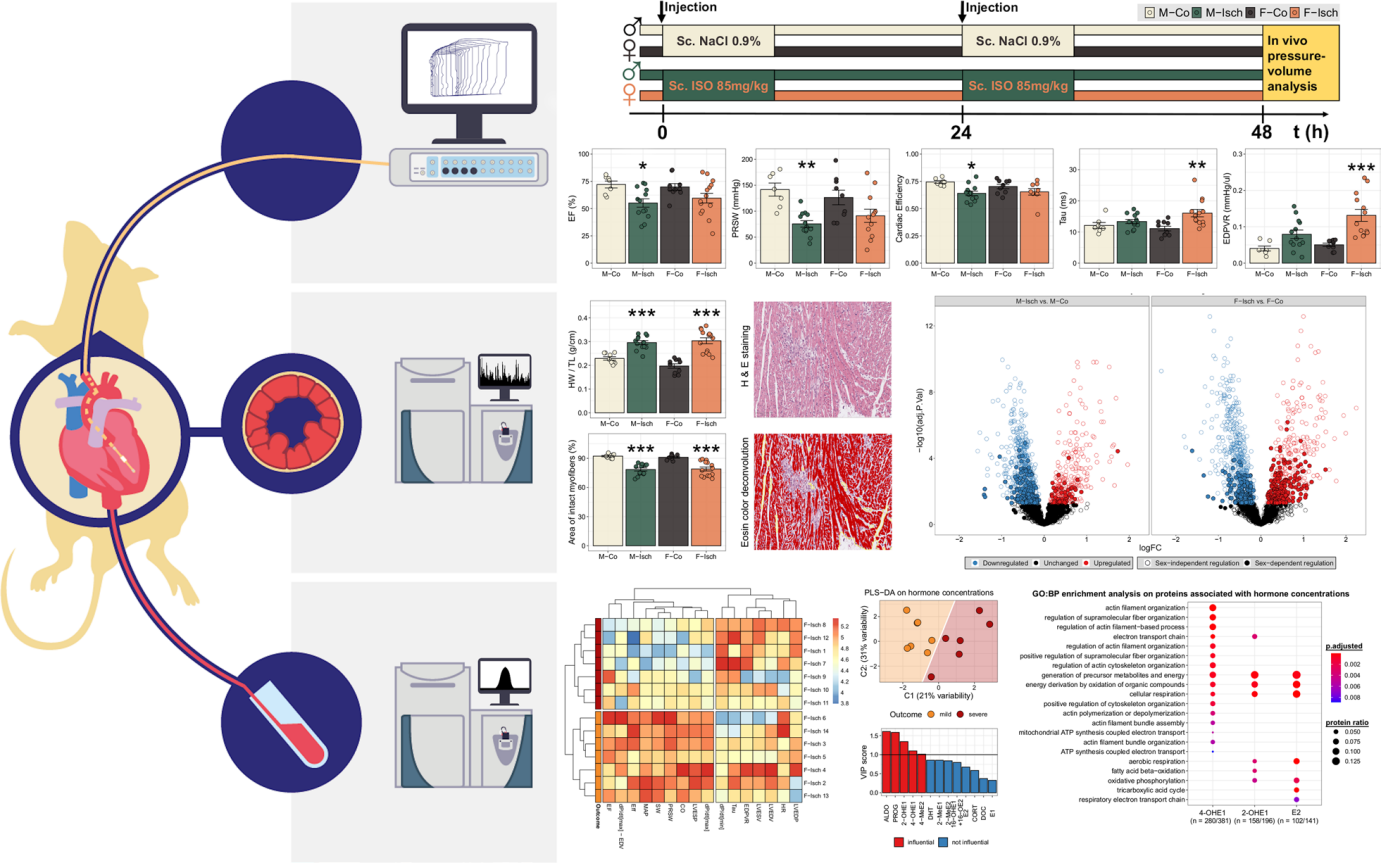

**Supplementary Information:**

The online version contains supplementary material available at 10.1186/s12967-021-03164-y.

## Introduction

Sex has been shown to have a major impact on the pathophysiology, presentation and outcomes of cardiovascular diseases [1]. This phenomenon is particularly apparent in coronary artery diseases (CAD). The occurrence of myocardial infarction (MI) in men is expected earlier and with higher severity compared to age-matched women [1]. The exact mechanisms underlying this discrepancy are still poorly understood, but a scientific consensus has formed around the possible protective role of estrogen hormones [2, 3]. Although estradiol has been investigated with regard to its protective effects under ischemic conditions [4, 5], the effect of estrogen metabolites remains understudied despite their potential to modulate estrogen hormone signaling.

Several studies have been conducted on rodents to gain morphological, functional and molecular insights into sex-related differences in response to myocardial ischemic insults. A majority of these investigations have found higher survival rates as well as faster recovery of contractility and relaxation in females in the first few days after MI (as reviewed by Regitz-Zagrosek and Kararigas [1]). Furthermore, myocardial remodeling following MI has been associated with cavity dilation, eccentric hypertrophy and scar thinning in males, while females exhibited concentric hypertrophy with maintained cavity dimensions and stable scar formation [6].

Most of the data currently available in the literature on sex-dependent cardiac functional outcomes shortly after ischemic periods come from ex vivo experiments performed on Langendorff perfused hearts [7, 8] or echocardiographic measurements [9]. The major drawback of the former approach is that the excised heart might not accurately represent in vivo cardiac function, while the disadvantage of the latter method is that echocardiographic functional parameters are largely dependent on loading conditions (changes in preload or afterload). The gold standard method for assessment of left-ventricular function remains in vivo pressure–volume (P–V) analysis, which offers highly sensitive, load-independent indices of systolic and diastolic function measured under physiologic conditions [10]. Therefore, P–V analysis is uniquely suited to evaluate even subtle sex-specific alterations in cardiac function following ischemia.

Mechanistic insights into the molecular background of observed sex differences have been gained from multiple studies performed on a great variety of animal models and species, as well as on cultured cardiomyocytes. Mitochondrial function, lipid and carbohydrate metabolism, inflammation, regulation of cell death and fibrosis are referred to as crucial sources of sex-dependent distinction [3, 11–13]. In each of these biological processes, proteins play a pivotal role. Thus proteomic characterization of sex-related differences in cardiac adaptation after ischemia is indispensable for a complete understanding of underlying pathomechanisms. Traditional 2D gel-based proteomic methods are still the most abundant source of information due to their availability [14]. However, liquid chromatography coupled mass spectrometry (LC–MS/MS) based proteomics is a rapidly advancing field offering substantially higher proteome coverage than traditional methods, while isobaric labeling molecules (iTRAQ, TMT), allow for drastically increased throughput [15].

Isoproterenol is a non-selective beta-adrenergic agonist that is known to have a dose-dependent effect on the heart. In moderate amounts, it leads to an immediate, but short-term increase in cardiac performance due to its positive chronotropic, dromotropic and inotropic properties, while chronic application induces ventricular hypertrophy and cardiac dysfunction. Grant et al. recently reported a lack of sexual dimorphism in both short-term and long-term changes in cardiac function in response to moderate doses of isoproterenol [16]. In high doses, however, it causes diffuse endomyocardial infarction as a result of overburdening the reserves of the myocardium, upsetting the delicate balance of oxygen delivery and consumption [17], which makes it ideal for investigation of sex-related differences in post-ischemic adaptation.

In this study, we aimed to provide a comprehensive and unbiased characterization of sex-specific early cardiac functional and proteomic alterations in a rat model of isoproterenol-induced myocardial ischemia. Furthermore, we investigated the steroid hormone profiles of ischemic female rats and conducted a detailed bioinformatic analysis to establish possible associations among functional outcomes, proteomic and hormonal profiles.

## Materials and methods

### Animals

Our investigation was performed as approved by the Ethics Committee of Hungary for Animal Experimentation, Semmelweis University, Budapest (PEI/001/2374-4/2015) conforming to European Directive 2010/63/EU and the Guide for the Care and Use of Laboratory Animals used by the US National Institutes of Health (Publication No. 85-23, revised 1996). Our study has been interpreted according to the Animals in Research: Reporting In Vivo Experiments Guidelines. The animals were kept in standard conditions (22 ± 2 °C with 12-h light/dark cycles) and were allowed access to laboratory rat diet and water ad libitum during the whole experiment. All animals received humane care.

### Study protocol

**Fig. 1:**
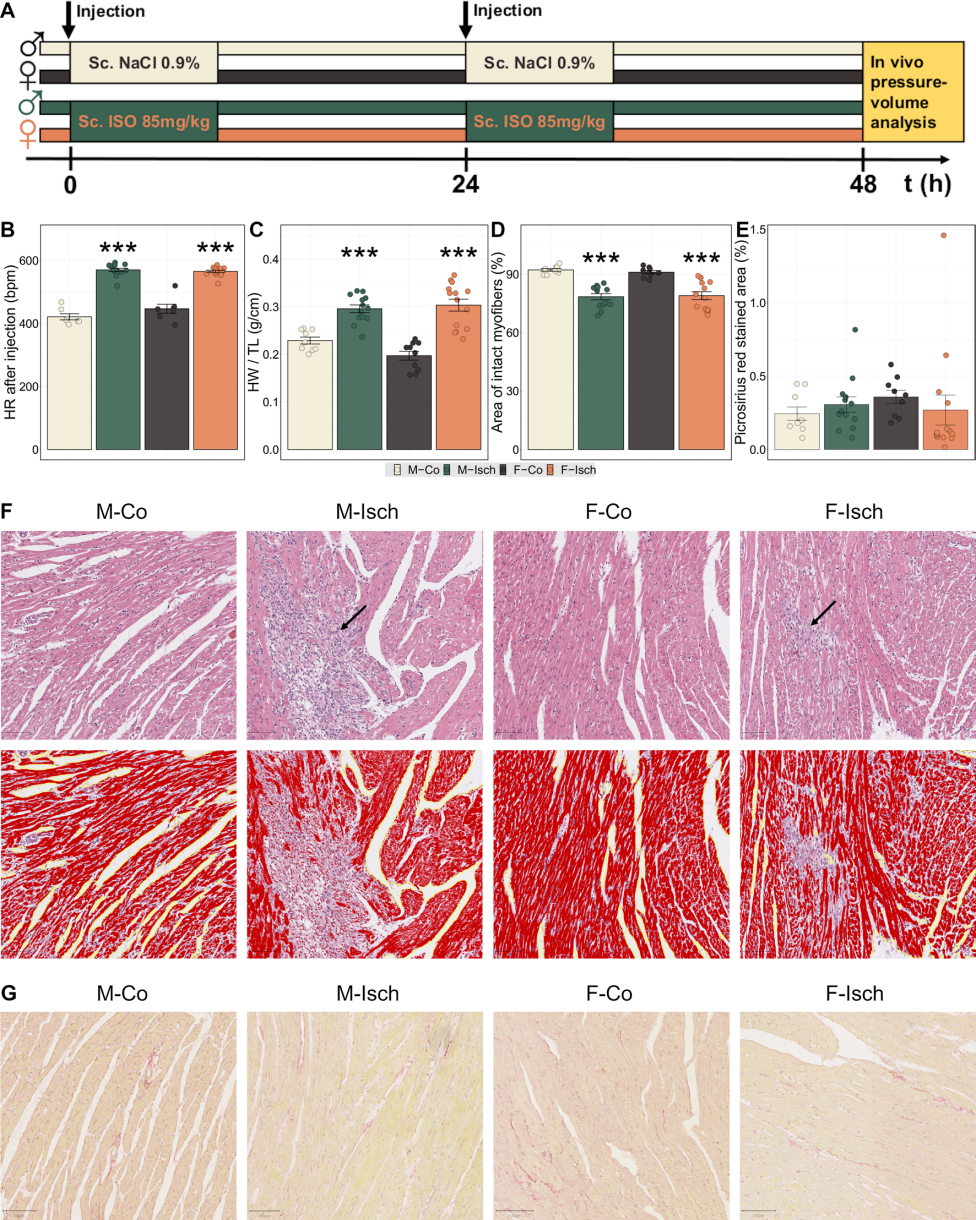
Experimental protocol and assessment of effect of isoproterenol. **A** Study design. **B** Heart rates recorded 2 min after injection of isoproterenol showed a comparable increase due to β-adrenergic activation in both males and females. **C** Increments in heart weight normalized to tibia length indicated a buildup of edema in both sexes. **D** The percentage of the area of intact myofibers on histological slides of the myocardium declined due to isoproterenol therapy in both sexes compared to controls. **E** The percentage of picrosirius red stained area was not significantly changed in response to ischemia at the end of the experimental period. **F** Representative photomicrographs of hematoxylin & eosin stained slides demonstrate diffuse necrotic areas (arrows) embedded within healthy areas of the myocardium after isoproterenol-induced ischemia. Further images (second row) demonstrate how after H&E color deconvolution the eosin color component can be used to quantify the relative area of intact myofibers in the myocardium. First, the overall area of the myocardium is detected. Areas devoid of any myocardial tissue are outlined with yellow and are excluded from the calculation of the overall myocardial area. Then careful thresholding on the eosin color component can identify viable cardiomyocytes with intact myofibrillar structures. These areas (colored red in the software) are then used for the calculation of intact myofibrillar areas which are inversely proportional to and thus are an indirect measure of the extent of myocardial damage, myocardial necrosis. **G**: representative photomicrographs of picrosirius stained slides. Statistical significance of post-hoc test compared to same sex control is highlighted as follows: *P < 0.05, **P < 0.01, ***P < 0.001. M-Co: male control; M-Isch: male ischemic; F-Co: female control; F-Isch: female ischemic; HR: heart rate; HW/TL: heart weight normalized to tibia length

After acclimatization, male and female Wistar rats (180 to 220 g) were assigned to matched control (Co) and ischemic (Isch) groups (Fig. 1A):

- Male control (M-Co, n = 8)
- Male ischemic (M-Isch, n = 20)
- Female control (F-Co, n = 8)
- Female ischemic (F-Isch, n = 17)

All animals underwent body weight measurement (BW) and were briefly anaesthetized using 5% isoflurane. Rats from the ischemic groups were injected with isoproterenol (ISO, 85 mg/kg, subcutaneous, Sigma-Aldrich) daily for 2 consecutive days to induce diffuse subendocardial ischemia as previously described [17], while control rats received equivalent volumes of subcutaneous saline (NaCl 0.9%) injections at the same timepoints. Electrocardiograms were recorded 2 min after injections to assess the acute effect of the drug by a computer-based data acquisition system (PowerLab 16/30 and LabChart Pro software v7; AD Instruments, Colorado Spring, US).

### Hemodynamic measurements

At 48 h after the first injection, invasive left ventricular (LV) pressure–volume (P–V) analysis was performed according to a previously described protocol [18]. In brief, anesthesia was induced with 5% isoflurane and maintained with 1.5% for the duration of the measurements. Animals were tracheotomized, intubated and placed on an automatic heating pad (equipped with a rectal probe) to maintain their body temperature at 37 °C. Fluid administration during the procedure was carried out using a polyethylene catheter inserted into the left external jugular vein. A 2F microtip pressure microcatheter (SPR-838, Millar Instruments, Houston, TX, USA) was advanced into the ascending aorta through the right carotid artery. After 5 min of stabilization, arterial pressure was recorded with a sampling rate of 1000/s. The catheter was then further advanced through the aortic valve to record LV P–V loops. A dedicated P–V analysis program (PVAN, Millar Instruments) was used to calculate the following parameters: systolic arterial blood pressure (SABP), diastolic arterial blood pressure (DABP), heart rate (HR), LV end-systolic pressure (LVESP), LV end-diastolic pressure (LVEDP), LV end-diastolic volume (LVEDV), LV end-systolic volume (LVESV), stroke volume (SV), cardiac output (CO), ejection fraction (EF), stroke work (SW), maximal rate of rise in LV pressure (dP/dt_max_), maximal rate of decrease in LV pressure (dP/dt_min_), time-constant of LV pressure decay Tau (τ, Glantz). Load-independent parameters of systolic and diastolic function were determined based on P–V loops recorded under conditions of gradually decreasing preload achieved by transient occlusion of the inferior vena cava. As indices of contractility preload recruitable stroke work (PRSW) and maximal slope of dP/dt_max_ – EDV relationship (dP/dt_max_-EDV), while as a measure of LV diastolic stiffness the slope of the LV end-diastolic pressure–volume relationship (EDPVR) were determined. Parallel conductance was measured and volume calibrations were performed as previously described [10]. After the invasive hemodynamic measurements, rats were euthanized by exsanguination. The hearts were perfused with oxygenated ringer solution and excised. Heart weight (HW) and tibia length (TL) were measured.

### Histology and immunohistochemistry

Mid-papillary slices of the left ventricle were excised from the explanted hearts and were stored either by snap freezing or by formalin fixation (24 h in 4% buffered paraformaldehyde solution). From the formalin-fixed paraffin-embedded tissue, 5 μm thick slices were sectioned and stained with hematoxylin and eosin (H&E) and picrosirius red. Sections were then digitalized using a whole slide scanner (Ventana DP 200, Roche). The percentage of areas with intact myofibers to the total myocardial area (an indirect measure of the extent of necrosis) was quantified with QuPath 0.2.3 [19] using H&E color deconvolution following the principles set by van Putten et al. [20]. The percentage of picrosirius red stained area was quantified by thresholding in QuPath.

### Proteomics

Cryopreserved myocardial samples were homogenized in 0.1% Rapigest and 100 mM HEPES (pH 7.5) solution. Reductive alkylation was performed using 5 mM tris(2-carboxyethyl)phosphine-hydrochloride (TCEP, Sigma-Aldrich) and 20 mM iodoacetamide (Sigma-Aldrich). Proteins were digested overnight by Trypsin (Worthington, Lakewood, NJ, sequencing grade), and the resulting peptides were desalted on PreOmics columns (PreOmics, Bavaria, Germany). Further steps were performed as described previously[21]. Briefly, peptides were labeled with TMT11plex isobaric label reagents (Thermo Fisher Scientific) and combined into four batches including a cohort-wide normalization channel. Reverse-phase prefractionation (pH = 10) was performed on an XBridge C18 column, 150 mm × 1 mm column containing 3.5 µm particles (Waters) inserted into an Agilent 1100 high performance liquid chromatography system (HPLC). Fractions were analyzed on a Q-Exactive Plus (Thermo Scientific, Bremen, Germany) operating in a data-dependent acquisition (DDA) mode. Mass spectra were analyzed using MaxQuant version 1.6.17.0 [22] with the Uniprot rat database downloaded in November 2020. Quantified peptide intensities were then summarized by MSstatsTMT (R package [23]).

### Western blot

Myocardial LV tissue samples were homogenized in RIPA buffer (Bio-Rad Laboratories, Hercules, CA, USA) containing protease and phosphatase inhibitor cocktail (Roche, Basel, Switzerland), using the Bertin Precellys 24 Tissue Homogenizer with the Bertin Cryolys cooling system (Bertin Technologies). The concentrations of the extracted proteins were measured by BCA assay (Thermo Fisher Scientific). Then, protein homogenates were suspended in sample buffer and heated at 70 °C for 10 min. A total of 20 µg protein for each sample was loaded onto 6–12% acrylamide gels and separated with a sodium dodecyl sulfate polyacrylamide gel electrophoresis system (Bio-Rad Laboratories). Gels were transferred to polyvinylidene fluoride membranes under dry conditions. Membranes were then washed and blocked for 1 h in 5% bovine serum albumin (BSA) in Tris-buffered saline Tween 20 (TBST) at room temperature. Next, membranes were incubated overnight at 4 °C with the following primary antibodies diluted in 2.5% BSA in TBST. The following primary antibodies were used: from Cell Signaling (Cell Signaling Technology, Danvers, MA, USA): VASP (1:1000, ID:#3112), ATP2A2 (1:1000, ID:#4388) and the housekeeping GAPDH (1:5000; ID: #5174); from R&D Systems (R&D Systems Inc., Minneapolis, MN, US): POSTN (1:1000, ID: AF3548); from Abcam (Abcam Inc., Toronto, ON, Canada): OPN (1:1000, ID: ab63856). The blots were washed and incubated with horseradish peroxidase-conjugated secondary antibody (1:5000, 2.5% BSA in TBST) for 2 h at room temperature. The immunoreactive protein bands were developed using Super Signal West Pico Plus (Thermo Fisher Scientific) or SuperSignal West Femto chemiluminescent substrate. The intensity of the immunoblot bands was analyzed with Image J (NIH, Bethesda, MD, USA). The intensity of the bands of the primary targets was normalized to that of the housekeeping GAPDH on the same blot.

### Hormone measurements

The concentrations of circulating endogenous steroid hormones (ALDO aldosterone, PROG progesterone, 2-OHE1 2-hydroxyestrone, 4-OHE1 4-hydroxyestrone, 4-MeE2 4-methoxyestradiol, DHT dihydrotestosterone, 2-MeE1 2-methoxyestrone, 2-MeE2 methoxyestradiol, 16-OHE1 + 16OE2 16-hydroxyestrone + 16-ketoestradiol, E2 estradiol, CORT corticosterone, DOC 11-deoxycorticosterone, E1 estrone) were quantitated using in-house methods developed and validated at the Department of Laboratory Medicine, Semmelweis University, relying on ultra-high performance liquid chromatography-triple quadrupole mass spectrometry with positive electrospray ionization and multiple reaction monitoring (Shimadzu Nexera X2-LCMS-8060, Simkon Kft., Budapest, Hungary). Isotopically labeled internal standards were added to the samples at the beginning of sample preparation to correct for random errors during preparation and analysis. Following deproteinization of 200 µL serum with 600 µL methanol containing the internal standards, ALDO, DHT, DOC, CORT and PROG were extracted by solid phase extraction (Phenomenex Strata-X 60 mg, Gen-Lab Kft, Budapest, Hungary). E1, E2, 2-OHE1, 4-OHE1, 2-MeE1, 2-MeE2, 4-MeE2, 16OHE1 and 16OE2 were first released from their conjugates by incubating another 500-µL fraction of serum with 500 µL β-glucuronidase/aryl sulfatase in pH = 5.0 acetate buffer at 60 °C for 120 min. Subsequently, the mixture was cooled and mixed intensively with 2 × 1 mL ethyl acetate. Following the evaporation of the combined fractions of the organic solvent, 100 µL 1 mg/mL dansyl chloride in acetonitrile and 20 µL 0.5 mol/L sodium carbonate in water were added to the residue. The mixture was incubated at 45 °C for 15 min, followed by the addition of 20 µL 0.5 mol/L hydrochloric acid.

### Statistics and bioinformatics

The data are presented as mean ± SEM. Normal distribution was confirmed by the Shapiro-Wilks method. Two-way analysis of variance (ANOVA) on the factors of “sex” and “ischemia”, followed by a Tukey HSD post hoc test to examine intergroup differences. Contingency tables were analyzed with Fisher’s exact test. Correlations were tested using Pearson’s method. Two-sample T-test was used to compare the area of intact myofibers in the F-isch subgroups. Relationships of functional, proteomic and hormonal profiles were established using log- transformed and quantile normalized values to account for differences among datasets obtained with different methodologies in a subgroup analysis of F-Isch animals. A P-value of < 0.05 was deemed significant for all but gene ontology enrichment analyses (executed with clusterProfiler [24]), where an adjusted P-value of < 0.01 was defined as the threshold.

Differential expression analysis was performed with Linear Models for Microarray Data (Limma R package [25]) by setting up a 2 × 2 factorial design. Significantly changed proteins were grouped into functional groups based on an extensive literature search. Supervised algorithms from the MixOmics R package [26] were utilized to establish associations among functional, proteomic and hormonal datasets. Partial Least Squares Discriminant Analysis (PLS-DA) was employed to examine hormonal-functional relationships where a Variable Importance in the Projection (VIP) score of more than one was considered influential in feature selection. sparse PLS-DA (sPLS-DA) was utilized to provide a visual overview of the extent of inter-sex and ischemic-control differences. Projection to Latent Structure (PLS) analysis was performed to screen for hormone-protein associations, where a VIP score of more than 1.3 was considered influential in feature selection. K-fold cross-validation from MixOmics was used to test the classification efficiency of the models. As a measure of model performance, balanced error rate (BER) was calculated. The number of components to be included in the models was based on the lowest BER and lowest SEM of BER that was achievable.

## Results

### Assessment of effect of isoproterenol treatment

Subcutaneous injection of ISO was followed by an immediate and comparable rise in HR (Fig. 1B) in the Isch groups. The induction of ischemia on two consecutive days has resulted in 38% and 17% mortality in M-Isch and F-Isch rats respectively. The HW/TL ratio was greatly increased, while the area of intact myofibers as quantified on the H&E stained histological slides was markedly decreased in both M-Isch and F-Isch animals (Fig. 1C, D, F). Myocardial collagen content was assessed on picrosirius stained slides and was not found to be different in any of our experimental groups (Fig. 1E, G). These data suggest similar LV myocardial damage in male and female animals that survived the ischemic insult.

### LV functional characterization

**Fig. 2:**
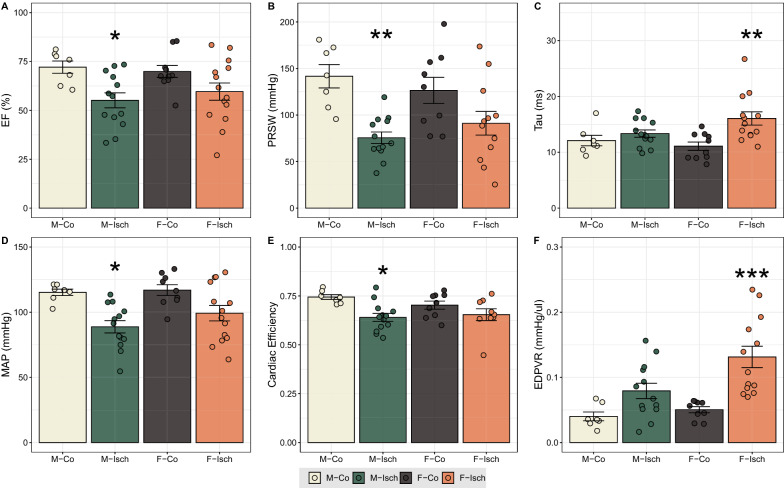
Assessment of systolic and diastolic function. The decline of systolic function was more pronounced in males, while diastolic function deteriorated to a greater degree in females after isoproterenol treatment. **A**, **B** Contractility as assessed by EF and PRSW markedly decreased in males after ischemia. **D** Accordingly, MAP significantly decreased in males as well. **E** The observed deterioration of systolic function of males was underlain by a marked decline in cardiac efficiency. **C** Marked increase in Tau indicated a more pronounced worsening of active relaxation in females. **F** Diastolic stiffness increased only in females as determined by EDPVR. Statistical significance of post hoc test compared to same-sex control is highlighted as follows: *P < 0.05, **P < 0.01, ***P < 0.001. M-Co: male control; M-Isch: male ischemic; F-Co female control; F-Isch: female ischemic; EF: ejection fraction; PRSW: preload recruitable stroke work; MAP: mean arterial pressure; EDPVR: slope of the LV end-diastolic pressure–volume relationship

Although 48 h after the first injection, LVESP was significantly lowered in both Isch groups, MAP, SABP, SADP and SW were found to be markedly diminished only in M-Isch. The decrements of both dP/dt_max_ and dP/dt_max_-EDV were not found to be significant in either sex after ischemia, whereas EF and the gold standard index of contractility (PRSW) have indicated a marked deterioration in systolic function in M-Isch animals. Furthermore, cardiac efficiency was also more severely reduced in males after ischemia (Fig. 2 and Table 1).

**Table 1:** Basic hemodynamic characteristics of experimental groups

Parameter	M-Co	M-Isch	F-Co	F-Isch
BW (g)	205.1 ± 3.3	195.2 ± 5.6	189.8 ± 2.2	193.2 ± 3
SABP (mmHg)	136.2 ± 3.5	103.4 ± 5.0**	129.7 ± 7.4	114.6 ± 7.2
DABP (mmHg)	105.4 ± 1.9	81.52 ± 4.7*	104.22 ± 4.9	91.6 ± 5.3
MAP (mmHg)	115.2 ± 2.2	88.8 ± 4.7*	112.7 ± 5.7	99.2 ± 5.9
HR (bpm)	419.2 ± 11.2	408.9 ± 7.8	416.5 ± 12.1	386.3 ± 9.2
LVESV (µL)	53.5 ± 3.3	114.2 ± 21.0	67.7 ± 11.5	89.7 ± 12.6
LVEDV (µL)	154.6 ± 9.2	207.3 ± 20.7	195.9 ± 14.0	189.4 ± 17.4
LVESP (mmHg)	124.8 ± 3.4	100.1 ± 3.5*	128.8 ± 5.3	103.6 ± 6.0**
LVEDP (mmHg)	11.0 ± 1.1	11.2 ± 0.9	8.6 ± 0.8	13.6 ± 1.7*
SV (µL)	117.5 ± 11.3	111.8 ± 5.6	141.6 ± 5.1	112. ± 11.1
CO (µL/min)	48,694 ± 4106	45,785 ± 2540	58,968 ± 2678	43,175 ± 4240*
SW (mmHg*μL)	14,316 ± 1775	9455 ± 798*	15,111 ± 800	10,626 ± 1234
dP/dt_max_ (mmHg/s)	7458 ± 341	6612 ± 293	8636 ± 420	7531 ± 402
dP/dt_max_-EDV (mmHg/(s*μL))	64.8 ± 3.4	45.7 ± 4.9	59.9 ± 5.9	54.3 ± 4.7
dP/dt_min_ (mmHg/s)	− 9322 ± 542	− 6677 ± 433*	− 11,095 ± 640	− 6736 ± 598***

Statistical significance of post hoc test compared to same-sex control is highlighted as follows: *P < 0.05, **P < 0.01, ***P < 0.001. M-Co male control, M-Isch male ischemic, F-Co female control, F-Isch female ischemic, BW body weight, SABP systolic arterial blood pressure, DABP diastolic arterial blood pressure, HR heart rate 48 h after the first injection, LVESP left ventricular end-systolic pressure, LVEDP left ventricular end-diastolic pressure, LVEDV left ventricular end-diastolic volume, LVESV left ventricular end-systolic volume, SV stroke volume, CO cardiac output, SW stroke work, dP/dt_max_ maximal rate of rise in LV pressure, dP/dt_max_-EDV maximal slope of dP/dt_max_ – EDV relationship, dP/dt_min_ maximal rate of decrease in LV pressure

Rats in the F-Isch group were burdened by markedly decreased CO. In line with this finding, F-Isch rats experienced a higher, but not significant decrease in SV compared to sex-matched control groups. Aspects of diastolic function: active relaxation as determined by Tau and myocardial stiffness as evidenced by LVEDP and EDPVR have deteriorated to a greater extent in F-Isch (Fig. 2 and Table 1).

### Proteomic results

**Fig. 3:**
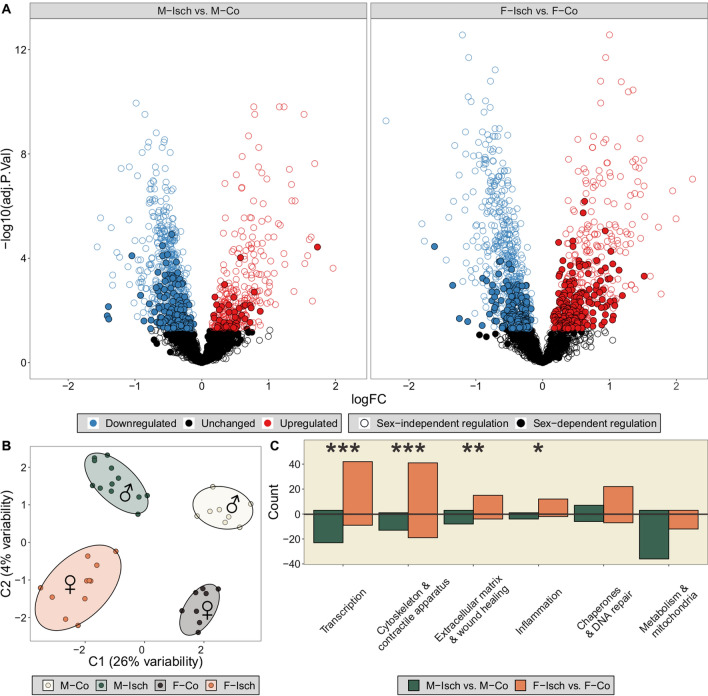
Proteomic comparison of sex-specific changes after ischemia. **A** Results of differential expression analysis. Proteins under sex-specific regulation are highlighted. An adjusted P-value of < 0.05 was considered significant compared to the sex-matched control group. The female proteomic adaptation following ischemia is characterized by a higher number of upregulated proteins compared to males. **B** sPLS-DA analysis on the proteomes of all experimental groups. The supervised method clearly separated all groups, proving that both sex and ischemia affect proteomic profiles. **C** Proteins under sex-dependent regulation in response to ischemia were grouped according to biological categories. Statistically significant association was found between sex and differential expression of proteins with a function in transcription, inflammation, extracellular remodeling and cytoskeletal organization. Statistical significance of Fisher’s exact test is highlighted as follows: *P < 0.05, **P < 0.01, ***P < 0.001. M-Co: male control; M-Isch: male ischemic; F-Co: female control; F-Isch: female ischemic

2224 proteins were identified and quantified from the myocardial samples. sPLS-DA performed on the entire quantified proteome of the myocardium shows that the four experimental groups can be clearly separated from each other with the inclusion of two components (0.0002 ± 0.0069 BER), thus highlighting that there are consistent protein level differences between our groups (Fig. 3B). According to sPLS-DA analysis, the most important proteins in the successful separation of Isch vs. Co were JPH2, ATP5ME, KNG2, CAVIN2, APOOL, MRRF and COQ8A, while the greatest distinction between males and females could be made based on the abundances of A1BG, LIFR, FKBP7, RCN3.

Fisher’s exact test showed a statistically significant association between the number of sex-specific differentially regulated proteins in response to ischemia and sexes (p < 0.001). Although the number of downregulated proteins was similar (603 vs 607, F-Isch vs. M-Isch), females had 56% more sex-specific upregulated proteins compared to males (455 vs. 291, F-Isch vs. M-Isch) (Fig. 3A, Additional file 2: Table S1). The main biological categories that were found to be differentially regulated were comprised of proteins with a role in transcription, cytoskeleton & contraction, extracellular matrix & wound healing and inflammation. In all these categories, females exhibited a higher number of significantly upregulated proteins. Although under the threshold of significance, more metabolic and mitochondrial proteins were downregulated in ischemic males than in females after ischemia (Fig. 3C). To validate the proteomic measurements in the cases of sex-dependent and sex-independent upregulation, as well as unchanged protein expression after ischemia, four proteins with broadly acknowledged cardiovascular relevance were selected: VASP, POSTN, OPN, ATP2A2. Western blotting has confirmed the female-specific upregulation of VASP and OPN in F-Isch, and the sex-independent upregulation of POSTN in M-Isch, as well as in F-Isch compared to respective controls. Furthermore, the expression of ATP2A2 was consistently unaffected by ischemia according to both western blotting and LC–MS/MS measurements (Additional file 1: Fig. S1).

### Association of steroid hormones and cardiac functional outcomes in females after ischemia

**Fig. 4:**
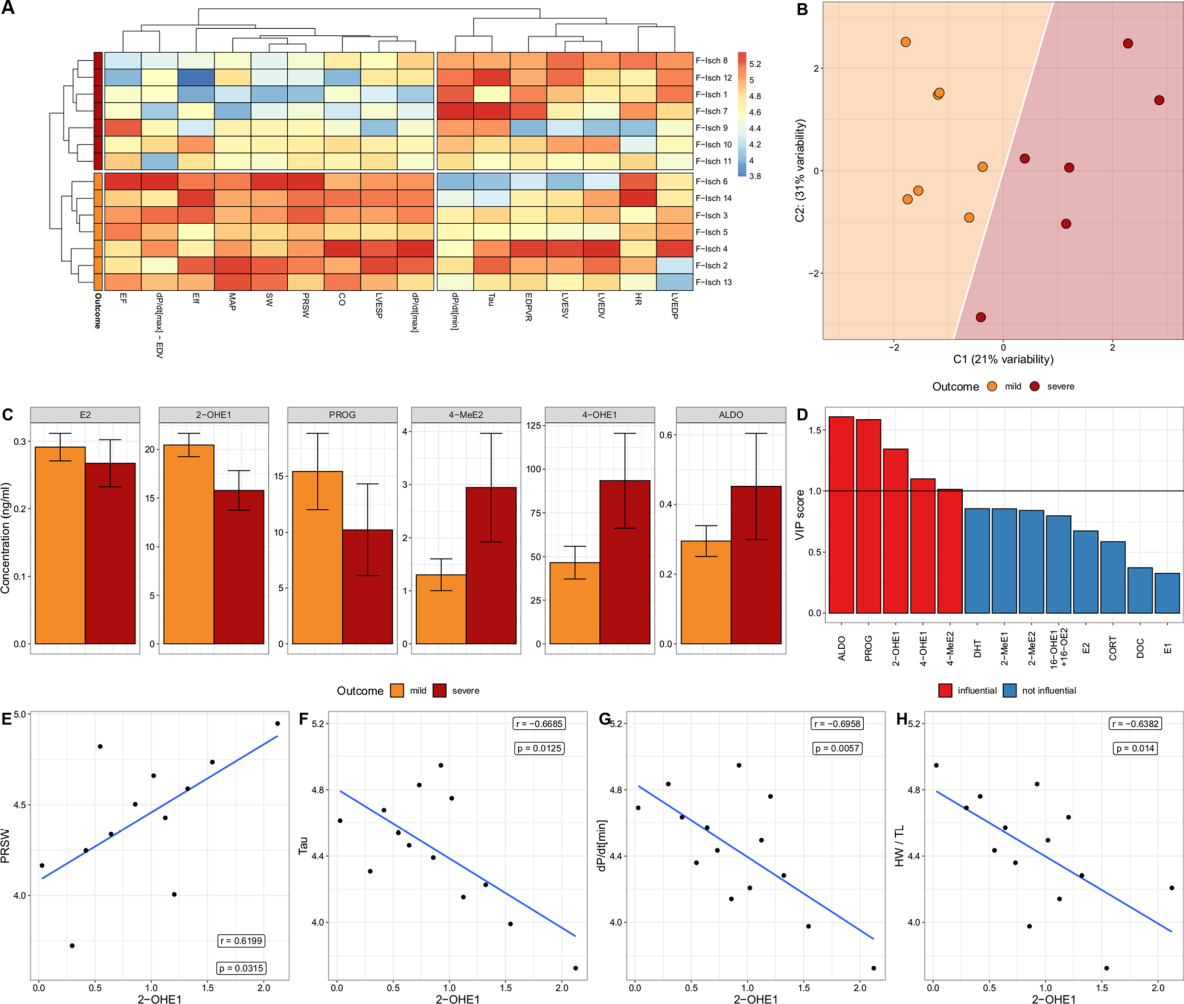
Assessment of influence of circulating steroid hormone levels on functional outcomes after ischemia in females. **A** Hierarchical clustering identified two equally sized subgroups of female ischemic animals characterized by an overall mild or severe functional impairment based on parameters of the pressure–volume analysis. **B** PLS-DA analysis performed on the concentrations of circulating steroid hormone levels in ischemic females resulted in a moderate separation of animals with mild or severe functional outcomes. **C** Absolute concentrations of steroid hormones that were found influential on functional or proteomic profiles. **D** VIP scores extracted from the PLS-DA model identified hormones that contributed the most to the moderate separation of mild and severe functional outcomes. **E**–**H** Out of all influential hormones 2-OHE1 showed strong significant correlations with systolic (PRSW) and diastolic (Tau, dP/dt_min_) as well as HW/TL. For comparison of functional and hormonal datasets acquired by distinct methodologies values were log-transformed and quantile normalized. M-Co: male control; M-Isch: male ischemic; F-Co: female control; F-Isch: female ischemic; SABP: systolic arterial blood pressure; DABP: diastolic arterial blood pressure; HR: heart rate; LVESP: left ventricular end-systolic pressure; LVEDP: left ventricular end-diastolic pressure; LVEDV: left ventricular end-diastolic volume; LVESV: left ventricular end-systolic volume; SV: stroke volume; CO: cardiac output; SW: stroke work; dP/dt_max_ maximal rate of rise in LV pressure; EF: ejection fraction; PRSW: preload recruitable stroke work; dP/dt_max_-EDV: maximal slope of dP/dt_max_ – EDV relationship; Eff: cardiac efficiency; dP/dt_min_: maximal rate of decrease in LV pressure; C1: component 1; C2: component 2; ALDO: aldosterone; PROG: progesterone; 2-OHE1: 2-hydroxyestrone, 4-OHE1: 4-hydroxyestrone; 4-MeE2: 4-methoxyestradiol; DHT: dihydrotestosterone; 2-MeE1: 2-methoxyestrone; 2-MeE2: 2-methoxyestradiol; 16-OHE1 + 16OE2: 16-hydroxyestrone + 16-ketoestradiol; E2: estradiol; CORT: corticosterone; DOC: 11-deoxycorticosterone; E1: estrone; HW/TL: heart weight normalized to tibia length

A relationship between functional outcomes and circulating steroid hormones was investigated in the rats of the F-Isch group in a detailed subgroup analysis. Hierarchical clustering of all P–V hemodynamic parameters has automatically identified two distinct subgroups of 7–7 F-Isch animals which were labeled as mild and severe functional outcomes (Fig. 4A). We investigated if the functional differences observed between these clusters may be related to different percentages of areas of intact myofibers left after infarction (an indirect measurement of infarct size) in the mild and severe F-Isch groups. Nevertheless, no significant difference was found (79.774 ± 2.963% vs 78.259 ± 2.953%, F-Isch mild vs. F-Isch severe). PLS-DA analysis was performed on the steroid hormone profiles of F-Isch rats in an attempt to predict functional outcomes based on hormonal profiles (0.307 ± 0.063 BER, Fig. 4B). Aldosterone, progesterone, 2-hydroxyestrone, 4-hydroxyestrone and 4-methoxyestradiol were found to be influential in the classification of mild vs. severe outcomes based on VIP scores extracted from the model (Fig. 4C, D). Strong correlations were found between 2-hydroxyestrone and crucial P–V parameters of systolic and diastolic function (PRSW, Tau and dP/dt_min_, Fig. 4E–G). The concentration of 2-hydroxyestrone correlated with the HW/TL as well in the F-Isch group (Fig. 4H).

### Association of hormonal and proteomic profiles in females after ischemia

**Fig. 5:**
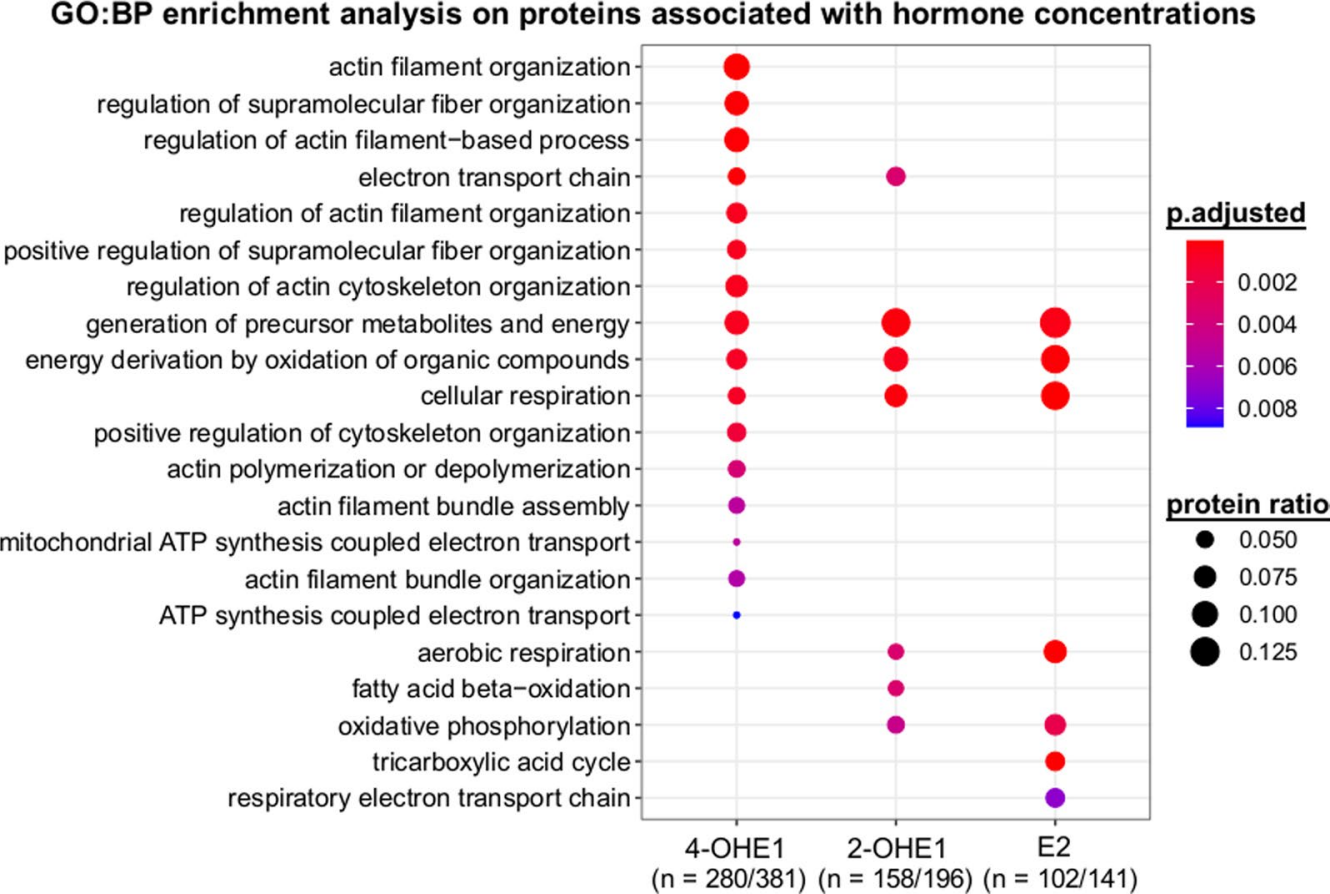
Gene ontology biological process enrichment analysis on proteins associated with hormone concentrations after ischemia in females. 2-OHE1: 2-hydroxyestrone; 4-OHE1: 4-hydroxyestrone; E2: estradiol

PLS was used to establish associations among circulating steroid hormone concentrations and the post-ischemic female myocardial proteome. When VIP scores of proteins per hormone were extracted from the model, a protein with a VIP score of more than 1.3 for a given hormone was considered to show an association with the concentration of the hormone. The top four hormones that were associated with the most proteins are as follows: 4-hydroxyestrone (n = 381), 2-hydroxyestrone (n = 196), estradiol (n = 141) and corticosterone (n = 93). As a second step gene ontology biological process (GO:BP) enrichment analysis was performed on all of the hormone-associated protein groups to find common biological processes, the regulation thereof individual hormones might be responsible for. Only three protein groups have shown significant GO:BP enrichment: 4-hydroxyestrone, 2-hydroxyestrone and estradiol (Fig. 5). Estradiol and 2-hydroxyestrone were associated with proteins responsible for oxidative phosphorylation and aerobic respiration. Furthermore, estradiol was found to associate with proteins from the tricarboxylic acid cycle, while fatty acid beta-oxidation was associated only with 2-hydroxyestrone. Proteins linked to 4-hydroxyestrone were also implicated in cellular respiration, however, elements of supramolecular fibers and the cytoskeleton, in particular proteins related to actin filaments have shown a strong association.

## Discussion

To the best of our knowledge, this is the first study to employ the gold standard methods of pressure–volume analysis, LC–MS/MS based proteomics and steroid hormone measurements to evaluate sex-specific early LV functional alterations following ischemia in a comprehensive manner. Since highly sensitive indices of cardiac performance were measured in vivo, our results reflect LV function independent of loading conditions. We have also provided thorough profiles of sex-dependent proteomic changes by achieving deep proteome coverage on a high number of bioreplicates compared to an earlier cardiac proteomics study performed only on male rats by Lim et al. [14].

### Assessment of sex-independent effects of ischemia

The isoproterenol-induced ischemia model has some unique characteristics compared to surgical alternatives. Instead of permanent or transient ligature of coronary arteries, where the ischemic insult is delimited by the borders of the area that is perfused by the given coronary artery, high dose isoproterenol induces global ischemia that affects the entire myocardium with special emphasis on subendocardial regions. Consequently, this model can be best translated to cases of global ischemia. Subcutaneous administration of isoproterenol led to a sudden and comparable rise in HR in both sexes (Fig. 1). Upon histologic evaluation, small necrotic islands were spread throughout the myocardium, thereby confirming the global nature of ischemia. Using digital whole slide analysis and H&E color deconvolution, the percentage of the area of intact myofibers was calculated, which is an indirect measure of the extent of necrosis. Although previous studies pointed to lower mortality and smaller infarct sizes in females after ischemia (as reviewed by Regitz-Zagrosek and Kararigas [1]) our investigation did not confirm the latter. Our findings are more in line with the works of Shioura et al. who also found that infarct sizes were not different in male and female mice [27], thus sex-specific functional outcomes are not a direct consequence of differences in the extent of myocardial damage. HW/TL was increased in both sexes as well, which was most likely due to myocardial edema caused by the initiation of inflammatory processes, as part of the post-ischemic myocardial remodeling. Correspondingly, among the 7 proteins that have shown the most consistent, sex-independent changes in abundance when separating Isch and Co animals as identified by sPLS-DA analysis, we found KNG2 (T-kininogen 2), an important contributor to inflammatory processes. Of the 7, further 4 proteins (Atypical kinase COQ8A, Ribosome-recycling factor MRRF, MICOS complex subunit APOOL, ATP synthase subunit e ATP5ME) have indicated mitochondrial impairment and another 2 membrane-associated proteins (Junctophilin-2 JPH2, Caveolae-associated protein 2 CAVIN2) have pointed to the disintegration of the T-tubular network of cardiomyocytes [28], that is essential for effective excitation–contraction coupling.

### Systolic function and its proteomic background

Female hearts were found to exhibit improved recovery of contractility in experiments performed on Langendorff perfused hearts after isoproterenol treatment [29, 30]. In line with this finding, 48 h after the first isoproterenol injection, a significant decline in LV contractility (PRSW, EF) was observed only in male ischemic rats, while females displayed overall preserved systolic function. Likewise, a marked decrease in arterial pressure (SABP, DABP, MAP) could only be detected in male animals. Although on the proteomic level we were able to observe a comparable number of downregulated proteins in response to ischemia between the sexes, 56% more proteins were upregulated in females compared to males. After grouping sex-specific up- and downregulated proteins according to biological function based on extensive literature and database searches, we have found significantly more cytoskeletal and contractile proteins upregulated in the myocardium of F-Isch than in M-Isch rats. Together with increased transcriptional activity in females, this could indicate faster replenishment of damaged contractile structures and membrane scaffolding proteins and thus could lead to more effective excitation–contraction coupling and better functional recovery. Although not statistically significant, we have found a greater number of DNA repair enzymes and molecular chaperones upregulated exclusively in females. Of note are ORP-150 [31], HSP90AA1 [32], HSP90AB1 [33], CALR [34], PDIA3 [35] and BIP [36] which are known to exert protective effects under ischemic conditions. As a further source of intersex disparity, we have found a tendency towards a higher number of mitochondrial proteins which were downregulated only in males. This finding could be associated with impaired myocardial energetics that corresponds to the observed decrease in cardiac efficiency of the M-Isch, group, while females displayed maintained Eff values. Of particular interest were proteins with a pivotal role in the citric acid cycle (ACO2, OGDH, IDH3A, IDH2, FH) and fatty acid oxidation (ECI1, ETFRF1, ETFA, ETFB, ETFDH, CISD1, CPT2, ACOT2), all of which were selectively downregulated in males following ischemia but have not changed significantly in females. When we took a look at glycolytic processes, differing strategies of adaptation became apparent. In males, ischemia led to a downregulation of β-enolase, while in females an upregulation of α-enolase was found. α-enolase has been linked to improved cardiac contractility after ischemia [37], which is in line with our current findings.

### Diastolic function and its proteomic background

Conflicting studies have been published regarding diastolic function. In Langendorff setups, the female heart seems to show greater immediate recovery after ischemia [29, 30]. However, in vivo measurements 10 weeks after ischemia indicated increased myocardial stiffness in females [27], possibly due to enhanced extracellular remodeling [6]. In our in vivo study of short-term cardiac functional adaptation, we have found significantly increased end-diastolic pressure in the left ventricle of F-Isch rats. This could be explained by a simultaneous rise in myocardial stiffness, as suggested by EDPVR. Furthermore, Tau has indicated a significant deterioration of active relaxation in females, but not in males, following ischemia. Diastolic impairments can result in a filling deficit and thus might limit the volume of blood that can be ejected during systole. Analogously, females were characterized by a numerical decrease in SV and a significant decrease in CO after ischemia, which was not observed in males. On the proteomic level, we have found a significantly higher quantity of upregulated proteins linked to extracellular matrix remodeling, wound healing and inflammation in females after ischemia. Since extracellular remodeling is a long-term process, inflammation and interstitial fluid accumulation might predominantly determine myocardial stiffness in an acute scenario [38]. Indeed, we have found marked increases in HW/TL after isoproterenol treatment in our experiment, which could be attributed to myocardial edema. Thus, upregulation of inflammatory proteins may contribute to the observed short-term deterioration of diastolic function in females. Nevertheless, this may be beneficial overall, as inhibition of inflammatory processes in the first days after MI was found to result in worse long-term outcomes by impeding cardiac remodeling and tissue repair [39]. Moreover, immediate alterations of extracellular proteins in this critical initial phase of myocardial remodeling should also be addressed. Even though we did not find a substantial increase of collagen accumulation on our picrosirius red stained slides, our proteomic results indicated that extracellular remodeling has already begun. Proteins of interest for the structural integrity of the myocardium are COL18A1, inhibition of which was found to deteriorate myocardial remodeling after infarction [40], and ITGA5, deletion of which was found to inhibit tissue fibrosis in multiple organs [41], both of which were uniquely upregulated in females. Of particular note were matricellular proteins: we have identified SPARC, SPP1 and CTSB to be sex-specifically upregulated in females, while CST3 was upregulated only in males. A transient early rise in SPARC expression has been linked to both fibroblast migration and effective early extracellular remodeling after ischemia [42, 43], while SPP1 was found to be essential for effective collagen synthesis preventing LV dilation. Furthermore, CTSB deficiency was found to slow myocardial remodeling [44]. Additionally, CST3 was identified as a potent inhibitor of CTSB [45], which might contribute to the more passive remodeling strategy of males. This is in line with earlier findings of delayed myocardial remodeling in males compared to females after ischemia [46].

### Associations of sex hormone levels with functional outcomes and proteomic alterations in the female ischemic group.

It is established that estrogen hormone signaling greatly influences the outcome of myocardial ischemia [47]. The most studied estrogen in this respect is E2, but it is far from the only sex hormone that could exert potentially beneficial or detrimental effects. The majority of metabolites of both E1 and E2 are known to bind to estrogen receptor subtypes [48] and may contribute to sex-related differences. To date, gonadectomy and estrogen receptor deletion have been the most commonly used targeted strategies for studying the hormonal influence on post-ischemic recovery [4, 5]. However, both approaches lead to a complete blockade of estrogenic effects, independently of the specific hormones or hormone metabolites. Complete deprivation of estrogen hormones through gonadectomy led to worse outcomes after ischemia, while E2 administration was shown to ameliorate post-ischemic functional impairments and enhance myocardial remodeling in most studies [49]. However, in an investigation by Cavasin et al. E2 supplementation after gonadectomy failed to live up to its protective potential, while testosterone was found to be detrimental to cardiac remodeling and functional recovery [50]. To study the potential influence of sex hormones and their metabolites on post-ischemic recovery, concentrations of a wide range of steroid hormones and metabolites were measured in the F-Isch group. Based on all parameters of left-ventricular function obtained with P–V analysis, automatic hierarchical clustering was performed on rats of the F-Isch group, which identified two distinct clusters of F-Isch rats: one with a mild deterioration of cardiac function and one with a severe impairment of systolic and diastolic performance. To examine the potential role of circulating steroid hormones on functional outcomes, PLS-DA supervised algorithm was utilized for feature selection. Of all the measured hormones, the contributions of PROG, ALDO, 2-OHE1, 4-OHE1 and 4-MeE2 were the most important in the successful classification of mild vs. severe functional outcomes, thus their possible involvement in regulating the pivotal processes of post-ischemic recovery was considered. Out of the five influential hormones, only 2-OHE1 has shown significant and strong correlations with our functional and structural parameters (PRSW, dP/dt_min_, Tau and HW/TL). As a follow-up, protein groupings were established using PLS modeling based on the strength of the associations they showed with the concentration of circulating steroid hormones. GO:BP analysis performed on all hormone-related groups of proteins found significant enrichment only in protein groups associated with 4-OHE1, 2-OHE1 and E2. E2 and 2-OHE1 have shown consistent associations with proteins of oxidative phosphorylation and aerobic respiration. As the main sources of disparity in mitochondrial differential regulation after ischemia, the citric acid cycle and fatty acid oxidation were identified. The former showed a strong association with E2, while the latter seemed to be more related to 2-OHE1. Cytoskeletal and contractile proteins were linked principally to the concentration of 4-OHE1 in particular supramolecular fibers and proteins responsible for the build-up of actin filaments.

## Conclusions

In our rat model of isoproterenol-induced myocardial ischemia, P–V analysis uncovered sex-specific patterns of LV dysfunction: males were characterized by predominantly systolic, while females principally diastolic dysfunction. Distinct proteomic profiles underlay these functional differences. The female post-ischemic myocardial adaptation involved an overall higher number of upregulated proteins, notably in the domains of inflammation, extracellular remodeling, and molecular chaperones. Males exhibited sex-specific downregulation of several proteins involved in the citric acid cycle and fatty acid oxidation. Detailed subgroup analysis of the ischemic female group highlighted the potential role of E2 and estrogen metabolites in determining post-ischemic functional outcomes and proteomic profiles.

## Limitations

The interpretation of the results from the current study is limited to young rats. All data have been acquired from male and female rats that have survived the two-day induction of intermittent myocardial ischemia. The pharmacologic induction of acute global ischemia utilized in our investigation may not be directly translatable to chronic ischemic heart disease in humans. Blood samples for hormonal profiling were taken after P–V measurements to ensure undisturbed hemodynamic characterization. Steroid hormones can be produced and metabolized locally inside the tissue as well, thus circulating levels of sex hormones might not accurately represent hormone levels inside the myocardium. Further studies are needed to assess the effect of supplementation of the steroid hormones highlighted in this study on gonadectomized ischemic rodent models.

## Supplementary Information

**Additional file 1: Fig. S1.** Validation of LC–MS/MS measurements with western blot. A–D: Relative protein expression of VASP, POSTN, OPN and ATP2A2 as measured by LC–MS/MS and western blot. Values were normalized to the mean of the corresponding control group. Statistical significance of post hoc test compared to same-sex control is highlighted as follows: *P < 0.05, **P < 0.01, ***P < 0.001, ****P < 0.0001. M-Co male control, M-Isch male ischemic, F-Co female control, F-Isch female ischemic.

**Additional file 2: Table S1.** Results of differential expression analysis performed with Limma.

## Data Availability

The datasets used and/or analysed during the current study are available from the corresponding author on reasonable request. The proteomic raw data files supporting the conclusions of this article are available in the Mass Spectrometry Interactive Visual Repository (MassIVE, MSV000088184 dataset).
