# Supplementary figures and images for: Sex-related differences of early cardiac functional and proteomic alterations in a rat model of myocardial ischemia
Source: J Transl Med. 2021 Dec 11;19:507. doi: 10.1186/s12967-021-03164-y (PMC8666068; doi:10.1186/s12967-021-03164-y)

# Validation of LC-MS/MS measurements with western blot

## A VASP

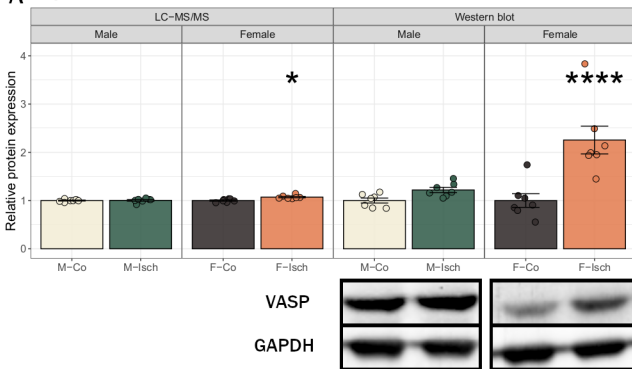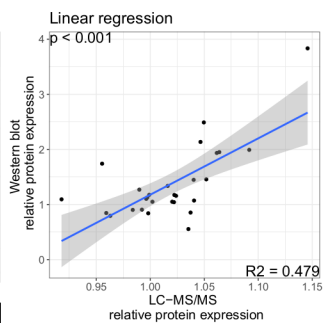

## B POSTN

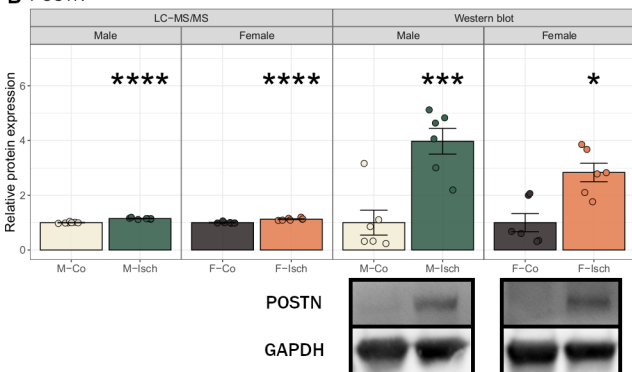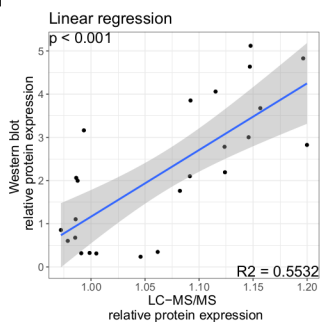

## C OPN

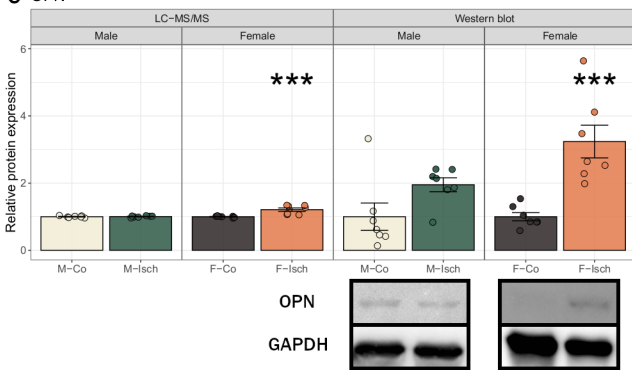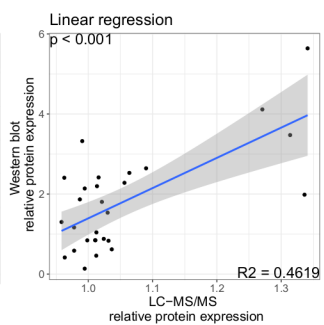

## D ATP2A2

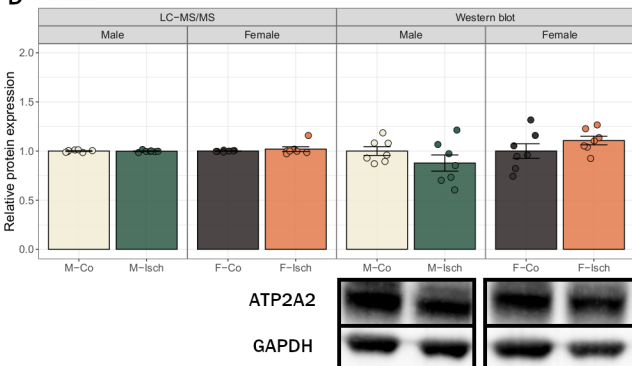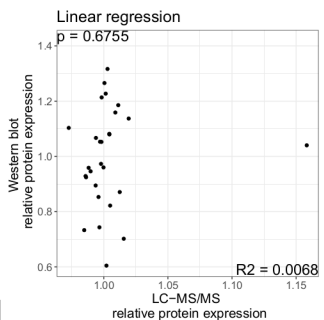

Supplement: Supplementary file 1 — Additional file 1: Fig. S1. Validation of LC–MS/MS measurements with western blot. A–D: Relative protein expression of VASP, POSTN, OPN and ATP2A2 as measured by LC–MS/MS and western blot. Values were normalized to the mean of the corresponding control group. Statistical significance of post hoc test compared to same-sex control is highlighted as follows: *P < 0.05, **P < 0.01, ***P < 0.001, ****P < 0.0001. M-Co male control, M-Isch male ischemic, F-Co female control, F-Isch female ischemic. [file 12967_2021_3164_MOESM1_ESM.pdf]
