# Supplementary material for: Sex-related differences of early cardiac functional and proteomic alterations in a rat model of myocardial ischemia
Source: J Transl Med. 2021 Dec 11;19:507. doi: 10.1186/s12967-021-03164-y (PMC8666068; doi:10.1186/s12967-021-03164-y)
Supplement: Supplementary file 2 — Additional file 2: Table S1. Results of differential expression analysis performed with Limma. [file 12967_2021_3164_MOESM2_ESM.docx]

**Supplements**

Figure S1. Validation of LC-MS/MS measurements with western blot.

A-D: Relative protein expression of VASP, POSTN, OPN and ATP2A2 as measured by LC-MS/MS and western blot. Values were normalized to the mean of the corresponding control group. Statistical significance of post hoc test compared to same-sex control is highlighted as follows: *P<0.05, **P<0.01, ***P<0.001, ****P<0.0001. M-Co male control, M-Isch male ischemic, F-Co female control, F-Isch female ischemic.

Table S1. Results of differential expression analysis performed with Limma.

| UniprotID | logFC_male | adj.P.Val_male | logFC_female | adj.P.Val_female |
| --- | --- | --- | --- | --- |
| Q5BJQ0 | -0.9830 | 1.13E-10 | -1.2007 | 2.76E-13 |
| P13383 | 0.6038 | 1.25E-08 | 0.9998 | 2.74E-13 |
| Q71LX6 | 0.7921 | 3.04E-10 | 0.9421 | 2.02E-12 |
| D4ABA9 | 0.7777 | 1.57E-10 | 0.8670 | 1.63E-11 |
| Q63ZY8 | -0.7948 | 3.56E-09 | -1.1071 | 2.02E-12 |
| P08932 | 1.2302 | 1.57E-10 | 1.3514 | 3.54E-11 |
| Q66H98 | -0.5568 | 2.85E-09 | -0.7108 | 6.00E-12 |
| Q2PS20 | -0.8510 | 3.04E-10 | -0.8546 | 9.28E-11 |
| Q9ERB4 | 0.8521 | 5.65E-09 | 1.1750 | 1.71E-11 |
| A0A0G2K1L0 | 1.1600 | 1.57E-10 | 0.9449 | 2.77E-09 |
| O35094 | -0.5486 | 3.61E-08 | -0.7807 | 1.63E-11 |
| P04692 | -0.8926 | 8.83E-09 | -1.1147 | 6.49E-11 |
| P01048 | 1.5291 | 3.04E-10 | 1.4569 | 2.57E-09 |
| P62501 | 0.8976 | 5.32E-08 | 1.2811 | 4.16E-11 |
| G3V8S6 | -0.6816 | 1.56E-09 | -0.7139 | 1.07E-09 |
| P09456 | -0.5941 | 5.64E-09 | -0.6500 | 1.07E-09 |
| P16409 | -0.6951 | 3.13E-08 | -0.8771 | 2.50E-10 |
| D4AAC3 | -0.7072 | 2.18E-07 | -1.0711 | 9.83E-11 |
| Q66HF9 | 0.5948 | 2.05E-07 | 0.8683 | 1.12E-10 |
| A0A0G2JYM0 | -0.4730 | 8.83E-09 | -0.5235 | 1.91E-09 |
| M0R557 | -0.5478 | 1.91E-07 | -0.7860 | 1.65E-10 |
| Q9JK11 | 0.6989 | 2.04E-09 | 0.6184 | 3.70E-08 |
| B5DEL8 | -0.7630 | 1.36E-07 | -0.9019 | 1.43E-09 |
| D3ZCZ9 | -0.6819 | 5.32E-08 | -0.8019 | 2.82E-09 |
| P29419 | -1.2074 | 3.61E-08 | -1.3155 | 4.76E-09 |
| D3ZHA0 | 0.5997 | 1.91E-07 | 0.7591 | 2.11E-09 |
| Q9QX75 | -1.0847 | 3.13E-08 | -1.1510 | 1.01E-08 |
| Q5M9I5 | -0.7192 | 1.15E-07 | -0.8555 | 4.30E-09 |
| Q62936 | -1.5148 | 2.83E-06 | -2.3445 | 5.40E-10 |
| F1LPG5 | -0.5666 | 3.56E-09 | -0.4556 | 2.23E-07 |
| P08733 | -0.8076 | 5.41E-09 | -0.7227 | 1.35E-07 |
| P02764 | 1.3091 | 3.89E-08 | 1.3901 | 1.60E-08 |
| Q91ZW1 | -0.9623 | 6.89E-08 | -1.0342 | 1.20E-08 |
| D3ZH76 | -0.5741 | 1.64E-06 | -0.8233 | 1.84E-09 |
| D4A8N2 | -0.5286 | 1.06E-06 | -0.7141 | 2.13E-09 |
| Q80XX4 | 0.8475 | 3.15E-08 | 0.8434 | 4.32E-08 |
| Q5U301 | 0.3457 | 1.36E-07 | 0.3866 | 1.60E-08 |
| P16636 | 0.9733 | 9.10E-08 | 1.1207 | 5.33E-09 |
| P11530 | -0.5338 | 5.12E-07 | -0.6536 | 7.23E-09 |
| Q9QUK5 | 0.5165 | 6.31E-08 | 0.5312 | 4.63E-08 |
| D4ADN6 | -0.5051 | 5.38E-06 | -0.7441 | 2.08E-09 |
| P13084 | 0.6335 | 2.83E-06 | 0.9122 | 3.70E-09 |
| P50753 | -0.7585 | 2.18E-07 | -0.8768 | 2.50E-08 |
| P06238 | 1.6893 | 2.35E-08 | 1.4622 | 3.59E-07 |
| P85834 | -0.6610 | 2.65E-05 | -1.1555 | 1.43E-09 |
| Q4KM62 | -0.4053 | 1.34E-05 | -0.6709 | 2.11E-09 |
| Q925Q9 | -0.7051 | 6.32E-07 | -0.8731 | 1.60E-08 |
| P62961 | 0.3224 | 1.09E-05 | 0.5262 | 2.77E-09 |
| B2RZ79 | -0.5441 | 2.24E-06 | -0.7503 | 8.09E-09 |
| Q925G0 | 0.7738 | 6.32E-07 | 0.9595 | 2.22E-08 |
| F1M656 | -0.6411 | 8.46E-06 | -0.9593 | 3.60E-09 |
| Q5PQN7 | -0.5204 | 2.51E-07 | -0.5755 | 4.63E-08 |
| Q5U1W6 | -0.5346 | 1.20E-07 | -0.5266 | 1.28E-07 |
| P23693 | -0.7118 | 2.52E-06 | -0.9551 | 1.13E-08 |
| Q9JI85 | 0.4921 | 1.16E-05 | 0.7476 | 5.62E-09 |
| Q63544 | -0.5747 | 2.52E-06 | -0.7465 | 1.81E-08 |
| Q62813 | -0.7775 | 2.25E-07 | -0.8508 | 2.37E-08 |
| Q5XIC0 | -0.7023 | 5.28E-07 | -0.7628 | 7.31E-08 |
| D3ZY71 | -0.4720 | 1.65E-05 | -0.7403 | 6.44E-09 |
| D3ZU54 | -0.6292 | 4.17E-06 | -0.8473 | 1.74E-08 |
| D3ZYL4 | -0.5909 | 1.13E-05 | -0.8611 | 1.35E-08 |
| P13086 | -0.5697 | 1.42E-06 | -0.6643 | 7.33E-08 |
| G3V6X1 | 1.1461 | 4.66E-06 | 1.4424 | 3.48E-08 |
| Q9Z0G8 | -0.4507 | 7.18E-05 | -0.7824 | 6.85E-09 |
| D3ZZQ4 | -0.5532 | 2.09E-05 | -0.8578 | 1.60E-08 |
| P83565 | -0.6385 | 1.47E-06 | -0.7019 | 1.15E-07 |
| P04785 | 0.7217 | 2.75E-06 | 0.8528 | 8.22E-08 |
| P55207 | 0.9909 | 3.13E-06 | 1.3467 | 2.00E-08 |
| P20788 | -0.4148 | 7.62E-06 | -0.5708 | 4.31E-08 |
| Q9QYU1 | -0.4894 | 3.82E-06 | -0.6151 | 7.10E-08 |
| P50137 | 0.8978 | 3.82E-06 | 1.1629 | 7.33E-08 |
| P07483 | -0.5547 | 2.04E-05 | -0.9454 | 2.48E-08 |
| D4A917 | -0.4300 | 2.18E-07 | -0.3828 | 1.25E-06 |
| Q9Z2J4 | -0.6212 | 1.17E-05 | -0.8818 | 3.48E-08 |
| Q920G2 | -0.4300 | 2.98E-06 | -0.5207 | 1.06E-07 |
| P70567 | -0.4076 | 2.19E-05 | -0.5964 | 2.25E-08 |
| Q6AYF2 | 0.3742 | 3.73E-04 | 0.7440 | 5.62E-09 |
| P62632 | -0.7884 | 7.72E-07 | -0.7676 | 5.95E-07 |
| Q5RKI9 | -0.7419 | 1.74E-06 | -0.8112 | 2.66E-07 |
| P11030 | -0.6169 | 3.06E-06 | -0.7246 | 1.57E-07 |
| P09006 | 0.5380 | 6.40E-05 | 0.9684 | 2.19E-08 |
| F1M124 | -0.3740 | 1.64E-05 | -0.5215 | 4.32E-08 |
| Q9Z269 | -0.6581 | 2.75E-06 | -0.8123 | 5.15E-08 |
| F1M8K0 | -0.4547 | 2.57E-07 | -0.4281 | 2.57E-06 |
| B1WC75 | 0.9912 | 1.55E-05 | 1.5120 | 1.75E-08 |
| D3ZAF5 | 1.3411 | 1.48E-07 | 1.0932 | 6.30E-06 |
| B1PRL5 | -0.6101 | 5.99E-06 | -0.7430 | 1.15E-07 |
| G3V6L9 | -0.6017 | 5.32E-06 | -0.7211 | 1.67E-07 |
| F1M853 | 0.7577 | 1.52E-05 | 1.0133 | 7.33E-08 |
| Q9WVJ6 | 0.5915 | 8.90E-06 | 0.7565 | 1.15E-07 |
| P05942 | 1.6351 | 1.54E-05 | 2.2419 | 9.22E-08 |
| M0R9L0 | -0.4544 | 2.22E-05 | -0.6486 | 7.33E-08 |
| P17764 | -0.7664 | 7.03E-07 | -0.7189 | 1.97E-06 |
| D4A4W6 | -0.4300 | 2.11E-04 | -0.7386 | 1.92E-08 |
| Q4QQV6 | 1.0782 | 1.52E-05 | 1.4221 | 1.15E-07 |
| A0A0G2JYI7 | 0.3514 | 2.83E-06 | 0.3808 | 6.13E-07 |
| P07632 | -0.5741 | 2.09E-05 | -0.7804 | 1.06E-07 |
| M0R7S5 | -0.3964 | 9.42E-05 | -0.6258 | 4.49E-08 |
| A0A0G2JYH7 | -0.6331 | 1.04E-04 | -1.0106 | 4.49E-08 |
| Q62632 | 0.6374 | 8.20E-05 | 1.0030 | 5.26E-08 |
| Q4KLN7 | 0.6166 | 1.52E-05 | 0.7550 | 1.94E-07 |
| A0A0G2K2D6 | -0.6490 | 1.74E-06 | -0.6489 | 1.70E-06 |
| F1M392 | -0.4384 | 5.18E-06 | -0.5014 | 6.16E-07 |
| Q62920 | -0.5241 | 8.38E-06 | -0.6144 | 3.90E-07 |
| Q06647 | -0.7294 | 1.06E-07 | -0.4581 | 1.30E-04 |
| F7EXQ7 | -0.7777 | 5.59E-07 | -0.6264 | 1.17E-05 |
| P41777 | 0.7411 | 2.83E-06 | 0.7251 | 1.63E-06 |
| P19332 | -0.4359 | 2.04E-05 | -0.5695 | 2.46E-07 |
| D3ZD09 | -0.6178 | 1.76E-05 | -0.7880 | 2.82E-07 |
| D3ZGK0 | -0.3888 | 8.90E-06 | -0.5167 | 1.60E-07 |
| O88797 | 0.5584 | 4.23E-05 | 0.7886 | 1.58E-07 |
| M0R7T6 | -0.6642 | 8.64E-06 | -0.7978 | 6.55E-07 |
| P17046 | 0.4540 | 5.51E-05 | 0.6520 | 1.35E-07 |
| B2RYS2 | -0.6122 | 6.56E-07 | -0.5222 | 1.25E-05 |
| A0A0G2K5E4 | -0.5577 | 9.36E-06 | -0.6130 | 7.13E-07 |
| P24483 | 0.9173 | 8.10E-07 | 0.7756 | 1.25E-05 |
| D3ZEY0 | -0.4854 | 1.16E-06 | -0.4193 | 9.87E-06 |
| O35115 | -0.8879 | 2.10E-05 | -1.1502 | 4.46E-07 |
| A0A096MK47 | -0.5520 | 5.16E-05 | -0.7769 | 2.25E-07 |
| M0R785 | -0.3798 | 3.11E-04 | -0.6572 | 7.33E-08 |
| D3ZXF9 | -0.5163 | 7.61E-06 | -0.5584 | 1.42E-06 |
| A0A0G2KAQ5 | -0.5869 | 7.17E-06 | -0.6534 | 1.62E-06 |
| Q4FZY0 | 0.5556 | 1.59E-04 | 0.9008 | 1.33E-07 |
| Q03344 | -0.5573 | 5.35E-05 | -0.7615 | 2.80E-07 |
| Q5I0P2 | -0.5413 | 1.56E-05 | -0.6418 | 7.95E-07 |
| D3ZF21 | -0.6007 | 1.60E-05 | -0.7160 | 7.99E-07 |
| A0A0G2K161 | -0.4923 | 1.88E-05 | -0.5646 | 7.70E-07 |
| P49432 | -0.7376 | 5.80E-07 | -0.5677 | 4.80E-05 |
| E9PT87 | -0.5302 | 4.30E-05 | -0.7107 | 4.28E-07 |
| D3ZIC4 | -0.4185 | 7.97E-05 | -0.6092 | 2.66E-07 |
| F1LU71 | -1.3555 | 6.70E-06 | -1.4044 | 2.70E-06 |
| D3ZIN7 | -0.3795 | 2.21E-04 | -0.6094 | 1.35E-07 |
| P06866 | 1.3953 | 6.32E-07 | 1.0535 | 4.83E-05 |
| D4A6X4 | -0.4898 | 7.93E-05 | -0.6805 | 3.48E-07 |
| Q5EBC0 | 0.4842 | 1.04E-02 | 1.3894 | 3.12E-08 |
| D4A4L5 | -0.5300 | 7.24E-05 | -0.7389 | 4.08E-07 |
| Q68FX4 | 0.7428 | 4.23E-05 | 0.9684 | 6.55E-07 |
| Q9WVA1 | -0.4679 | 4.25E-04 | -0.7829 | 1.28E-07 |
| P05964 | 1.2424 | 1.71E-04 | 1.9445 | 2.60E-07 |
| O35889 | -0.4311 | 2.04E-05 | -0.5015 | 1.45E-06 |
| Q9Z1Z3 | -0.4405 | 3.02E-06 | -0.4021 | 1.19E-05 |
| D3ZHA7 | -1.0001 | 2.10E-05 | -1.1637 | 1.41E-06 |
| P15205 | 0.4770 | 4.58E-05 | 0.6066 | 7.31E-07 |
| Q4PP99 | -0.5763 | 2.53E-03 | -1.2495 | 6.46E-08 |
| Q63416 | 0.8845 | 4.15E-04 | 1.5161 | 1.69E-07 |
| B5DEN5 | 0.3298 | 1.07E-03 | 0.6429 | 1.06E-07 |
| P42930 | 0.7761 | 6.70E-06 | 0.8621 | 6.26E-06 |
| B0BN99 | -0.5581 | 1.56E-05 | -0.6078 | 2.70E-06 |
| Q63945 | 0.2704 | 5.81E-03 | 0.6465 | 5.07E-08 |
| D3ZVK1 | -0.6410 | 3.48E-05 | -0.7829 | 1.54E-06 |
| A0A0G2JW01 | -0.3371 | 1.87E-03 | -0.6762 | 1.00E-07 |
| Q76MV3 | -0.5616 | 9.21E-06 | -0.5702 | 7.15E-06 |
| P62076 | -0.5627 | 2.48E-05 | -0.6749 | 2.57E-06 |
| P43278 | -0.9532 | 1.16E-04 | -1.3339 | 6.55E-07 |
| P01026 | 0.9230 | 7.96E-05 | 1.2745 | 1.01E-06 |
| Q66HG9 | -0.4786 | 1.59E-04 | -0.6852 | 6.02E-07 |
| O88767 | -0.5331 | 1.64E-06 | -0.4296 | 7.25E-05 |
| B2RYM3 | 0.8539 | 4.15E-04 | 1.3781 | 3.28E-07 |
| P13635 | 0.8173 | 3.80E-04 | 1.3153 | 3.85E-07 |
| B4F7A1 | -0.5866 | 1.82E-04 | -0.8595 | 6.55E-07 |
| B5DER5 | -0.7943 | 5.93E-06 | -0.6990 | 2.07E-05 |
| F1M6Q1 | -1.0892 | 3.37E-06 | -0.9848 | 1.41E-05 |
| Q9Z327 | -0.4638 | 3.48E-05 | -0.5493 | 3.37E-06 |
| F1LRA5 | 1.6074 | 1.92E-05 | 1.7320 | 6.41E-06 |
| Q00566 | -0.5496 | 2.35E-05 | -0.5866 | 5.11E-06 |
| D4ABX6 | -0.4650 | 2.91E-05 | -0.5317 | 4.24E-06 |
| D4A164 | -0.4300 | 1.56E-05 | -0.4580 | 8.33E-06 |
| Q4G063 | 0.7406 | 2.00E-04 | 1.0667 | 8.13E-07 |
| P31044 | -0.5633 | 7.97E-05 | -0.7448 | 1.70E-06 |
| P21571 | -0.6652 | 2.48E-05 | -0.7211 | 5.72E-06 |
| P29117 | -0.6608 | 6.70E-06 | -0.6169 | 2.59E-05 |
| A9UMV9 | -0.6468 | 2.09E-04 | -0.9091 | 1.08E-06 |
| Q63560 | 0.3248 | 1.77E-04 | 0.4565 | 1.38E-06 |
| D4A0Y6 | 0.6599 | 5.02E-05 | 0.7702 | 4.03E-06 |
| Q9QW07 | -0.6810 | 1.54E-05 | -0.6738 | 1.35E-05 |
| Q9QY17 | -0.4401 | 6.70E-06 | -0.3769 | 3.63E-05 |
| D3ZY68 | -0.4535 | 1.76E-04 | -0.6225 | 1.45E-06 |
| Q5U2R8 | 1.5832 | 7.93E-05 | 1.9953 | 3.06E-06 |
| A0A0G2K598 | -0.3731 | 3.98E-04 | -0.5800 | 8.52E-07 |
| P62959 | -0.4361 | 3.03E-04 | -0.6529 | 1.14E-06 |
| A0A0G2JZC3 | -0.4725 | 2.02E-05 | -0.4905 | 1.26E-05 |
| A0A0G2KBA5 | -0.5890 | 2.01E-04 | -0.8240 | 1.63E-06 |
| Q63041 | 0.9055 | 2.65E-05 | 1.0039 | 1.11E-05 |
| D3ZS58 | -0.6190 | 1.11E-05 | -0.5685 | 3.00E-05 |
| D3ZT98 | -0.5028 | 5.78E-04 | -0.8105 | 9.18E-07 |
| P10888 | -0.7281 | 2.46E-06 | -0.5230 | 2.91E-04 |
| P02401 | 0.3307 | 2.88E-04 | 0.4734 | 1.61E-06 |
| P97615 | -0.5969 | 2.28E-04 | -0.8372 | 1.90E-06 |
| P04937 | 0.7024 | 3.13E-05 | 0.7786 | 1.16E-05 |
| Q5XIF3 | -0.4441 | 4.58E-05 | -0.5229 | 8.14E-06 |
| F1LM47 | -0.4914 | 3.01E-05 | -0.5224 | 1.19E-05 |
| P31399 | -0.6926 | 8.46E-06 | -0.6686 | 5.47E-05 |
| P01161 | 1.3474 | 6.25E-07 | 0.6822 | 4.74E-03 |
| D3ZF13 | -0.4567 | 7.71E-03 | -1.0408 | 2.82E-07 |
| P08082 | -0.3327 | 1.16E-04 | -0.4156 | 5.72E-06 |
| Q60587 | -0.7855 | 1.81E-05 | -0.7323 | 4.40E-05 |
| A0A0G2JUM8 | -0.3674 | 2.52E-04 | -0.5031 | 3.75E-06 |
| Q8VBU2 | -0.5646 | 9.33E-05 | -0.7088 | 9.13E-06 |
| A0A0G2JY22 | -0.4509 | 7.18E-05 | -0.5001 | 1.14E-05 |
| Q66HD3 | 0.2686 | 1.24E-02 | 0.6484 | 3.29E-07 |
| Q9Z2P4 | -0.2962 | 9.77E-04 | -0.4457 | 1.62E-06 |
| Q63362 | -0.5991 | 6.73E-05 | -0.6604 | 1.60E-05 |
| D3ZA85 | -0.4890 | 1.10E-04 | -0.5723 | 1.11E-05 |
| A0A0G2JZY0 | -0.7464 | 9.44E-05 | -0.9064 | 5.55E-06 |
| D3ZB55 | -0.7568 | 2.40E-04 | -1.0020 | 5.72E-06 |
| P61149 | -0.3461 | 5.06E-04 | -0.5064 | 3.65E-06 |
| A0A0G2JZR1 | -0.5551 | 8.90E-06 | -0.5036 | 8.60E-05 |
| D4A650 | -0.4344 | 2.11E-04 | -0.5680 | 8.14E-06 |
| Q9WV97 | -0.8379 | 4.19E-05 | -0.8524 | 3.67E-05 |
| P22985 | 0.5108 | 3.15E-04 | 0.6926 | 5.93E-06 |
| O88884 | -0.3771 | 7.61E-04 | -0.5431 | 3.17E-06 |
| F1M8E7 | 1.2275 | 6.59E-05 | 1.3144 | 2.74E-05 |
| Q4FZX5 | -0.4609 | 6.80E-05 | -0.4919 | 2.78E-05 |
| P0DMW0 | 1.1739 | 5.38E-06 | 0.7671 | 8.34E-04 |
| Q6KC51 | -0.3670 | 5.21E-04 | -0.5611 | 2.61E-06 |
| Q6P7A9 | -0.5697 | 8.96E-05 | -0.6300 | 2.51E-05 |
| D4A9G1 | -0.9164 | 1.14E-04 | -1.0431 | 2.14E-05 |
| Q63515 | 0.7769 | 7.10E-05 | 0.8161 | 3.46E-05 |
| Q9EPJ3 | -0.5112 | 1.50E-05 | -0.4084 | 9.76E-05 |
| A0A0G2K2T1 | -0.4476 | 1.64E-04 | -0.5368 | 1.71E-05 |
| Q6PCU8 | -0.4008 | 7.17E-04 | -0.5897 | 5.36E-06 |
| A0A0G2KAD4 | -0.3060 | 1.31E-03 | -0.4948 | 4.16E-06 |
| P62138 | 0.6392 | 3.21E-04 | 0.8180 | 1.19E-05 |
| E9PT65 | -0.4460 | 6.22E-05 | -0.4394 | 5.66E-05 |
| D3ZZ68 | -0.2077 | 1.23E-03 | -0.3269 | 4.65E-06 |
| P26051 | 0.6367 | 1.22E-03 | 0.9963 | 5.11E-06 |
| A0A0G2K014 | 0.9793 | 3.99E-04 | 1.2648 | 1.17E-05 |
| D3ZU13 | 0.2809 | 7.34E-04 | 0.4098 | 7.68E-06 |
| A0A0G2K7M4 | 0.2574 | 4.41E-02 | 0.7445 | 6.03E-07 |
| Q9WTT6 | 0.7457 | 2.82E-05 | 0.6751 | 1.85E-04 |
| D3ZFH5 | 0.8209 | 4.26E-03 | 1.5271 | 2.53E-06 |
| Q66HR2 | 0.4347 | 1.43E-03 | 0.6692 | 5.18E-06 |
| P62078 | -0.4866 | 3.98E-04 | -0.6351 | 1.42E-05 |
| Q6P747 | -0.7700 | 1.80E-04 | -0.8342 | 2.99E-05 |
| D3ZMJ7 | -0.6545 | 6.61E-05 | -0.6934 | 3.45E-05 |
| Q99MC0 | -0.4085 | 6.69E-04 | -0.5629 | 1.13E-05 |
| D3ZKT8 | -0.4386 | 1.10E-04 | -0.4614 | 5.66E-05 |
| A2VD12 | -0.4516 | 5.61E-05 | -0.4239 | 1.39E-04 |
| Q5BJX1 | -0.4880 | 2.23E-03 | -0.7655 | 5.36E-06 |
| Q9JI04 | -0.4129 | 2.05E-05 | -0.3393 | 5.09E-04 |
| M0RD54 | -0.3364 | 4.19E-03 | -0.6060 | 3.84E-06 |
| P06399 | 0.7787 | 3.14E-03 | 1.3405 | 5.02E-06 |
| Q5PQZ9 | -0.4479 | 2.07E-04 | -0.5388 | 1.93E-05 |
| Q9QZ76 | -0.5795 | 3.03E-03 | -0.9885 | 5.18E-06 |
| D3ZF11 | -0.2071 | 2.39E-04 | -0.2289 | 3.78E-05 |
| A0A0G2KBB9 | -0.3191 | 5.54E-04 | -0.3867 | 1.93E-05 |
| D4A3X1 | -0.2586 | 2.06E-03 | -0.4006 | 7.27E-06 |
| D4A197 | -0.4395 | 1.01E-03 | -0.6351 | 1.36E-05 |
| F1LN42 | -0.3304 | 2.81E-04 | -0.3897 | 4.02E-05 |
| P26772 | -0.3276 | 1.24E-03 | -0.4891 | 1.25E-05 |
| M0R3T0 | -0.8128 | 3.28E-04 | -0.9663 | 3.76E-05 |
| F1LR10 | 0.3681 | 2.37E-03 | 0.5882 | 8.14E-06 |
| F1M9B2 | -0.4039 | 1.56E-05 | -0.2852 | 1.39E-03 |
| O08769 | -0.2269 | 5.12E-03 | -0.4127 | 5.04E-06 |
| P21818 | 0.1407 | 2.08E-01 | 0.6274 | 6.55E-07 |
| B4F7E8 | 0.5951 | 5.32E-03 | 1.0815 | 5.18E-06 |
| P70583 | -0.3672 | 9.13E-04 | -0.5067 | 1.96E-05 |
| P62074 | -0.4809 | 1.84E-04 | -0.5176 | 7.88E-05 |
| A0A0G2KBA1 | -0.7890 | 1.75E-04 | -0.8202 | 8.37E-05 |
| M0RDC2 | -0.3913 | 4.80E-04 | -0.4958 | 3.34E-05 |
| P35434 | -0.4395 | 1.19E-04 | -0.4488 | 1.30E-04 |
| Q63514 | 0.7432 | 4.34E-04 | 0.9619 | 3.82E-05 |
| Q3KR86 | -0.3143 | 9.35E-06 | -0.1878 | 4.64E-03 |
| Q9WUH4 | 0.8833 | 5.35E-05 | 0.7316 | 4.22E-04 |
| F1LTG2 | -0.3005 | 2.44E-04 | -0.3217 | 7.42E-05 |
| D4ADD7 | -0.6086 | 1.33E-03 | -0.8528 | 1.92E-05 |
| Q9JKC9 | -0.2698 | 1.07E-03 | -0.3719 | 2.36E-05 |
| Q6P6R2 | -0.4767 | 3.39E-04 | -0.5390 | 6.58E-05 |
| D4A5G8 | -0.5171 | 9.49E-05 | -0.4866 | 2.57E-04 |
| D3Z952 | -0.5234 | 2.05E-05 | -0.3485 | 2.05E-03 |
| P48721 | -0.4623 | 2.65E-05 | -0.3674 | 1.28E-03 |
| Q8K4G6 | -0.5211 | 1.75E-04 | -0.5327 | 1.48E-04 |
| D4A997 | 0.5053 | 1.04E-03 | 0.6625 | 2.74E-05 |
| Q704S8 | -0.5149 | 8.83E-04 | -0.6750 | 3.34E-05 |
| P35745 | -0.5360 | 3.01E-04 | -0.5985 | 9.01E-05 |
| P11240 | -0.6430 | 8.73E-04 | -0.8742 | 3.55E-05 |
| P84100 | -0.5505 | 6.51E-04 | -0.6786 | 4.42E-05 |
| P60841 | -0.3612 | 9.25E-04 | -0.4883 | 3.45E-05 |
| Q6IN37 | 0.7160 | 5.18E-03 | 1.2458 | 9.62E-06 |
| P85125 | -0.3850 | 1.05E-05 | -0.2117 | 8.17E-03 |
| Q9WUW3 | 0.2723 | 1.40E-03 | 0.3860 | 2.71E-05 |
| Q71UF4 | 0.3340 | 4.57E-03 | 0.5581 | 1.15E-05 |
| P84109 | -0.4876 | 4.25E-04 | -0.5646 | 8.07E-05 |
| Q2Q0I9 | 0.8287 | 2.80E-05 | 0.5866 | 1.96E-03 |
| B5DF46 | -0.6250 | 5.79E-06 | -0.2783 | 2.03E-02 |
| P21263 | 0.1678 | 1.36E-01 | 0.6054 | 1.81E-06 |
| M0R5H1 | -0.4178 | 4.28E-04 | -0.4637 | 8.83E-05 |
| G3V824 | 0.9461 | 7.11E-04 | 1.2439 | 2.98E-05 |
| Q9R0J8 | 0.6386 | 4.16E-03 | 1.0307 | 1.42E-05 |
| F1LPD0 | -0.4490 | 6.80E-05 | -0.3716 | 7.68E-04 |
| Q8R511 | 0.3500 | 1.44E-03 | 0.5373 | 2.00E-05 |
| P13941 | 0.6455 | 3.32E-03 | 1.0309 | 1.86E-05 |
| P17475 | 0.7541 | 5.85E-03 | 1.3191 | 1.22E-05 |
| D3ZUB0 | 0.4121 | 2.88E-03 | 0.6360 | 2.04E-05 |
| D4A0T0 | -0.3644 | 9.59E-04 | -0.4715 | 4.87E-05 |
| P11951 | -0.5427 | 4.36E-05 | -0.4051 | 1.51E-03 |
| A0A0G2K865 | 0.8790 | 5.11E-03 | 1.4655 | 1.71E-05 |
| A0A1W2Q6L2 | -1.3332 | 4.37E-04 | -1.7701 | 2.20E-05 |
| Q5BK20 | 0.3330 | 1.92E-02 | 0.6658 | 7.32E-06 |
| P12749 | -0.7846 | 1.73E-04 | -0.6904 | 4.14E-04 |
| P31000 | 0.5896 | 4.50E-03 | 0.9676 | 2.21E-05 |
| Q64240 | 0.4033 | 6.98E-03 | 0.6991 | 1.58E-05 |
| B2RYW3 | -0.6264 | 1.32E-03 | -0.8242 | 5.91E-05 |
| P70566 | -0.4170 | 1.21E-04 | -0.4444 | 8.09E-05 |
| Q8R4R9 | -0.3966 | 3.01E-03 | -0.5940 | 3.13E-05 |
| Q5BJN5 | -0.3915 | 5.24E-03 | -0.6787 | 2.36E-05 |
| P04276 | 0.7314 | 1.94E-03 | 1.1566 | 5.23E-05 |
| Q6J2U6 | -0.4791 | 3.26E-04 | -0.4911 | 2.49E-04 |
| Q5XIN6 | -0.3238 | 8.40E-03 | -0.5739 | 1.64E-05 |
| P25304 | -0.3611 | 6.22E-05 | -0.2640 | 1.88E-03 |
| P02680 | 0.5131 | 1.57E-03 | 0.7072 | 5.93E-05 |
| Q5U2U0 | -0.4151 | 2.31E-02 | -0.8927 | 8.88E-06 |
| P63102 | 0.6803 | 3.36E-03 | 1.0633 | 3.46E-05 |
| D4A1R0 | -1.5654 | 3.66E-05 | -1.1425 | 3.50E-05 |
| Q75UQ2 | 0.4007 | 1.17E-03 | 0.4781 | 7.77E-05 |
| Q4QR73 | -0.8308 | 1.44E-03 | -1.8069 | 4.76E-06 |
| Q5XIG4 | -0.3719 | 6.66E-04 | -0.4205 | 1.43E-04 |
| Q8VHF5 | -0.5267 | 9.70E-04 | -0.6508 | 1.02E-04 |
| F1M049 | -0.5053 | 6.79E-04 | -0.5733 | 1.43E-04 |
| P84087 | -0.6145 | 1.02E-03 | -0.7977 | 5.23E-05 |
| B0BN94 | -0.3481 | 2.11E-04 | -0.3283 | 4.92E-04 |
| Q5M7T5 | 0.6150 | 8.63E-03 | 1.1166 | 2.04E-05 |
| Q6TUF2 | -0.4477 | 9.22E-04 | -0.5344 | 1.23E-04 |
| Q68FR7 | 0.4040 | 8.89E-04 | 0.4864 | 7.09E-05 |
| P85973 | 0.7978 | 1.27E-04 | 0.6701 | 1.09E-03 |
| F1M8T4 | -0.2308 | 3.75E-04 | -0.2282 | 3.32E-04 |
| A0A0G2K860 | -0.4143 | 6.73E-05 | -0.3240 | 1.28E-03 |
| Q06486 | 0.2757 | 3.30E-02 | 0.6469 | 1.03E-05 |
| P38659 | 0.2971 | 4.65E-03 | 0.4875 | 2.24E-05 |
| D3ZME7 | -0.3395 | 7.64E-04 | -0.3775 | 1.67E-04 |
| Q792I0 | -0.4116 | 1.45E-03 | -0.5156 | 8.99E-05 |
| P02564 | 0.6411 | 1.09E-04 | 0.5331 | 1.38E-03 |
| A0A0G2K9L2 | -0.3813 | 8.63E-03 | -0.6591 | 2.36E-05 |
| Q9QXU9 | -0.5296 | 1.93E-03 | -0.7335 | 4.27E-05 |
| Q5M9G3 | 0.1553 | 2.92E-02 | 0.3469 | 1.15E-05 |
| P0DP31 | -0.2822 | 5.70E-05 | -0.1979 | 3.69E-03 |
| Q9Z2L0 | -0.4353 | 1.10E-05 | -0.2118 | 4.86E-02 |
| Q01177 | 0.5003 | 6.82E-03 | 0.8681 | 3.49E-05 |
| P19103 | -0.3725 | 4.16E-03 | -0.5538 | 4.98E-05 |
| F1LPW0 | 0.4402 | 4.09E-02 | 1.1550 | 9.37E-06 |
| O55012 | 0.4829 | 1.80E-03 | 0.6532 | 5.72E-05 |
| M0RAG4 | -0.4864 | 4.49E-04 | -0.5271 | 2.06E-04 |
| Q6UPE1 | -0.4503 | 1.20E-05 | -0.1676 | 7.75E-02 |
| P37805 | 0.3272 | 5.63E-02 | 0.9355 | 8.84E-06 |
| O35796 | -0.4662 | 3.80E-04 | -0.5144 | 5.22E-04 |
| D4ACC5 | -0.4032 | 1.04E-02 | -0.7056 | 2.88E-05 |
| Q5XIG5 | -0.4140 | 2.97E-03 | -0.7132 | 2.13E-05 |
| Q64428 | -0.4590 | 2.21E-04 | -0.4067 | 9.79E-04 |
| P55009 | 0.6465 | 2.53E-03 | 0.8797 | 8.89E-05 |
| P00507 | 0.7600 | 2.21E-04 | 0.6695 | 1.12E-03 |
| P24054 | -0.2750 | 1.28E-02 | -0.4923 | 3.00E-05 |
| P70501 | -0.3361 | 3.03E-03 | -0.4629 | 8.88E-05 |
| Q62812 | 0.4251 | 6.88E-03 | 0.6959 | 4.95E-05 |
| P62630 | 0.4627 | 1.07E-02 | 0.8284 | 3.67E-05 |
| M0RCX0 | -0.4132 | 4.16E-03 | -0.6055 | 7.39E-05 |
| P08461 | -0.4772 | 1.45E-03 | -0.5744 | 1.85E-04 |
| Q9Z214 | -0.4413 | 4.36E-04 | -0.4372 | 6.37E-04 |
| P97531 | -0.2853 | 3.69E-03 | -0.4104 | 5.05E-05 |
| P09812 | -0.5098 | 2.40E-04 | -0.4995 | 6.57E-04 |
| D4A565 | -0.5734 | 4.23E-05 | -0.3374 | 1.37E-02 |
| Q9R080 | -0.4227 | 1.07E-02 | -0.7206 | 3.84E-05 |
| P00564 | -0.5205 | 3.87E-05 | -0.2908 | 1.66E-02 |
| P19132 | -0.2952 | 2.84E-02 | -0.6244 | 2.14E-05 |
| Q9ET61 | -0.7479 | 1.01E-04 | -0.5332 | 2.25E-03 |
| A0A0G2K7I4 | -0.3275 | 8.14E-04 | -0.3475 | 3.82E-04 |
| P14562 | 0.5151 | 6.39E-04 | 0.5231 | 5.02E-04 |
| P61314 | -0.4505 | 2.98E-04 | -0.3709 | 1.24E-03 |
| Q925F0 | -0.3586 | 6.55E-03 | -0.5644 | 6.40E-05 |
| P31977 | -0.2685 | 1.50E-03 | -0.3164 | 2.27E-04 |
| B2GV14 | 0.3936 | 9.32E-03 | 0.6648 | 5.10E-05 |
| A0JPQ4 | -0.4811 | 2.40E-04 | -0.3948 | 1.61E-03 |
| D4A8X8 | 0.4425 | 2.06E-03 | 0.6667 | 4.92E-05 |
| D4A8G7 | -0.2226 | 2.52E-02 | -0.4583 | 2.74E-05 |
| P29418 | -0.7578 | 1.71E-04 | -0.5880 | 2.69E-03 |
| G3V7P1 | -0.3373 | 6.35E-04 | -0.3360 | 6.23E-04 |
| B5DEH0 | -0.2960 | 7.13E-03 | -0.4476 | 6.41E-05 |
| M0RDY6 | -0.2151 | 4.56E-03 | -0.3018 | 9.87E-05 |
| P67779 | -0.4331 | 2.09E-04 | -0.3334 | 2.32E-03 |
| A0A0G2K948 | -0.1996 | 2.42E-02 | -0.4198 | 2.25E-05 |
| Q6B345 | 0.5797 | 1.82E-02 | 1.1234 | 2.73E-05 |
| Q5RJR8 | 0.4011 | 2.42E-02 | 0.8046 | 3.11E-05 |
| P63249 | -0.3659 | 2.83E-03 | -0.4796 | 1.57E-04 |
| Q63ZV7 | 0.7354 | 4.02E-05 | 0.4032 | 6.10E-03 |
| Q8VID1 | -0.5279 | 2.78E-03 | -0.6835 | 1.65E-04 |
| Q920Q0 | -0.4545 | 7.97E-05 | -0.2829 | 9.13E-03 |
| Q9QWJ9 | -1.2704 | 6.01E-05 | -0.8014 | 2.61E-04 |
| P29411 | -0.4772 | 1.28E-03 | -0.5389 | 3.67E-04 |
| A0A0G2JWY8 | -0.3223 | 2.06E-03 | -0.4200 | 1.38E-04 |
| F1LUD3 | 0.3419 | 4.54E-03 | 0.4850 | 1.24E-04 |
| Q64591 | -1.0141 | 5.12E-03 | -1.4431 | 1.17E-04 |
| F7FHF3 | 0.7011 | 1.43E-03 | 0.8244 | 3.81E-04 |
| P62859 | 0.1858 | 5.92E-02 | 0.4504 | 2.17E-05 |
| Q06C60 | -0.3533 | 3.78E-03 | -0.4745 | 1.60E-04 |
| Q4KLH5 | 0.3599 | 5.43E-03 | 0.5284 | 1.22E-04 |
| A0A0G2K2M9 | 0.1627 | 4.41E-02 | 0.3673 | 2.70E-05 |
| B0BNL2 | -0.4042 | 1.17E-03 | -0.4330 | 5.03E-04 |
| P08503 | -0.4650 | 8.83E-04 | -0.4750 | 7.10E-04 |
| P45592 | 0.3014 | 3.22E-02 | 0.6322 | 3.53E-05 |
| Q5RK27 | -0.6277 | 2.14E-04 | -0.4905 | 2.17E-03 |
| D3ZL85 | -0.4173 | 1.93E-04 | -0.3149 | 2.62E-03 |
| Q3B8N7 | -0.5220 | 6.18E-05 | -0.2678 | 2.54E-02 |
| P19234 | -0.3305 | 4.11E-03 | -0.4499 | 1.94E-04 |
| A0A0G2K995 | -0.3058 | 1.43E-02 | -0.5308 | 7.42E-05 |
| D3ZJD3 | -0.9137 | 2.64E-03 | -1.0748 | 2.92E-04 |
| Q9Z339 | 0.6673 | 4.33E-04 | 0.5934 | 1.91E-03 |
| P10960 | 0.3688 | 5.12E-03 | 0.5402 | 1.76E-04 |
| D4A4K6 | 0.3145 | 1.61E-03 | 0.3793 | 5.02E-04 |
| D3ZQX3 | -0.7243 | 1.92E-03 | -0.8363 | 4.13E-04 |
| D4ACP2 | -0.8918 | 8.21E-04 | -1.0039 | 2.50E-04 |
| A0A0G2JY18 | -0.3028 | 1.42E-03 | -0.3344 | 5.72E-04 |
| Q8R560 | 0.5095 | 4.86E-03 | 0.7839 | 1.27E-04 |
| D3ZLD6 | -0.6168 | 4.47E-03 | -0.8522 | 2.06E-04 |
| A0A0G2K6P4 | -0.5168 | 4.94E-04 | -0.4501 | 1.85E-03 |
| Q99PF5 | 0.0893 | 1.00E-01 | 0.2380 | 2.46E-05 |
| P52944 | -0.2680 | 1.70E-02 | -0.4652 | 8.09E-05 |
| A0A0U1RRP3 | -0.4976 | 9.20E-04 | -0.5124 | 6.75E-04 |
| D4ABA5 | -0.3869 | 7.18E-05 | -0.2129 | 2.13E-02 |
| Q6TXE7 | -0.2658 | 8.18E-03 | -0.4035 | 1.67E-04 |
| Q5XI07 | -0.3306 | 1.23E-03 | -0.3397 | 9.93E-04 |
| Q497A9 | -0.3371 | 2.51E-03 | -0.3988 | 4.90E-04 |
| P21670 | 0.7458 | 5.76E-04 | 0.6549 | 2.24E-03 |
| F1LN59 | 0.5354 | 1.04E-02 | 0.8148 | 1.39E-04 |
| Q9Z1A6 | 0.3116 | 1.14E-02 | 0.4988 | 1.40E-04 |
| Q6IUR5 | -0.3197 | 1.42E-03 | -0.3341 | 8.98E-04 |
| P20059 | 0.9798 | 2.14E-03 | 1.1249 | 6.26E-04 |
| D4A7N1 | -0.4276 | 2.02E-03 | -0.4781 | 6.95E-04 |
| P45953 | 0.4756 | 1.67E-03 | 0.5566 | 8.59E-04 |
| D4A5L9 | -0.4555 | 4.91E-03 | -0.6163 | 3.11E-04 |
| D4AEL0 | -0.6893 | 7.76E-04 | -0.6644 | 1.17E-03 |
| Q6TXG9 | 0.5449 | 5.51E-03 | 0.7375 | 2.77E-04 |
| P23928 | 0.2628 | 2.53E-03 | 0.3635 | 6.23E-04 |
| Q68FX0 | -0.3113 | 5.31E-03 | -0.4181 | 2.89E-04 |
| Q9JMB3 | -0.2912 | 6.53E-03 | -0.4130 | 1.63E-04 |
| G3V7P2 | 0.4747 | 1.70E-02 | 0.8158 | 1.24E-04 |
| D3ZZV0 | 1.0825 | 3.74E-04 | 0.8883 | 3.08E-03 |
| D3ZK76 | 0.6492 | 1.43E-02 | 1.2399 | 5.98E-05 |
| Q5RJT0 | -0.2505 | 5.86E-03 | -0.3109 | 1.86E-04 |
| O70513 | 0.5342 | 1.99E-03 | 0.5836 | 8.51E-04 |
| P81155 | -0.4071 | 7.44E-05 | -0.2166 | 4.93E-02 |
| Q641Z6 | -0.3458 | 1.64E-03 | -0.3666 | 1.12E-03 |
| G3V828 | 0.3745 | 5.29E-03 | 0.4898 | 3.83E-04 |
| Q01205 | -0.3621 | 1.80E-03 | -0.3780 | 1.07E-03 |
| Q63638 | -0.3168 | 1.55E-02 | -0.5306 | 1.65E-04 |
| P52909 | 0.1948 | 4.06E-02 | 0.3936 | 7.70E-05 |
| P61023 | 0.3911 | 3.81E-03 | 0.4931 | 5.60E-04 |
| G3V6U0 | 1.7299 | 3.70E-05 | 0.3846 | 1.30E-01 |
| P34901 | 0.3711 | 6.27E-03 | 0.6889 | 1.40E-04 |
| E9PTV0 | -0.2528 | 4.49E-04 | -0.2194 | 5.03E-03 |
| Q5PQX1 | 0.3807 | 2.33E-03 | 0.4348 | 9.82E-04 |
| Q6AXM7 | -0.1947 | 9.67E-03 | -0.2813 | 2.69E-04 |
| Q63016 | 1.9630 | 2.39E-04 | 1.6095 | 4.85E-04 |
| P10860 | -0.9413 | 8.55E-04 | -0.8938 | 8.59E-04 |
| P97629 | -0.9159 | 2.81E-04 | -0.8383 | 3.90E-04 |
| D4AD58 | -1.0461 | 7.97E-05 | -0.4388 | 6.51E-02 |
| D3ZFQ8 | -0.5178 | 8.83E-04 | -0.4818 | 1.82E-03 |
| D3ZUC9 | -0.3174 | 4.58E-04 | -0.2404 | 6.26E-03 |
| P27605 | 0.6431 | 3.99E-03 | 0.8234 | 6.57E-04 |
| D4A644 | -0.2829 | 5.21E-03 | -0.3552 | 5.02E-04 |
| D3ZI60 | -0.5439 | 4.74E-03 | -0.7065 | 3.71E-04 |
| B1PLB1 | -0.4169 | 9.77E-04 | -0.4430 | 8.03E-04 |
| A0A0G2K5P5 | -0.3872 | 9.20E-04 | -0.3470 | 2.92E-03 |
| Q5XII9 | -0.2875 | 3.59E-02 | -0.5460 | 1.07E-04 |
| Q8VHQ7 | -1.2303 | 9.06E-05 | -0.8794 | 2.88E-03 |
| D3ZJJ6 | -0.5527 | 2.37E-03 | -0.5843 | 1.11E-03 |
| Q66HF1 | -0.5143 | 7.24E-05 | -0.2105 | 1.03E-01 |
| Q5RKG9 | 0.2948 | 1.24E-03 | 0.2691 | 2.21E-03 |
| Q4V8F9 | -0.6417 | 8.36E-05 | -0.2780 | 3.94E-02 |
| P04256 | 0.1149 | 2.72E-01 | 0.4539 | 3.54E-05 |
| A0A140UHY3 | 0.3614 | 2.17E-03 | 0.3773 | 1.32E-03 |
| A0A0G2K0S7 | -0.6877 | 6.79E-04 | -0.6195 | 1.46E-03 |
| F1LYQ4 | -0.3546 | 1.21E-01 | -1.6186 | 3.53E-05 |
| P0C1G7 | -0.9139 | 8.20E-04 | -1.1439 | 1.51E-04 |
| G3V8W9 | -0.3981 | 2.69E-03 | -0.4334 | 1.14E-03 |
| P05371 | 1.0007 | 1.96E-03 | 1.0277 | 1.59E-03 |
| B0K020 | -0.5886 | 3.28E-05 | -0.0765 | 6.20E-01 |
| Q9Z330 | 0.3446 | 8.98E-02 | 1.0194 | 5.42E-05 |
| P02466 | 0.4363 | 1.61E-02 | 0.6939 | 2.60E-04 |
| D4A702 | -0.2868 | 6.50E-03 | -0.3771 | 5.92E-04 |
| P39069 | -0.5167 | 1.98E-03 | -0.5231 | 1.85E-03 |
| D3ZL10 | -0.5859 | 7.93E-05 | -0.2247 | 7.66E-02 |
| Q6AXS5 | 0.2466 | 4.03E-03 | 0.2813 | 1.02E-03 |
| O08816 | -0.3841 | 1.01E-03 | -0.3074 | 4.44E-03 |
| A0A0G2JTX7 | -0.6356 | 2.53E-03 | -0.6831 | 1.61E-03 |
| Q8R3Z7 | -0.2327 | 3.02E-03 | -0.2523 | 1.36E-03 |
| Q5BJZ3 | -0.4572 | 8.08E-04 | -0.3805 | 6.07E-03 |
| D4A7U1 | 0.2711 | 1.28E-02 | 0.4053 | 3.96E-04 |
| Q7TP62 | -0.6663 | 3.56E-03 | -0.7260 | 1.23E-03 |
| F1M6T3 | -0.3392 | 3.29E-03 | -0.3864 | 4.89E-04 |
| D4A9A3 | -1.1050 | 2.10E-04 | -0.6255 | 1.68E-02 |
| D3ZRC4 | -0.5253 | 6.28E-03 | -0.7938 | 3.01E-04 |
| O35346 | -0.9401 | 2.97E-04 | -0.7126 | 1.36E-03 |
| A1A5Q0 | -0.3797 | 7.77E-03 | -0.5099 | 7.71E-04 |
| P62997 | -0.2734 | 1.18E-02 | -0.3946 | 3.96E-04 |
| Q5FWS5 | 1.0306 | 1.27E-04 | 0.4199 | 1.55E-02 |
| A0A0G2JUV5 | 0.7918 | 1.21E-03 | 0.9423 | 3.04E-04 |
| Q00918 | 0.3083 | 2.78E-02 | 0.5936 | 2.21E-04 |
| Q9ERU2 | -0.2969 | 3.19E-02 | -0.5329 | 2.39E-04 |
| D3ZMS0 | -0.4995 | 1.74E-03 | -0.4913 | 2.30E-03 |
| P13596 | 0.3518 | 3.90E-03 | 0.4210 | 1.08E-03 |
| D3ZM60 | -0.2951 | 3.72E-02 | -0.5413 | 2.25E-04 |
| Q63159 | -0.2451 | 5.70E-02 | -0.5055 | 1.65E-04 |
| A0A0G2K2P5 | -0.2682 | 7.98E-04 | -0.2021 | 9.40E-03 |
| P56741 | -0.2380 | 1.70E-03 | -0.2133 | 3.80E-03 |
| B2GUY4 | -0.3786 | 2.96E-02 | -0.6444 | 2.09E-04 |
| O35142 | 0.4929 | 2.81E-03 | 0.5164 | 1.61E-03 |
| P47198 | 0.2200 | 1.06E-01 | 0.5427 | 1.23E-04 |
| Q07936 | 0.3433 | 2.32E-02 | 0.5698 | 3.94E-04 |
| Q5XI78 | -0.3805 | 1.38E-04 | -0.1719 | 1.13E-01 |
| M0RC65 | -0.3397 | 1.56E-02 | -0.5021 | 5.34E-04 |
| P63090 | 0.3545 | 3.44E-02 | 0.6320 | 2.82E-04 |
| F1MA98 | 0.1096 | 1.31E-01 | 0.3024 | 1.23E-04 |
| P04642 | 0.7574 | 1.70E-03 | 0.6920 | 4.58E-03 |
| O35964 | 0.2865 | 5.28E-02 | 0.5501 | 2.08E-04 |
| Q5XIH3 | -0.5359 | 1.50E-04 | -0.2184 | 1.12E-01 |
| F1M6Z4 | -0.4597 | 1.91E-02 | -0.7050 | 5.22E-04 |
| M0R7V3 | -0.5459 | 1.67E-03 | -0.4915 | 4.02E-03 |
| Q7TQ11 | 0.5944 | 2.30E-02 | 0.9719 | 4.92E-04 |
| Q5XID0 | 1.5410 | 1.59E-04 | 0.5152 | 4.11E-02 |
| P12075 | -0.6424 | 8.90E-03 | -0.8240 | 1.10E-03 |
| D3ZIF0 | -1.0812 | 1.01E-03 | -1.0632 | 5.50E-04 |
| P07943 | -0.3793 | 3.94E-04 | -0.1972 | 4.47E-02 |
| B5DFN0 | -0.2405 | 5.29E-02 | -0.4653 | 2.65E-04 |
| P09895 | -0.4766 | 1.04E-02 | -0.5968 | 1.02E-03 |
| P02793 | 0.5783 | 9.42E-05 | 0.0829 | 5.72E-01 |
| Q5FVM2 | 0.7496 | 2.52E-03 | 0.8025 | 1.49E-03 |
| A0A0G2JWG6 | 0.2739 | 6.88E-03 | 0.3324 | 1.57E-03 |
| Q9QX81 | 0.2505 | 1.10E-01 | 0.5974 | 1.72E-04 |
| O35303 | -0.3096 | 6.94E-03 | -0.3872 | 1.24E-03 |
| Q6IMY1 | -0.3357 | 1.58E-02 | -0.4808 | 8.15E-04 |
| Q9ER34 | -0.3600 | 3.57E-04 | -0.1930 | 5.56E-02 |
| D3ZDP2 | -0.3661 | 1.31E-02 | -0.4974 | 9.73E-04 |
| Q9JLU4 | -0.3093 | 6.96E-03 | -0.3864 | 1.23E-03 |
| A0A0G2K051 | -0.1500 | 5.34E-03 | -0.1673 | 2.22E-03 |
| D3Z8P1 | -0.1863 | 1.64E-01 | -0.6496 | 1.33E-04 |
| A0A0G2K2B5 | -0.3946 | 3.82E-02 | -0.9365 | 1.27E-04 |
| E9PSQ0 | -0.4782 | 9.32E-03 | -0.6985 | 6.01E-04 |
| P62260 | 0.7571 | 4.40E-03 | 0.8201 | 2.95E-03 |
| D3ZUX5 | -0.3688 | 7.92E-03 | -0.4452 | 1.64E-03 |
| D3ZBN0 | -0.6567 | 3.45E-04 | -0.3504 | 6.50E-02 |
| D3ZM91 | -0.1596 | 4.94E-02 | -0.3065 | 3.76E-04 |
| B0BN98 | 0.4003 | 8.05E-02 | 0.9367 | 2.32E-04 |
| A1L108 | -0.4486 | 4.03E-03 | -0.4900 | 1.36E-03 |
| Q9EQS0 | 0.4348 | 2.82E-02 | 0.8584 | 3.56E-04 |
| A1A5P2 | 0.2810 | 8.51E-02 | 0.6751 | 1.69E-04 |
| Q5XIL4 | -0.2596 | 2.26E-03 | -0.2340 | 6.23E-03 |
| P08081 | 0.3144 | 4.21E-02 | 0.5674 | 4.27E-04 |
| P20761 | 0.5384 | 7.44E-02 | 1.1423 | 2.85E-04 |
| Q5M7W5 | -0.2021 | 2.36E-03 | -0.1868 | 6.08E-03 |
| D4A7Q3 | -0.3829 | 3.60E-02 | -0.6524 | 4.89E-04 |
| Q5U2R6 | -0.4837 | 2.08E-03 | -0.4369 | 4.98E-03 |
| P14480 | 0.3333 | 5.63E-02 | 0.6818 | 3.86E-04 |
| O89049 | 0.1693 | 3.90E-01 | 0.8058 | 1.22E-04 |
| P0CC10 | -0.7437 | 3.12E-03 | -0.7769 | 1.91E-03 |
| F1M6I7 | 1.0089 | 8.35E-04 | 0.6690 | 1.85E-02 |
| Q6AXV4 | -0.9070 | 1.30E-03 | -1.0087 | 8.63E-04 |
| B0K010 | -0.3340 | 3.76E-03 | -0.3366 | 4.31E-03 |
| Q5U334 | 0.8657 | 1.75E-03 | 1.0473 | 7.02E-04 |
| P07150 | 0.3385 | 2.26E-02 | 0.4935 | 8.38E-04 |
| E9PU11 | 0.2094 | 3.89E-02 | 0.3737 | 5.72E-04 |
| Q9WVC7 | -0.2852 | 5.51E-03 | -0.3054 | 3.07E-03 |
| D3ZA84 | -0.1738 | 3.17E-01 | -0.6291 | 1.27E-04 |
| A0A0G2JYE0 | 0.2470 | 1.79E-02 | 0.3504 | 1.08E-03 |
| F1LWG8 | -0.2751 | 1.17E-02 | -0.3440 | 1.57E-03 |
| D3ZJB1 | -0.2909 | 1.87E-02 | -0.4175 | 1.08E-03 |
| P16975 | 0.2036 | 2.17E-01 | 0.6363 | 1.86E-04 |
| Q9JLT6 | 0.2089 | 8.47E-02 | 0.4445 | 3.60E-04 |
| Q810U0 | 0.1191 | 6.13E-02 | 0.2426 | 4.85E-04 |
| P04916 | 0.1921 | 2.46E-01 | 1.0484 | 1.64E-04 |
| Q32PX7 | 0.1163 | 8.24E-02 | 0.2536 | 4.02E-04 |
| P62870 | -0.2525 | 6.49E-03 | -0.2763 | 3.35E-03 |
| Q66HA8 | 0.1387 | 3.47E-01 | 0.5482 | 1.67E-04 |
| P08721 | 0.4870 | 6.10E-02 | 1.0061 | 5.99E-04 |
| G3V7R5 | -0.4483 | 2.78E-03 | -0.4245 | 4.04E-03 |
| P50115 | 0.8498 | 2.72E-02 | 1.3243 | 1.07E-03 |
| Q66H40 | -0.3997 | 1.89E-02 | -0.5571 | 1.40E-03 |
| Q80W96 | -0.8686 | 4.83E-04 | -0.4782 | 1.36E-02 |
| Q63312 | -0.2384 | 2.80E-03 | -0.2051 | 9.57E-03 |
| D3ZW59 | -0.2330 | 3.40E-02 | -0.3818 | 9.77E-04 |
| P08050 | -0.3184 | 2.95E-02 | -0.4649 | 1.03E-03 |
| Q9EPH8 | 0.3102 | 1.04E-02 | 0.3781 | 2.70E-03 |
| P04466 | -0.9037 | 5.98E-04 | -0.4692 | 4.20E-02 |
| Q9WUC4 | -0.3074 | 1.03E-02 | -0.3620 | 2.78E-03 |
| Q920L2 | -0.4339 | 5.84E-04 | -0.2017 | 9.67E-02 |
| D4AC65 | -0.4468 | 2.17E-03 | -0.3398 | 1.58E-02 |
| P06761 | 0.0814 | 2.67E-01 | 0.2883 | 2.70E-04 |
| M0R7B4 | -0.8386 | 1.43E-03 | -0.6268 | 2.49E-02 |
| Q4QQV3 | -0.8598 | 4.44E-03 | -1.1945 | 7.71E-04 |
| D3ZTN2 | -0.7315 | 1.60E-03 | -0.5134 | 2.40E-02 |
| D3ZN59 | -0.6876 | 9.94E-04 | -0.4065 | 3.76E-02 |
| D4A1P2 | -0.4639 | 3.47E-02 | -0.7423 | 1.12E-03 |
| O70351 | -0.4921 | 1.32E-03 | -0.3889 | 1.37E-02 |
| Q5XII6 | 1.0141 | 1.16E-03 | 0.6635 | 1.98E-02 |
| Q9ES39 | -0.3660 | 1.24E-02 | -0.4466 | 2.78E-03 |
| Q9JLJ3 | -0.5095 | 4.33E-04 | -0.1736 | 1.45E-01 |
| P28075 | 0.3507 | 4.75E-03 | 0.3300 | 7.41E-03 |
| D4A7I8 | 0.8055 | 2.85E-03 | 0.9055 | 1.49E-03 |
| P09495 | 0.0464 | 6.82E-01 | 0.4130 | 1.89E-04 |
| Q5EBB0 | 0.5722 | 1.55E-02 | 0.8066 | 1.33E-03 |
| Q68FU3 | -0.3006 | 9.70E-04 | -0.1654 | 6.14E-02 |
| Q62930 | 0.3280 | 4.75E-02 | 0.6018 | 8.59E-04 |
| D4A8H5 | 0.6659 | 1.60E-02 | 0.9337 | 1.36E-03 |
| P17955 | 0.1632 | 8.05E-02 | 0.3508 | 7.91E-04 |
| D3ZHI6 | -0.3179 | 1.66E-02 | -0.4053 | 2.59E-03 |
| Q9WUD2 | 0.9761 | 5.32E-03 | 1.2378 | 1.16E-03 |
| Q09073 | -0.4483 | 4.50E-04 | -0.1522 | 2.08E-01 |
| Q5U1W8 | 0.2315 | 5.44E-02 | 0.3920 | 9.73E-04 |
| Q02765 | 0.7684 | 2.92E-02 | 1.3273 | 1.04E-03 |
| D3ZIS5 | -0.3117 | 2.24E-02 | -0.4327 | 2.12E-03 |
| Q5PQN5 | -0.3166 | 2.01E-02 | -0.4222 | 2.32E-03 |
| B2RZD7 | -0.4269 | 5.43E-02 | -0.7512 | 1.04E-03 |
| P09650 | -0.6899 | 5.06E-03 | -0.6860 | 9.40E-03 |
| O88339 | -0.2552 | 2.42E-02 | -0.3572 | 2.05E-03 |
| A0A0G2K9U6 | -0.5489 | 7.60E-04 | -0.2267 | 8.70E-02 |
| D3ZN42 | -0.2215 | 2.32E-01 | -0.6584 | 3.96E-04 |
| G3V9M6 | 0.2810 | 8.10E-03 | 0.3022 | 6.07E-03 |
| D3ZTB5 | -0.3376 | 4.30E-02 | -0.5542 | 1.33E-03 |
| P50116 | 0.8756 | 3.66E-02 | 1.3838 | 1.55E-03 |
| D4A8Y1 | -0.1852 | 5.09E-02 | -0.3080 | 1.14E-03 |
| P25977 | -0.3730 | 1.22E-02 | -0.4486 | 3.27E-03 |
| D3ZAQ6 | 0.8366 | 1.85E-03 | 1.1063 | 3.54E-03 |
| Q5RJZ6 | -0.2478 | 3.59E-02 | -0.3763 | 1.64E-03 |
| Q4FZU3 | 0.2014 | 9.44E-02 | 0.4050 | 8.09E-04 |
| P20411 | 1.3782 | 1.87E-03 | 1.0424 | 5.03E-03 |
| F1LRH4 | 0.6720 | 5.32E-03 | 0.8898 | 1.62E-03 |
| D3ZM07 | -0.2546 | 4.54E-02 | -0.4080 | 1.38E-03 |
| P20961 | 0.8165 | 1.98E-03 | 0.7292 | 4.58E-03 |
| B0K013 | -0.2454 | 4.51E-02 | -0.4139 | 1.21E-03 |
| D4A2G9 | 0.1927 | 1.22E-01 | 0.4325 | 7.41E-04 |
| Q6AYU5 | 0.3335 | 1.28E-02 | 0.3977 | 3.83E-03 |
| P52925 | 0.1668 | 5.17E-01 | 0.8738 | 3.04E-04 |
| Q2TA68 | -0.4272 | 5.21E-04 | -0.1306 | 2.98E-01 |
| Q68FS4 | 0.2165 | 2.96E-02 | 0.3119 | 2.34E-03 |
| B0LPN4 | -1.3350 | 1.07E-03 | -0.7829 | 1.81E-02 |
| D3ZEH8 | 1.6549 | 4.34E-03 | 1.7712 | 2.33E-03 |
| P62755 | -0.4464 | 4.33E-03 | -0.3539 | 1.69E-02 |
| O35824 | -0.5522 | 4.36E-04 | -0.1066 | 4.81E-01 |
| Q7TP77 | -0.4943 | 1.94E-03 | -0.3213 | 4.51E-02 |
| M0RA86 | -0.2433 | 5.72E-03 | -0.2176 | 1.28E-02 |
| P13803 | -0.3287 | 6.34E-04 | -0.1065 | 2.66E-01 |
| Q5FVR4 | 0.4309 | 1.62E-01 | 1.5184 | 4.82E-04 |
| M0R6L8 | -0.7442 | 1.40E-03 | -0.4547 | 1.43E-02 |
| P0C5I0 | -0.3282 | 1.71E-01 | -0.8396 | 7.29E-04 |
| Q5XIT9 | -0.5882 | 1.43E-02 | -0.6632 | 5.29E-03 |
| D3ZH41 | 0.3754 | 3.52E-02 | 0.5563 | 2.30E-03 |
| Q5EB94 | -0.3635 | 1.74E-02 | -0.4509 | 4.46E-03 |
| P04764 | 0.2585 | 5.43E-02 | 0.4406 | 1.64E-03 |
| Q5RJQ7 | -0.3239 | 6.60E-02 | -0.5843 | 1.45E-03 |
| M0R8A4 | -0.2970 | 1.17E-02 | -0.3121 | 6.64E-03 |
| Q331S7 | 0.3664 | 8.81E-03 | 0.3717 | 9.27E-03 |
| D3ZAW2 | -0.3257 | 4.46E-02 | -0.5211 | 2.04E-03 |
| Q62967 | 0.4139 | 6.38E-02 | 0.8037 | 1.07E-03 |
| A8IHN8 | 0.3408 | 7.12E-03 | 0.3340 | 1.20E-02 |
| F1LYX9 | -0.3305 | 2.60E-02 | -0.4374 | 3.36E-03 |
| G3V928 | 0.5738 | 4.46E-02 | 0.9030 | 2.11E-03 |
| D4A6K4 | -0.2949 | 2.52E-01 | -0.8981 | 5.81E-04 |
| Q08163 | 0.1919 | 1.52E-01 | 0.4744 | 9.43E-04 |
| P12346 | 0.8635 | 4.19E-03 | 0.6748 | 2.35E-02 |
| F1M1M2 | -0.4233 | 1.43E-02 | -0.4503 | 6.20E-03 |
| P60825 | -0.3539 | 1.03E-03 | -0.1534 | 1.57E-01 |
| G3V8N8 | 0.1907 | 3.94E-02 | 0.2845 | 2.48E-03 |
| P27274 | 0.2426 | 1.60E-02 | 0.3073 | 3.04E-03 |
| P97576 | -0.5744 | 6.77E-03 | -0.5177 | 1.45E-02 |
| D3ZC82 | 0.2001 | 3.75E-02 | 0.2906 | 2.80E-03 |
| Q9Z0V6 | 0.5769 | 2.34E-02 | 0.7466 | 4.39E-03 |
| Q7TP15 | 0.4185 | 1.29E-02 | 0.4797 | 6.37E-03 |
| P48004 | 0.4457 | 1.84E-02 | 0.5369 | 5.63E-03 |
| Q9EPF2 | -0.2817 | 3.67E-03 | -0.1920 | 3.44E-02 |
| Q66HD0 | 0.1587 | 1.59E-01 | 0.4058 | 9.77E-04 |
| Q9JJ54 | -0.2856 | 8.83E-04 | -0.0931 | 2.73E-01 |
| A1A5R1 | -0.5023 | 6.37E-04 | -0.0619 | 6.37E-01 |
| A0A0G2K1A2 | 0.2031 | 3.92E-01 | 0.9774 | 6.26E-04 |
| D4AE06 | 0.3430 | 5.08E-02 | 0.5446 | 2.39E-03 |
| P49911 | -0.1842 | 1.20E-02 | -0.1914 | 9.59E-03 |
| A0A0G2JZD5 | -0.3476 | 4.99E-03 | -0.2817 | 2.49E-02 |
| A0A0G2K960 | -0.6774 | 1.34E-03 | -0.3629 | 3.71E-02 |
| P17220 | 0.3648 | 2.08E-02 | 0.4409 | 5.52E-03 |
| D4A3E8 | -0.7361 | 5.44E-03 | -0.6957 | 4.34E-03 |
| Q3B7U1 | 0.3199 | 2.12E-01 | 0.8646 | 7.29E-04 |
| F1M3G7 | 0.2174 | 1.38E-02 | 0.2320 | 9.13E-03 |
| D3ZGL1 | 1.0276 | 4.67E-03 | 0.9769 | 5.34E-03 |
| Q6PEC1 | -0.1596 | 2.03E-02 | -0.1912 | 6.23E-03 |
| D3ZQN7 | -0.2753 | 1.24E-02 | -0.2757 | 1.02E-02 |
| P54001 | 1.1008 | 5.92E-03 | 1.0878 | 4.19E-03 |
| A0A0G2KB52 | -0.4620 | 7.61E-03 | -0.4401 | 9.41E-03 |
| Q4KM49 | -0.4621 | 1.37E-03 | -0.1898 | 1.54E-01 |
| P50339 | -0.5251 | 3.06E-03 | -0.3569 | 4.23E-02 |
| D3ZE72 | -0.2565 | 2.69E-02 | -0.3228 | 4.88E-03 |
| Q68FR6 | 0.6532 | 5.49E-03 | 0.6928 | 4.73E-03 |
| M0RBL8 | -0.5023 | 2.66E-02 | -0.6360 | 2.94E-03 |
| F1M9A4 | -0.7501 | 1.03E-02 | -0.7090 | 1.08E-02 |
| A0A0G2K4R1 | -0.0915 | 2.04E-01 | -0.2525 | 1.12E-03 |
| D3ZZC1 | 0.3247 | 1.43E-02 | 0.3347 | 9.64E-03 |
| O35760 | 0.6316 | 4.16E-02 | 1.0086 | 2.30E-03 |
| O35763 | 0.1818 | 8.34E-02 | 0.3250 | 2.05E-03 |
| Q9EQZ1 | -0.3621 | 3.18E-02 | -0.4902 | 3.96E-03 |
| Q63530 | -0.1465 | 3.79E-01 | -0.5472 | 7.71E-04 |
| Q5FVP7 | -0.4266 | 1.39E-02 | -0.4808 | 6.15E-03 |
| P15800 | -0.1536 | 4.82E-02 | -0.2334 | 3.34E-03 |
| D4A3I4 | -0.3207 | 3.76E-02 | -0.4504 | 4.22E-03 |
| A0A0G2K9K2 | 0.1040 | 4.64E-01 | 0.4610 | 7.06E-04 |
| B2RZ27 | 0.3337 | 2.91E-02 | 0.4378 | 5.43E-03 |
| P18422 | 0.6181 | 1.07E-02 | 0.5886 | 1.49E-02 |
| Q6AYR1 | 0.4451 | 3.02E-02 | 0.5921 | 4.35E-03 |
| Q62760 | -0.2747 | 1.77E-02 | -0.2998 | 8.98E-03 |
| A0A0G2K490 | -0.1830 | 1.66E-01 | -0.4802 | 1.12E-03 |
| Q569B7 | -0.3649 | 1.76E-02 | -0.4096 | 7.60E-03 |
| P35704 | 0.2412 | 3.12E-02 | 0.3208 | 5.30E-03 |
| P30427 | 0.1844 | 1.27E-01 | 0.3859 | 1.67E-03 |
| Q2EJA0 | -0.2243 | 2.82E-02 | -0.2870 | 5.92E-03 |
| P36201 | -0.5550 | 1.91E-02 | -0.6190 | 8.60E-03 |
| Q6QGW5 | 0.4378 | 1.39E-02 | 0.4379 | 1.20E-02 |
| B0K036 | -0.9169 | 2.62E-03 | -0.5537 | 5.20E-02 |
| B2RYA6 | -0.2576 | 6.70E-03 | -0.2073 | 2.63E-02 |
| P63259 | 0.6770 | 3.44E-02 | 1.0526 | 3.27E-03 |
| Q5HZF2 | 0.3335 | 3.04E-02 | 0.4198 | 5.52E-03 |
| Q5U4F3 | 0.0841 | 4.66E-01 | 0.3933 | 8.38E-04 |
| G3V9J1 | 0.3692 | 7.86E-02 | 0.6541 | 2.62E-03 |
| Q62636 | 0.2982 | 1.19E-01 | 0.6971 | 1.55E-03 |
| Q9JJP9 | 0.3418 | 1.01E-03 | 0.0740 | 4.72E-01 |
| B1H219 | -0.1299 | 2.20E-01 | -0.3527 | 1.19E-03 |
| D4AB01 | -0.3472 | 2.55E-02 | -0.4162 | 7.18E-03 |
| Q498C9 | -0.0734 | 3.14E-01 | -0.2447 | 1.09E-03 |
| Q6AYE2 | -0.3004 | 5.21E-03 | -0.2164 | 4.09E-02 |
| Q9EPH2 | 0.4784 | 3.13E-03 | 0.3002 | 6.42E-02 |
| P61459 | -0.3942 | 9.13E-03 | -0.3541 | 2.11E-02 |
| F1MAQ7 | 0.1005 | 3.14E-01 | 0.3286 | 1.08E-03 |
| Q8R2E7 | -0.3611 | 1.32E-01 | -0.7597 | 1.92E-03 |
| D4A3K5 | -0.6834 | 1.34E-03 | -0.2325 | 2.85E-01 |
| Q71UE8 | -0.3207 | 4.44E-02 | -0.4593 | 4.55E-03 |
| A2RUW1 | -0.3038 | 5.34E-02 | -0.4614 | 3.30E-03 |
| D4ADS9 | -0.4703 | 7.45E-03 | -0.4182 | 1.64E-02 |
| Q5RJT2 | 0.3942 | 3.52E-03 | 0.2632 | 4.19E-02 |
| Q5FVC7 | -0.5017 | 8.95E-03 | -0.5678 | 5.08E-03 |
| P97532 | -0.5606 | 1.57E-03 | -0.2181 | 2.10E-01 |
| P15651 | -0.7317 | 1.17E-02 | -0.8528 | 4.19E-03 |
| A0A0G2K5T9 | -0.3983 | 9.56E-04 | -0.0253 | 8.26E-01 |
| Q5PPN5 | -0.2989 | 1.28E-02 | -0.2847 | 1.71E-02 |
| Q5U2U8 | -0.0890 | 4.32E-01 | -0.3747 | 1.03E-03 |
| D3ZG43 | -0.3086 | 2.87E-03 | -0.1659 | 1.08E-01 |
| D3ZRM0 | 0.3018 | 3.09E-02 | 0.4105 | 7.54E-03 |
| Q5RJN0 | -0.1515 | 3.14E-01 | -0.5015 | 1.22E-03 |
| D3ZIK1 | 0.2786 | 1.32E-01 | 0.5864 | 2.30E-03 |
| Q6AYF4 | 0.2651 | 4.18E-02 | 0.3633 | 5.65E-03 |
| B2GV41 | -0.7066 | 3.57E-03 | -0.4850 | 2.16E-02 |
| D3ZVB7 | -0.3939 | 1.48E-02 | -0.4053 | 1.38E-02 |
| B5DEK0 | -0.6561 | 3.90E-03 | -0.4250 | 2.02E-02 |
| P29315 | 0.2459 | 2.89E-02 | 0.3100 | 8.63E-03 |
| O35550 | -0.2134 | 1.14E-01 | -0.3964 | 2.57E-03 |
| P11884 | -0.6325 | 1.16E-03 | 0.0995 | 5.97E-01 |
| B0BNA5 | 0.6726 | 5.06E-02 | 1.0094 | 4.50E-03 |
| Q68FY0 | -0.4224 | 2.20E-03 | -0.1796 | 1.90E-01 |
| B4F7C7 | -0.0776 | 4.37E-01 | -0.3513 | 1.21E-03 |
| D3ZIE1 | -0.5620 | 3.24E-03 | -0.3003 | 1.12E-01 |
| D4A9T2 | -0.5403 | 5.58E-03 | -0.4794 | 1.25E-02 |
| D3ZSG3 | -0.9665 | 1.71E-02 | -1.0145 | 1.29E-02 |
| P40307 | 0.6603 | 1.55E-02 | 0.6460 | 1.50E-02 |
| D3ZWN1 | 0.5283 | 2.70E-02 | 0.6395 | 8.47E-03 |
| D3ZWJ1 | -0.2336 | 1.17E-01 | -0.4504 | 2.80E-03 |
| P81795 | -0.3862 | 3.18E-02 | -0.4969 | 7.44E-03 |
| D3ZCQ9 | -0.3738 | 2.93E-03 | -0.2096 | 1.23E-01 |
| P62749 | 0.2489 | 1.35E-01 | 0.5129 | 2.67E-03 |
| O35878 | -0.3372 | 1.23E-02 | -0.3148 | 2.41E-02 |
| M0R965 | 0.4241 | 9.86E-02 | 0.7991 | 1.56E-03 |
| O70511 | -0.1450 | 2.59E-01 | -0.3878 | 1.64E-03 |
| Q9JI03 | 0.2658 | 1.14E-01 | 0.5013 | 3.13E-03 |
| Q5XI21 | -0.3087 | 4.30E-02 | -0.4089 | 6.87E-03 |
| Q9JLT0 | 0.2734 | 3.21E-02 | 0.3341 | 9.25E-03 |
| F1LVX1 | 0.7852 | 1.98E-03 | 0.2354 | 1.89E-01 |
| P62845 | -0.4034 | 5.80E-02 | -0.6896 | 1.99E-03 |
| D4ACG8 | -0.3970 | 2.55E-02 | -0.4505 | 1.20E-02 |
| Q00969 | 0.3273 | 1.68E-02 | 0.3646 | 1.11E-02 |
| P10959 | 0.5814 | 1.32E-01 | 1.1924 | 2.67E-03 |
| P63025 | -0.2065 | 2.08E-01 | -0.4953 | 1.85E-03 |
| D4AEG6 | -0.8045 | 8.28E-03 | -0.6439 | 3.43E-02 |
| F1M0R1 | -0.1533 | 2.39E-01 | 0.4175 | 1.61E-03 |
| P0C0S7 | -0.4467 | 3.02E-02 | -0.5189 | 1.06E-02 |
| Q02253 | -0.2638 | 7.04E-03 | -0.1884 | 5.32E-02 |
| P05197 | 0.0892 | 5.29E-01 | 0.4186 | 1.19E-03 |
| F6T071 | 0.2299 | 2.40E-01 | 0.6088 | 2.05E-03 |
| O55171 | -0.4343 | 4.51E-03 | -0.2482 | 9.90E-02 |
| P13471 | -0.0302 | 8.62E-01 | -0.5212 | 1.10E-03 |
| D4ADF5 | -0.1275 | 1.47E-02 | -0.1167 | 2.43E-02 |
| A0A0G2QC02 | -0.6572 | 1.80E-03 | -0.2849 | 1.87E-01 |
| A0A0G2JXT8 | -0.3595 | 1.34E-01 | 0.7718 | 2.59E-03 |
| F1LSC3 | -0.1751 | 2.05E-02 | -0.1793 | 1.73E-02 |
| P02770 | 0.7633 | 4.47E-02 | 1.0552 | 8.15E-03 |
| P80349 | 0.3608 | 8.10E-02 | 0.5689 | 4.73E-03 |
| F1M9Y9 | -0.1734 | 5.63E-02 | -0.2421 | 5.60E-03 |
| P01946 | 0.0636 | 8.92E-01 | -1.3464 | 1.09E-03 |
| P22002 | -0.7596 | 1.36E-02 | -0.8491 | 7.59E-03 |
| Q641X2 | 0.2486 | 1.41E-02 | 0.2505 | 1.63E-02 |
| P61983 | 0.5789 | 2.26E-02 | 0.6215 | 1.66E-02 |
| F1M6Q3 | -0.1407 | 1.86E-01 | -0.3269 | 2.70E-03 |
| Q9JLZ1 | 0.1950 | 3.58E-01 | 0.9069 | 1.62E-03 |
| Q9R063 | -0.2711 | 2.26E-02 | -0.2959 | 1.69E-02 |
| D3ZGX8 | 0.2907 | 1.08E-01 | 0.5268 | 3.70E-03 |
| Q9WUF4 | 0.1932 | 2.92E-01 | 0.5644 | 2.04E-03 |
| Q5BJZ4 | 0.2801 | 1.41E-01 | 0.5551 | 3.36E-03 |
| P52555 | 0.3203 | 3.75E-02 | 0.3944 | 1.04E-02 |
| P63100 | -0.3486 | 6.22E-03 | -0.2340 | 7.41E-02 |
| P11980 | 0.7225 | 6.28E-03 | 0.4640 | 7.60E-02 |
| D3ZP47 | -0.3734 | 4.43E-02 | -0.4877 | 9.07E-03 |
| P05065 | -0.2859 | 1.21E-02 | -0.2342 | 3.53E-02 |
| D3ZHW1 | 0.6901 | 5.51E-03 | 0.5112 | 2.21E-02 |
| P07335 | -0.2057 | 2.03E-02 | -0.2169 | 2.02E-02 |
| O88656 | 0.2202 | 2.70E-01 | 0.5945 | 2.18E-03 |
| Q05175 | 0.5118 | 1.39E-02 | 0.4434 | 3.06E-02 |
| D3ZUV3 | -0.5148 | 6.88E-03 | -0.3520 | 6.86E-02 |
| P62856 | -0.4876 | 1.31E-02 | -0.4200 | 3.30E-02 |
| P05426 | -0.3916 | 3.16E-02 | -0.5486 | 4.58E-03 |
| Q62658 | -0.2646 | 8.99E-03 | -0.1970 | 5.31E-02 |
| P46462 | 0.3941 | 9.68E-03 | 0.3026 | 4.93E-02 |
| Q4V893 | 0.0875 | 2.82E-01 | 0.2376 | 2.22E-03 |
| A0A0G2JW60 | -0.2574 | 3.02E-03 | -0.0995 | 2.49E-01 |
| Q07266 | 0.1270 | 2.45E-01 | 0.3242 | 2.62E-03 |
| D3ZBH5 | -0.2801 | 2.69E-02 | -0.3362 | 1.13E-02 |
| P15865 | -0.8754 | 1.20E-02 | -0.7289 | 4.15E-02 |
| Q499R6 | -0.1069 | 2.02E-01 | -0.2441 | 3.04E-03 |
| P19511 | -0.3137 | 5.31E-03 | -0.1832 | 1.05E-01 |
| Q6AYJ2 | -0.3468 | 1.17E-02 | -0.2791 | 4.71E-02 |
| A0A0G2K0U9 | -0.2954 | 6.00E-02 | -0.4566 | 6.14E-03 |
| P0DN35 | -0.4896 | 2.08E-02 | -0.4785 | 2.46E-02 |
| D3ZGR7 | 0.5355 | 2.00E-02 | 0.5150 | 2.57E-02 |
| Q566E4 | -0.7851 | 5.54E-03 | -0.4716 | 8.27E-02 |
| A0A0G2K0V8 | 0.1408 | 1.73E-01 | 0.2985 | 3.80E-03 |
| B2RZB5 | -0.3087 | 7.15E-03 | -0.1976 | 8.70E-02 |
| Q7M0E3 | -0.2229 | 5.43E-02 | -0.3093 | 8.97E-03 |
| Q99PS8 | 0.2486 | 1.34E-01 | 0.4688 | 4.75E-03 |
| Q4V8H8 | -0.5201 | 2.64E-03 | -0.1290 | 4.50E-01 |
| Q4V8C3 | -0.1964 | 1.54E-01 | -0.3899 | 4.37E-03 |
| O88553 | -0.2916 | 8.41E-02 | -0.4542 | 6.32E-03 |
| A0A0G2K8V3 | 0.5075 | 3.25E-02 | 0.5955 | 1.50E-02 |
| P04636 | 0.3569 | 1.56E-02 | 0.3137 | 3.76E-02 |
| D3ZAS1 | -0.4174 | 2.00E-02 | -0.3952 | 2.92E-02 |
| Q9WTV5 | -0.1244 | 1.44E-01 | -0.2379 | 4.77E-03 |
| Q9ESW0 | 0.6972 | 7.01E-02 | 1.0793 | 6.46E-03 |
| P08460 | -0.6400 | 8.21E-03 | -0.4054 | 8.83E-02 |
| D4A517 | 0.6320 | 1.48E-02 | 0.6357 | 1.40E-02 |
| O88778 | -0.5406 | 3.45E-02 | -0.6707 | 1.21E-02 |
| Q6TRW4 | 0.1764 | 5.53E-01 | 0.8525 | 2.02E-03 |
| P60711 | 0.3772 | 2.22E-01 | 0.8911 | 3.80E-03 |
| P30009 | 0.5716 | 2.69E-02 | 0.5918 | 2.41E-02 |
| C0JPT7 | 0.2549 | 1.78E-01 | 0.5427 | 4.54E-03 |
| P21913 | -0.4843 | 1.24E-02 | -0.3838 | 5.61E-02 |
| P22509 | -0.6429 | 6.99E-03 | -0.3791 | 1.05E-01 |
| D4A6E8 | -0.1904 | 6.13E-03 | -0.0976 | 1.46E-01 |
| D3Z9Z0 | -0.2603 | 3.11E-01 | -0.7187 | 3.02E-03 |
| Q8K3F3 | 0.2118 | 1.22E-01 | 0.3724 | 6.20E-03 |
| Q6MGD0 | -0.4288 | 2.83E-02 | -0.4369 | 2.40E-02 |
| Q5U3Z7 | 0.5406 | 9.00E-02 | 1.0679 | 4.30E-03 |
| P0C1X8 | -0.1143 | 8.52E-02 | -0.1726 | 8.52E-03 |
| A0A0G2K7M2 | -0.1578 | 3.47E-01 | -0.4665 | 2.91E-03 |
| P41542 | 0.1412 | 6.56E-02 | 0.1930 | 1.08E-02 |
| D3ZE02 | 0.6523 | 6.17E-03 | 0.3589 | 1.35E-01 |
| Q9WTQ2 | -0.1191 | 2.90E-01 | -0.3127 | 3.31E-03 |
| F1M6L8 | -0.2244 | 4.78E-03 | -0.0999 | 2.25E-01 |
| Q2MJT0 | -0.6234 | 2.83E-03 | -0.0826 | 6.77E-01 |
| D3ZH75 | -0.1676 | 8.54E-02 | -0.2601 | 9.35E-03 |
| P56574 | -0.4346 | 8.04E-03 | -0.2697 | 1.14E-01 |
| Q63396 | -0.5551 | 1.47E-02 | -0.4548 | 3.96E-02 |
| B2RYS8 | -0.3658 | 8.92E-03 | -0.2219 | 1.08E-01 |
| Q7TQ85 | -0.4279 | 2.41E-02 | -0.4039 | 2.86E-02 |
| B0BNM1 | 0.4066 | 1.17E-02 | 0.3079 | 5.39E-02 |
| A0A0G2JZ38 | 0.1915 | 2.59E-01 | 0.4936 | 3.50E-03 |
| D4A8I8 | -0.1977 | 2.19E-01 | 0.5245 | 4.33E-03 |
| Q91ZV2 | -0.2510 | 1.27E-01 | -0.4291 | 5.38E-03 |
| M0R5V6 | 0.2295 | 2.08E-01 | 0.4936 | 4.75E-03 |
| D3Z937 | -0.4432 | 1.63E-02 | -0.3772 | 1.92E-02 |
| P18886 | -0.4316 | 1.07E-02 | -0.2949 | 6.77E-02 |
| F1M265 | 0.4645 | 1.99E-02 | 0.4529 | 1.61E-02 |
| Q62847 | -0.2922 | 1.62E-02 | -0.2261 | 5.79E-02 |
| Q5XIM9 | 0.4435 | 7.90E-02 | 0.6383 | 1.14E-02 |
| P14408 | -0.3326 | 4.03E-03 | -0.1136 | 3.58E-01 |
| F1LN92 | -0.2807 | 8.67E-02 | -0.4431 | 9.71E-03 |
| P62775 | 0.0408 | 7.05E-01 | 0.3063 | 2.57E-03 |
| P48675 | 0.1619 | 1.32E-01 | 0.3125 | 8.01E-03 |
| B2RYN8 | -0.3374 | 5.34E-02 | -0.4141 | 1.68E-02 |
| D3ZD97 | 0.0732 | 6.31E-01 | 0.4142 | 2.59E-03 |
| Q9R0L4 | -0.3126 | 8.11E-02 | -0.4527 | 1.17E-02 |
| G3V629 | 0.4353 | 4.10E-02 | 0.4884 | 2.21E-02 |
| A0A0G2K1W1 | -0.1688 | 4.46E-02 | -0.1907 | 2.05E-02 |
| Q8VIL3 | 0.6217 | 6.82E-03 | 0.5756 | 5.89E-02 |
| A0A1W2Q6F8 | -0.3694 | 1.42E-01 | -0.6983 | 6.08E-03 |
| M0R9Z5 | 0.2106 | 4.56E-02 | 0.2585 | 2.06E-02 |
| P50411 | -0.1041 | 2.73E-01 | -0.2567 | 4.51E-03 |
| A0A096MJX5 | 0.1180 | 2.26E-01 | 0.2715 | 5.43E-03 |
| G3V968 | 0.2839 | 3.28E-02 | 0.3273 | 2.05E-02 |
| A0A0G2K0X1 | -0.3051 | 2.77E-02 | -0.2830 | 3.60E-02 |
| Q8K3R4 | 0.4478 | 5.86E-02 | 0.5636 | 1.68E-02 |
| D3ZLC1 | -0.1268 | 1.64E-01 | -0.2832 | 6.15E-03 |
| Q5RKH1 | 0.6655 | 3.81E-02 | 0.7228 | 2.32E-02 |
| Q5M9G1 | -0.1945 | 3.74E-02 | -0.2087 | 2.63E-02 |
| A0A096MIZ1 | 0.1956 | 3.05E-03 | -0.0001 | 9.99E-01 |
| Q9QZK8 | 0.4978 | 1.80E-02 | 0.3984 | 5.47E-02 |
| D3ZLD7 | -0.3401 | 3.74E-02 | -0.4934 | 1.19E-02 |
| Q9QX67 | -0.1717 | 1.45E-01 | -0.3063 | 8.39E-03 |
| Q5EB95 | -0.5934 | 7.13E-03 | -0.2808 | 1.97E-01 |
| P70619 | 0.4228 | 4.36E-02 | 0.4522 | 2.23E-02 |
| P34926 | 0.1775 | 1.54E-02 | 0.1257 | 8.11E-02 |
| D3ZPF0 | -0.2525 | 4.12E-02 | -0.2701 | 2.63E-02 |
| Q2PQA9 | -0.1787 | 3.94E-02 | -0.1927 | 2.80E-02 |
| O35806 | 0.7377 | 7.94E-03 | 0.3577 | 1.94E-01 |
| P20760 | 0.4735 | 1.77E-01 | 0.9290 | 7.49E-03 |
| B2RZ29 | 0.3104 | 2.87E-02 | 0.2932 | 4.05E-02 |
| D3Z9K4 | 0.0373 | 7.27E-01 | 0.2893 | 3.12E-03 |
| Q5XIB5 | -0.2210 | 3.96E-02 | -0.2288 | 2.82E-02 |
| D3ZKK3 | -0.3772 | 3.07E-02 | -0.3950 | 2.64E-02 |
| P00406 | -0.4982 | 1.83E-02 | -0.4802 | 2.64E-02 |
| G3V7K1 | -0.2840 | 1.54E-02 | -0.2049 | 8.70E-02 |
| Q3SWU3 | 0.0712 | 4.42E-01 | 0.2538 | 4.19E-03 |
| B5DFK2 | 0.6337 | 4.13E-02 | 0.6943 | 2.64E-02 |
| D3ZCI9 | 0.0364 | 8.35E-01 | 0.4715 | 3.28E-03 |
| D3ZSL2 | 0.1937 | 3.94E-01 | -0.7590 | 4.22E-03 |
| F1M614 | -0.2119 | 7.29E-02 | -0.2759 | 1.76E-02 |
| P18418 | 0.2277 | 3.76E-01 | 0.6988 | 4.85E-03 |
| P27139 | 0.1000 | 6.81E-01 | 0.6943 | 3.69E-03 |
| D3ZCI3 | -0.4669 | 1.91E-02 | -0.4033 | 3.00E-02 |
| Q811U3 | -0.2034 | 2.15E-02 | -0.1657 | 6.46E-02 |
| Q9R066 | -1.3974 | 7.12E-03 | -0.5261 | 1.91E-01 |
| Q01129 | -0.4570 | 9.11E-03 | -0.2295 | 1.94E-01 |
| Q9WTT7 | -0.3637 | 4.09E-02 | -0.5033 | 1.47E-02 |
| D4A206 | 0.1816 | 1.87E-01 | 0.3690 | 8.53E-03 |
| A0A0G2JX92 | -0.4426 | 1.91E-02 | -0.3167 | 3.27E-02 |
| P13413 | -0.3807 | 1.64E-01 | -0.7173 | 7.75E-03 |
| I6L9G5 | 0.2213 | 6.02E-03 | 0.0746 | 3.69E-01 |
| B0K008 | 0.3261 | 5.75E-02 | 0.3875 | 2.41E-02 |
| A0A0G2K6I4 | 0.1741 | 2.07E-01 | 0.3774 | 8.30E-03 |
| P08934 | 0.1836 | 1.69E-01 | 0.3448 | 9.74E-03 |
| D3ZK97 | -0.4176 | 6.83E-02 | -0.5368 | 2.00E-02 |
| D3Z914 | -0.1220 | 3.52E-01 | 0.3458 | 5.52E-03 |
| F1M2E9 | -0.3239 | 1.13E-01 | -0.4697 | 1.39E-02 |
| B2B9A9 | -0.0439 | 8.31E-01 | -0.6224 | 4.13E-03 |
| P14841 | 0.4044 | 6.17E-03 | 0.1433 | 3.82E-01 |
| A0A0G2JWR5 | -0.3504 | 2.42E-02 | -0.3127 | 6.14E-02 |
| Q9Z2P6 | 0.1613 | 3.59E-02 | 0.1545 | 4.40E-02 |
| M0R629 | -0.3896 | 1.43E-02 | -0.2498 | 1.19E-01 |
| D4AC73 | -0.4178 | 8.69E-02 | -0.7260 | 9.71E-03 |
| B2RYL3 | 0.3916 | 2.91E-01 | 0.9585 | 6.60E-03 |
| E9PT37 | -0.2610 | 7.51E-02 | -0.3367 | 2.18E-02 |
| Q9QX79 | 0.2106 | 1.59E-01 | 0.3865 | 1.19E-02 |
| D3ZYW7 | -0.1271 | 4.43E-01 | -0.4421 | 5.51E-03 |
| P00787 | 0.1893 | 1.64E-01 | 0.3352 | 1.13E-02 |
| O08837 | 0.0553 | 5.57E-01 | 0.2510 | 4.96E-03 |
| F1LMK8 | 0.6305 | 2.55E-02 | 0.5801 | 3.02E-02 |
| P26644 | 0.1099 | 3.20E-01 | 0.3033 | 7.29E-03 |
| D3Z9M5 | 0.2615 | 9.35E-03 | 0.1119 | 2.58E-01 |
| Q99NA5 | -0.3689 | 1.47E-02 | -0.2266 | 1.39E-01 |
| P35467 | -0.0941 | 6.31E-01 | -0.5086 | 4.70E-03 |
| D4A0Z4 | -0.4666 | 1.14E-02 | -0.2451 | 1.15E-01 |
| Q62698 | 0.2121 | 1.08E-01 | 0.3262 | 1.63E-02 |
| Q6AY90 | 0.1114 | 2.12E-01 | 0.2263 | 9.77E-03 |
| P0C5W1 | 0.1575 | 4.02E-01 | 0.4876 | 6.15E-03 |
| Q62839 | 0.2328 | 1.72E-01 | 0.4166 | 1.17E-02 |
| P97675 | 0.2145 | 2.69E-01 | 0.5329 | 7.49E-03 |
| P32551 | -0.3185 | 9.46E-03 | -0.1366 | 2.73E-01 |
| D3ZQL6 | -0.2018 | 1.17E-01 | -0.3351 | 1.40E-02 |
| D3ZLS5 | -0.4412 | 1.23E-02 | -0.2231 | 1.99E-01 |
| P24368 | -0.2511 | 2.71E-02 | -0.2108 | 7.37E-02 |
| G3V9W0 | -0.2580 | 1.12E-01 | -0.3700 | 1.79E-02 |
| D3ZUL3 | -0.2239 | 3.24E-02 | -0.1965 | 6.14E-02 |
| D3Z9K2 | -0.0520 | 7.24E-01 | -0.3748 | 4.82E-03 |
| A0A1W2Q642 | -0.7068 | 1.13E-02 | -0.3160 | 2.10E-01 |
| G3V6A6 | -0.6371 | 2.99E-02 | -0.4991 | 6.53E-02 |
| B2RYA8 | -0.2636 | 9.78E-02 | -0.3703 | 2.17E-02 |
| P08699 | 0.7491 | 6.85E-02 | 0.9212 | 2.72E-02 |
| Q63068 | 0.1665 | 2.21E-01 | 0.3358 | 1.09E-02 |
| Q9ER30 | 0.4529 | 1.04E-01 | 0.7506 | 1.21E-02 |
| G3V9R0 | 0.0415 | 8.74E-01 | 0.8194 | 5.65E-03 |
| P34067 | 0.4911 | 3.85E-02 | 0.4509 | 5.49E-02 |
| A0A0G2K7W4 | -0.0147 | 9.25E-01 | 0.3611 | 4.58E-03 |
| A0A0G2JY73 | -0.3273 | 1.62E-02 | -0.1959 | 1.29E-01 |
| A0A0G2JUA5 | 0.1103 | 3.83E-01 | 0.3225 | 7.49E-03 |
| F1M8L9 | -0.0557 | 8.32E-01 | 0.7340 | 5.43E-03 |
| Q5XIC8 | -0.4352 | 2.11E-02 | -0.3517 | 5.49E-02 |
| P35171 | 0.1936 | 2.73E-01 | 0.4470 | 9.72E-03 |
| D3ZKG1 | -0.6159 | 3.02E-02 | -0.5153 | 6.03E-02 |
| F7FAY5 | 0.0942 | 5.81E-01 | 0.5395 | 6.37E-03 |
| Q9JIH7 | 0.1853 | 1.96E-02 | 0.1183 | 1.32E-01 |
| B5DF65 | -0.5395 | 7.94E-03 | -0.1187 | 4.86E-01 |
| P53565 | 0.5983 | 1.50E-02 | 0.3296 | 1.59E-01 |
| Q62703 | 0.1463 | 1.43E-01 | 0.2393 | 1.74E-02 |
| Q5XIM5 | 0.2140 | 9.30E-02 | 0.2785 | 2.49E-02 |
| P18420 | 0.2898 | 6.45E-02 | 0.3387 | 3.59E-02 |
| P56571 | -0.1453 | 4.65E-01 | -0.5066 | 7.27E-03 |
| Q99ME0 | -1.4146 | 1.60E-02 | -0.8425 | 1.01E-01 |
| Q66H39 | 0.2949 | 7.13E-03 | 0.0537 | 6.29E-01 |
| B0BNB9 | -0.4747 | 2.13E-02 | -0.3486 | 6.28E-02 |
| P21961 | -0.3830 | 4.56E-02 | -0.4003 | 4.72E-02 |
| P60901 | 0.4202 | 4.03E-02 | 0.3796 | 5.98E-02 |
| F1LTF8 | -0.1617 | 1.23E-01 | -0.2394 | 2.05E-02 |
| Q4QQV4 | -0.1991 | 1.50E-01 | -0.3239 | 1.74E-02 |
| D3ZD05 | -0.4801 | 8.00E-02 | -0.4832 | 1.45E-02 |
| F1LWS4 | 0.1189 | 3.53E-01 | 0.3202 | 9.15E-03 |
| P30919 | -0.0901 | 4.80E-01 | -0.3224 | 7.59E-03 |
| Q00438 | 0.2051 | 2.75E-01 | 0.4694 | 1.12E-02 |
| Q9ES53 | -0.4228 | 3.66E-02 | -0.3560 | 7.18E-02 |
| Q9ES73 | 0.3671 | 2.97E-02 | 0.2933 | 8.48E-02 |
| P04041 | -0.1699 | 3.59E-01 | -0.4567 | 9.30E-03 |
| F1LR02 | 0.3481 | 1.66E-01 | 0.5859 | 1.69E-02 |
| G3V798 | 0.3319 | 2.92E-02 | 0.2456 | 9.78E-02 |
| Q923W4 | -0.2116 | 1.51E-01 | -0.3247 | 1.84E-02 |
| Q63617 | 0.2516 | 9.90E-02 | 0.3345 | 2.71E-02 |
| P11232 | -0.1802 | 2.95E-01 | -0.4349 | 1.14E-02 |
| O08629 | 0.2137 | 1.96E-01 | 0.3974 | 1.55E-02 |
| Q4KLG3 | 0.4832 | 3.88E-02 | 0.3855 | 3.59E-02 |
| Q5XFX0 | 0.0782 | 5.50E-01 | 0.3375 | 7.74E-03 |
| Q6AY72 | 0.3653 | 4.22E-02 | 0.3180 | 6.54E-02 |
| D3ZX38 | 0.0837 | 2.35E-01 | 0.1702 | 1.37E-02 |
| F1M3K6 | 0.4891 | 1.15E-02 | 0.1500 | 3.30E-01 |
| P0C219 | -0.1840 | 1.05E-01 | -0.2448 | 2.70E-02 |
| Q5PPG6 | -1.3923 | 2.12E-02 | -0.9455 | 8.61E-02 |
| Q5XI73 | 0.0651 | 6.57E-01 | 0.3624 | 7.29E-03 |
| B0BMX3 | -0.2629 | 3.86E-02 | -0.2446 | 6.14E-02 |
| Q5PQP8 | 0.5589 | 8.28E-03 | 0.1759 | 4.83E-01 |
| Q6AYT4 | -0.1668 | 1.66E-01 | -0.2822 | 2.00E-02 |
| E9PTB2 | -0.5603 | 1.05E-02 | -0.1137 | 5.23E-01 |
| P82995 | 0.3479 | 8.69E-02 | 0.4339 | 3.55E-02 |
| D3ZXP3 | -0.6219 | 2.96E-02 | -0.4418 | 1.16E-01 |
| P84079 | -0.0706 | 7.07E-01 | 0.4552 | 7.41E-03 |
| M0RA79 | 0.7213 | 2.86E-02 | 0.5838 | 6.86E-02 |
| P62083 | 0.3099 | 1.43E-01 | 0.4699 | 2.42E-02 |
| P02563 | -0.2412 | 1.38E-01 | -0.3712 | 2.59E-02 |
| Q8CHJ4 | -0.4730 | 2.76E-02 | -0.3216 | 1.17E-01 |
| A0A0G2K9C0 | 0.2763 | 1.87E-01 | 0.4871 | 2.03E-02 |
| A0A0G2JUX4 | -0.5334 | 2.07E-02 | -0.3172 | 1.26E-01 |
| F1LNF0 | -0.1606 | 1.27E-01 | -0.2254 | 2.64E-02 |
| Q9Z2Q1 | 0.2199 | 1.25E-01 | 0.3107 | 2.92E-02 |
| P28077 | -0.1236 | 5.50E-01 | 0.5772 | 9.57E-03 |
| A0A0G2K719 | 0.2708 | 1.65E-01 | 0.4369 | 2.35E-02 |
| Q8K585 | -0.2806 | 1.83E-02 | -0.1443 | 2.44E-01 |
| Q641Y2 | -0.3341 | 2.04E-02 | -0.1874 | 1.86E-01 |
| Q5XII0 | -0.1674 | 1.68E-01 | -0.2883 | 2.41E-02 |
| Q6XKD3 | -0.5266 | 8.69E-02 | -0.7185 | 2.50E-02 |
| A0A0G2K9Z7 | -0.2900 | 8.47E-02 | -0.3284 | 4.37E-02 |
| P00173 | -0.2436 | 2.48E-01 | -0.4697 | 1.67E-02 |
| Q91ZN1 | 0.0170 | 9.65E-01 | 0.8777 | 7.71E-03 |
| D3ZCG4 | -0.1453 | 1.86E-01 | -0.2486 | 2.17E-02 |
| Q6P0K8 | -0.2730 | 1.14E-01 | -0.3570 | 3.38E-02 |
| A0A0G2JZF6 | -0.2212 | 3.39E-02 | -0.1804 | 9.59E-02 |
| O35854 | 0.8735 | 1.07E-02 | 0.0488 | 8.83E-01 |
| P70531 | 0.5386 | 1.83E-01 | 1.0568 | 1.64E-02 |
| Q3KRE0 | -0.2257 | 7.28E-02 | -0.2427 | 4.92E-02 |
| Q6AZ50 | 0.3906 | 9.89E-02 | 0.4806 | 3.96E-02 |
| A0A0G2K8N1 | -0.4186 | 3.94E-02 | -0.3502 | 1.02E-01 |
| D3ZHM7 | 0.0978 | 2.43E-01 | 0.1846 | 1.78E-02 |
| P34900 | 0.2513 | 2.46E-01 | 0.6187 | 1.44E-02 |
| Q6AYK5 | 0.0542 | 7.67E-01 | 0.4230 | 8.97E-03 |
| Q6AY21 | -0.2606 | 1.53E-02 | 0.0908 | 3.97E-01 |
| P11598 | 0.1421 | 1.78E-01 | 0.2376 | 2.52E-02 |
| D3ZY47 | -0.1664 | 1.76E-01 | -0.2716 | 2.54E-02 |
| Q63014 | -0.2356 | 2.39E-01 | 0.5956 | 1.66E-02 |
| Q794E4 | 0.1214 | 5.83E-01 | 0.5129 | 1.08E-02 |
| P58200 | -0.2810 | 2.89E-02 | -0.1812 | 1.60E-01 |
| B2RZ69 | -0.1331 | 3.56E-01 | -0.3399 | 1.41E-02 |
| Q4V8A6 | -0.2189 | 3.91E-01 | -0.7955 | 1.37E-02 |
| Q5U302 | -0.5578 | 5.63E-02 | -0.4969 | 7.35E-02 |
| D3ZJ96 | -0.4910 | 1.90E-02 | -0.2197 | 2.92E-01 |
| Q4KM98 | -0.3208 | 1.01E-01 | -0.3776 | 4.36E-02 |
| Q6F6B3 | 0.0682 | 7.04E-01 | -0.4243 | 1.02E-02 |
| D4A500 | -0.2141 | 3.68E-01 | -0.5803 | 1.58E-02 |
| A0A0G2KAM4 | -0.3094 | 2.89E-02 | -0.1817 | 1.55E-01 |
| A0A0U1RRT8 | -0.1403 | 2.80E-01 | -0.3227 | 1.72E-02 |
| D4A9T0 | -0.3647 | 2.59E-02 | -0.2205 | 1.74E-01 |
| P02650 | 0.4291 | 2.04E-01 | 0.7596 | 2.41E-02 |
| P38983 | 0.1974 | 3.97E-01 | 0.5722 | 1.40E-02 |
| P62329 | -0.5508 | 2.51E-02 | -0.3034 | 2.19E-01 |
| Q6MG88 | 0.3667 | 1.04E-01 | 0.4437 | 4.54E-02 |
| Q5XIP0 | 0.0387 | 8.70E-01 | -0.5309 | 9.64E-03 |
| Q64303 | 0.3422 | 4.34E-02 | 0.2726 | 9.61E-02 |
| P04631 | 0.0125 | 9.60E-01 | -0.4987 | 9.31E-03 |
| P48199 | 0.5986 | 8.00E-02 | 0.6469 | 6.17E-02 |
| A0A0G2K2W2 | -0.6735 | 1.86E-01 | -1.1222 | 2.65E-02 |
| O70535 | -0.2444 | 2.54E-01 | -0.4782 | 2.17E-02 |
| P38656 | -0.5379 | 2.49E-02 | -0.2700 | 2.48E-01 |
| P48500 | -0.1872 | 3.80E-01 | -0.5062 | 1.68E-02 |
| P23562 | -0.7154 | 1.32E-01 | -0.9054 | 3.75E-02 |
| B2GV99 | 0.0619 | 6.91E-01 | 0.3474 | 1.15E-02 |
| Q9WVR3 | -0.0826 | 7.03E-01 | -0.5829 | 1.27E-02 |
| A0A0G2K4D5 | -0.5123 | 4.71E-02 | -0.4627 | 6.79E-02 |
| Q4KLZ0 | 0.3088 | 2.05E-01 | 0.5157 | 2.66E-02 |
| D3ZTA8 | 0.2563 | 1.15E-01 | 0.3329 | 4.03E-02 |
| D3ZF26 | -0.1645 | 3.11E-02 | -0.0985 | 1.94E-01 |
| Q4KM38 | -0.4358 | 1.39E-02 | -0.0671 | 7.13E-01 |
| Q63507 | -0.2709 | 1.20E-01 | -0.3467 | 4.57E-02 |
| Q63624 | -0.4080 | 3.09E-02 | -0.2265 | 1.38E-01 |
| P15999 | -0.2406 | 1.96E-02 | -0.0918 | 3.79E-01 |
| D3ZC56 | -0.0648 | 5.63E-01 | -0.2473 | 1.33E-02 |
| D4A7R0 | 0.2192 | 2.99E-01 | 0.5671 | 1.80E-02 |
| A0A0G2K6F9 | 0.1966 | 1.91E-01 | 0.3173 | 3.02E-02 |
| P50503 | -0.1634 | 8.25E-02 | -0.1706 | 6.79E-02 |
| Q562A2 | -0.1816 | 3.09E-01 | -0.4590 | 1.80E-02 |
| O35244 | -0.1703 | 1.48E-01 | -0.2408 | 3.95E-02 |
| Q6P9V9 | 0.1376 | 5.45E-01 | 0.5103 | 1.44E-02 |
| Q5QD51 | -0.1661 | 1.89E-02 | -0.0588 | 4.24E-01 |
| Q05695 | -0.2630 | 1.89E-02 | -0.0948 | 4.02E-01 |
| D4A2F6 | -0.6223 | 1.83E-02 | -0.0952 | 6.29E-01 |
| F1M7L9 | -0.2622 | 2.71E-01 | -0.5500 | 2.21E-02 |
| F1M7M4 | -0.3541 | 1.43E-02 | -0.0458 | 7.72E-01 |
| Q642C0 | 0.0927 | 3.28E-01 | 0.2117 | 2.12E-02 |
| D4AE96 | 0.2620 | 2.37E-02 | 0.1249 | 3.02E-01 |
| P24049 | -0.3195 | 1.17E-01 | -0.3809 | 5.16E-02 |
| D3Z874 | -0.2025 | 1.09E-01 | -0.2489 | 5.74E-02 |
| Q9EPH1 | -0.2461 | 1.59E-01 | 0.3617 | 4.04E-02 |
| A0A0G2K782 | -0.1356 | 4.35E-01 | -0.4281 | 1.80E-02 |
| Q5XI26 | -0.4389 | 1.71E-02 | -0.0582 | 7.27E-01 |
| P20695 | 1.0181 | 5.66E-02 | 0.9386 | 7.08E-02 |
| F1LNJ2 | 0.1525 | 4.17E-01 | 0.4276 | 1.91E-02 |
| D3ZQM0 | 0.2789 | 1.42E-01 | 0.3931 | 4.37E-02 |
| F1M8P4 | -0.7654 | 4.95E-02 | -0.7071 | 7.84E-02 |
| A0A0G2K0J7 | 0.1540 | 7.04E-01 | 0.9382 | 1.43E-02 |
| F1LTN6 | -0.4724 | 3.92E-01 | -1.2464 | 2.03E-02 |
| B5DFM8 | -0.2884 | 2.94E-01 | -0.6917 | 2.58E-02 |
| B4F789 | -0.3166 | 5.43E-02 | -0.2539 | 1.24E-01 |
| Q6AY80 | -0.4208 | 8.67E-02 | -0.4711 | 4.83E-02 |
| A0A0G2JTL6 | -0.2396 | 6.19E-02 | -0.2071 | 1.09E-01 |
| P51868 | -0.6035 | 1.89E-02 | 0.1765 | 5.17E-01 |
| P51886 | -0.5782 | 2.89E-02 | -0.3076 | 2.71E-01 |
| Q99ND9 | 0.1681 | 4.29E-01 | 0.4930 | 2.00E-02 |
| P86252 | -0.3029 | 9.94E-02 | -0.3304 | 6.56E-02 |
| Q63621 | 0.2062 | 2.33E-01 | 0.3571 | 3.10E-02 |
| Q8VIF7 | 0.0916 | 6.69E-01 | -0.4516 | 1.47E-02 |
| Q3B7D0 | 0.6448 | 2.08E-02 | 0.1998 | 4.67E-01 |
| D3ZDN9 | 0.5321 | 2.06E-02 | -0.1360 | 4.95E-01 |
| D4A4A9 | -0.6276 | 3.75E-02 | -0.4393 | 1.30E-01 |
| G3V783 | 0.2246 | 2.08E-01 | 0.3812 | 2.59E-02 |
| Q3V5X8 | -0.6723 | 2.82E-02 | -0.3082 | 2.49E-01 |
| A0A0G2K1E2 | 0.0209 | 8.49E-01 | 0.2361 | 1.43E-02 |
| P63329 | -0.4497 | 1.21E-01 | -0.5956 | 4.08E-02 |
| Q6AY97 | -0.1719 | 2.87E-02 | -0.0774 | 3.19E-01 |
| D3ZS15 | -0.4590 | 7.27E-02 | -0.4081 | 1.02E-01 |
| D4A5T1 | -0.2127 | 2.26E-01 | -0.4612 | 2.80E-02 |
| Q27W01 | 0.0407 | 6.08E-01 | 0.1712 | 1.67E-02 |
| O08839 | 0.0933 | 4.99E-01 | 0.3139 | 1.98E-02 |
| D4A8M5 | -0.1991 | 6.82E-02 | -0.1766 | 9.58E-02 |
| Q62881 | -0.0375 | 7.54E-01 | -0.2585 | 1.56E-02 |
| D4A305 | -0.1394 | 5.49E-01 | -0.5333 | 1.94E-02 |
| Q9QYN5 | 0.5297 | 2.44E-02 | 0.1737 | 4.02E-01 |
| M0R7Z0 | 0.1439 | 2.43E-01 | 0.2509 | 3.38E-02 |
| P63039 | -0.2315 | 8.69E-02 | -0.2279 | 8.98E-02 |
| Q5XIW8 | -0.0416 | 5.78E-01 | 0.1566 | 1.78E-02 |
| A0A0G2JXF0 | -0.3953 | 2.31E-02 | -0.1009 | 4.98E-01 |
| O70377 | -0.2333 | 6.17E-02 | -0.1855 | 1.25E-01 |
| G3V9N7 | -0.1068 | 3.76E-01 | -0.2525 | 2.41E-02 |
| Q8K4T4 | -0.4449 | 7.27E-02 | -0.4009 | 1.12E-01 |
| Q4KLH4 | -0.4101 | 4.67E-02 | -0.2551 | 1.68E-01 |
| P15429 | -0.4058 | 8.00E-02 | -0.3747 | 1.03E-01 |
| O35952 | -0.1544 | 5.15E-01 | -0.5019 | 2.02E-02 |
| B5DFN3 | -0.4909 | 1.65E-02 | -0.0147 | 9.59E-01 |
| D4ABT8 | 0.0565 | 7.04E-01 | 0.3161 | 1.74E-02 |
| P18576 | -0.4715 | 4.90E-02 | -0.3596 | 1.18E-01 |
| P20759 | 0.5942 | 1.04E-01 | 0.6301 | 8.21E-02 |
| O35787 | -0.3582 | 5.88E-02 | -0.2949 | 1.03E-01 |
| O35413 | -0.1172 | 3.34E-01 | -0.2505 | 2.90E-02 |
| A0A0G2JYY8 | -0.3089 | 2.12E-02 | 0.0531 | 6.98E-01 |
| P34058 | 0.2144 | 2.36E-01 | 0.3700 | 3.99E-02 |
| Q62764 | 0.1469 | 2.52E-01 | 0.2559 | 3.70E-02 |
| O88453 | -0.1917 | 5.89E-02 | -0.1431 | 1.59E-01 |
| Q63028 | -0.2063 | 3.47E-02 | -0.1011 | 3.10E-01 |
| M0R671 | -0.7486 | 6.02E-02 | -0.5589 | 1.09E-01 |
| P63149 | -0.4485 | 1.38E-01 | -0.6037 | 4.69E-02 |
| F1M155 | -0.0541 | 5.07E-01 | -0.1739 | 2.29E-02 |
| P47942 | 0.1498 | 1.30E-01 | 0.1857 | 7.14E-02 |
| Q05962 | -0.5171 | 1.99E-02 | 0.0317 | 9.00E-01 |
| Q5BK63 | -0.8659 | 2.51E-02 | 0.1813 | 5.97E-01 |
| F1MAA7 | -0.1739 | 8.01E-02 | -0.1460 | 1.18E-01 |
| P0C2X9 | -0.6899 | 5.80E-02 | -0.5192 | 1.47E-01 |
| P08649 | 0.1318 | 4.85E-01 | 0.3981 | 2.40E-02 |
| Q9R1Z0 | -0.7591 | 2.33E-02 | -0.1782 | 6.14E-01 |
| P68255 | 0.2682 | 5.29E-01 | 1.0447 | 2.30E-02 |
| Q9Z0W7 | 0.1047 | 6.81E-01 | 0.5526 | 2.03E-02 |
| A0A0G2K654 | -0.6191 | 6.82E-02 | -0.5209 | 1.44E-01 |
| Q9QWN8 | -0.1793 | 2.67E-01 | -0.3212 | 3.85E-02 |
| P34064 | 0.5261 | 4.09E-02 | 0.3065 | 2.62E-01 |
| Q91ZS3 | 0.3321 | 6.53E-02 | 0.2603 | 1.54E-01 |
| Q4KLF8 | 0.1958 | 4.60E-01 | 0.5444 | 2.43E-02 |
| Q9QZM5 | -0.0484 | 8.41E-01 | 0.4915 | 1.86E-02 |
| D3Z9A4 | -0.1053 | 4.18E-01 | -0.2651 | 2.72E-02 |
| D3ZGY2 | -0.4074 | 7.16E-02 | -0.3255 | 1.43E-01 |
| Q5FVH7 | 0.0677 | 6.69E-01 | 0.3228 | 2.06E-02 |
| D3ZWF5 | 0.0496 | 7.79E-01 | 0.3647 | 1.97E-02 |
| Q6IMY8 | -0.1796 | 3.94E-02 | -0.0994 | 2.87E-01 |
| A0A0G2KAN5 | 0.4999 | 4.95E-02 | 0.3150 | 2.15E-01 |
| D3ZMS1 | 0.1216 | 3.82E-01 | 0.2905 | 3.06E-02 |
| A0A0G2K160 | 0.3889 | 7.50E-02 | 0.3257 | 1.39E-01 |
| P21775 | 0.4503 | 2.12E-01 | 0.6833 | 5.00E-02 |
| B1WBS4 | -0.5391 | 2.18E-02 | 0.0532 | 8.51E-01 |
| Q6P9T8 | -0.3440 | 3.40E-02 | -0.1503 | 3.80E-01 |
| P23358 | 0.3570 | 5.67E-02 | 0.2948 | 1.71E-01 |
| Q6AXU6 | 0.2389 | 3.77E-02 | 0.1154 | 3.32E-01 |
| Q5XI67 | 0.0644 | 7.34E-01 | 0.3911 | 2.11E-02 |
| A0A0G2JVF0 | -0.1431 | 5.10E-01 | -0.4688 | 2.59E-02 |
| B2GV15 | -0.2820 | 1.12E-01 | -0.3067 | 8.70E-02 |
| D4A962 | 0.0701 | 5.78E-01 | 0.2637 | 2.47E-02 |
| P62982 | 0.2211 | 1.66E-01 | 0.2837 | 6.60E-02 |
| Q5RJK5 | -0.2327 | 6.91E-02 | 0.1775 | 1.63E-01 |
| Q9WU49 | 0.0471 | 6.82E-01 | -0.2335 | 2.22E-02 |
| Q62655 | 0.0810 | 5.44E-01 | 0.2594 | 2.46E-02 |
| P13676 | 0.2881 | 1.15E-01 | 0.2953 | 9.80E-02 |
| A0JPM9 | -0.1276 | 6.65E-02 | -0.0944 | 1.74E-01 |
| P18163 | -0.6324 | 1.14E-01 | -0.6834 | 8.88E-02 |
| Q9Z1P3 | 0.5441 | 7.63E-02 | 0.4536 | 1.38E-01 |
| Q3ZU82 | 0.1266 | 3.52E-01 | 0.2822 | 3.75E-02 |
| D3ZDW3 | 0.1461 | 9.75E-02 | 0.1399 | 1.22E-01 |
| D4A830 | -0.5737 | 4.32E-02 | -0.3224 | 2.42E-01 |
| Q4V8H5 | 0.4138 | 2.98E-02 | 0.1062 | 5.89E-01 |
| Q6IRK9 | 0.4996 | 7.13E-02 | 0.3694 | 1.74E-01 |
| F1M842 | -0.1200 | 8.09E-02 | -0.0991 | 1.53E-01 |
| Q812D1 | 0.0418 | 7.02E-01 | 0.2112 | 2.35E-02 |
| B0BMS9 | 0.6839 | 1.41E-01 | 0.8244 | 7.89E-02 |
| Q63635 | -0.1576 | 1.50E-01 | -0.1849 | 8.32E-02 |
| D3ZFB2 | 0.6462 | 1.91E-01 | 0.9212 | 6.04E-02 |
| F7FF45 | 0.1196 | 3.12E-01 | 0.2340 | 4.36E-02 |
| P07895 | -0.0593 | 6.93E-01 | -0.3136 | 2.57E-02 |
| Q64122 | -0.1514 | 3.12E-01 | -0.2879 | 4.33E-02 |
| A0A0G2JX46 | 0.5125 | 3.07E-02 | 0.1016 | 6.46E-01 |
| Q78PB6 | -0.0211 | 8.94E-01 | -0.2954 | 2.17E-02 |
| G3V8Y8 | 0.1149 | 3.94E-01 | 0.2690 | 3.55E-02 |
| Q4QQU6 | 0.0301 | 7.20E-01 | 0.1701 | 2.47E-02 |
| Q7TPJ0 | 0.4796 | 1.75E-01 | 0.6348 | 7.06E-02 |
| A0A0G2JZ79 | -0.3769 | 3.22E-02 | 0.0492 | 7.37E-01 |
| G3V6Y7 | -0.1410 | 1.09E-01 | -0.1424 | 1.18E-01 |
| P62142 | -0.2949 | 1.23E-01 | -0.3309 | 7.37E-02 |
| A0A0G2JXT9 | -0.1875 | 2.16E-01 | -0.2811 | 5.56E-02 |
| Q62826 | 0.1683 | 5.24E-01 | 0.5234 | 2.99E-02 |
| F1LVI3 | -0.2243 | 2.74E-02 | -0.0129 | 9.09E-01 |
| Q5PQM2 | -0.3760 | 5.80E-02 | -0.2684 | 1.75E-01 |
| F1LRS8 | -0.2306 | 2.96E-02 | 0.0454 | 6.91E-01 |
| D4A6W6 | -0.3149 | 1.28E-01 | -0.3285 | 1.05E-01 |
| D3ZIE4 | 0.5242 | 1.01E-01 | 0.4877 | 1.30E-01 |
| Q498U4 | 0.0946 | 4.18E-01 | 0.2275 | 3.65E-02 |
| Q75WE7 | -0.1167 | 4.63E-01 | 0.3533 | 3.34E-02 |
| A0A0G2JZ09 | -0.2213 | 2.34E-01 | 0.4062 | 5.00E-02 |
| P24090 | 0.0577 | 7.51E-01 | 0.3615 | 2.63E-02 |
| O70185 | -0.3730 | 6.46E-02 | -0.2413 | 1.79E-01 |
| P84586 | -0.3688 | 4.13E-02 | -0.1488 | 4.14E-01 |
| Q4KM73 | -0.4161 | 5.80E-02 | -0.1708 | 2.48E-01 |
| Q5XIH7 | -0.3316 | 1.06E-01 | -0.3154 | 1.24E-01 |
| Q9EPJ0 | 0.2033 | 3.01E-01 | 0.3508 | 5.00E-02 |
| A0A0G2K2Z0 | -0.0790 | 7.55E-01 | 0.5465 | 2.90E-02 |
| Q64548 | -0.3530 | 6.03E-02 | -0.2075 | 2.14E-01 |
| P16036 | -0.3903 | 3.87E-02 | -0.1479 | 4.74E-01 |
| B4F7A5 | -0.2585 | 1.35E-01 | -0.2666 | 1.11E-01 |
| P18666 | -0.2074 | 1.33E-01 | -0.2106 | 1.14E-01 |
| D3ZC89 | -0.0052 | 9.86E-01 | 0.6288 | 2.85E-02 |
| D3Z8L5 | 0.3225 | 3.83E-01 | 0.7503 | 4.01E-02 |
| B2RZB6 | -0.1039 | 4.10E-01 | -0.2268 | 4.12E-02 |
| D3ZSR2 | -0.3959 | 1.11E-01 | -0.3667 | 1.31E-01 |
| A0A0G2JZI2 | 0.2439 | 2.17E-01 | 0.3456 | 7.34E-02 |
| Q4KLL0 | 0.0628 | 6.46E-01 | 0.2537 | 3.06E-02 |
| Q3MID3 | 0.1287 | 2.75E-01 | 0.2082 | 5.93E-02 |
| Q07969 | 0.1919 | 1.35E-01 | 0.2146 | 1.15E-01 |
| Q64598 | -0.4225 | 4.69E-02 | -0.1813 | 4.04E-01 |
| P97584 | -0.3653 | 4.09E-02 | -0.0967 | 5.38E-01 |
| B5DFC8 | 0.1959 | 1.95E-01 | 0.2522 | 7.91E-02 |
| A0A0G2K6R8 | 0.0851 | 3.68E-01 | 0.1750 | 4.72E-02 |
| D4A022 | -0.3291 | 1.14E-01 | -0.3068 | 1.10E-01 |
| Q5M949 | 0.2962 | 1.15E-01 | 0.2836 | 1.08E-01 |
| Q6MG48 | -0.1221 | 3.31E-02 | -0.0155 | 8.01E-01 |
| P04550 | -0.1684 | 1.05E-01 | -0.1460 | 1.57E-01 |
| Q4V7C7 | -0.4214 | 4.54E-02 | -0.1523 | 4.54E-01 |
| B5DEP7 | 0.0916 | 5.36E-01 | 0.2934 | 3.72E-02 |
| A0A0G2QC38 | -0.3867 | 7.00E-02 | -0.2743 | 2.27E-01 |
| D3ZJG8 | 0.2309 | 5.86E-01 | 0.8846 | 3.57E-02 |
| Q8K3E7 | -0.0213 | 8.37E-01 | 0.1960 | 2.96E-02 |
| Q05982 | 0.4653 | 2.26E-01 | 0.7264 | 7.24E-02 |
| Q3MHS2 | -0.2228 | 3.51E-02 | -0.0305 | 7.88E-01 |
| P05943 | 0.0154 | 9.31E-01 | 0.3293 | 2.92E-02 |
| M0R9Q1 | -0.2753 | 5.70E-02 | -0.1405 | 3.36E-01 |
| F1M024 | -0.3385 | 5.34E-02 | 0.1544 | 3.37E-01 |
| Q63083 | -0.0930 | 1.48E-01 | -0.0994 | 1.17E-01 |
| Q920L0 | 0.4358 | 1.73E-01 | 0.5252 | 9.35E-02 |
| D4A589 | 0.3316 | 5.63E-02 | 0.1641 | 3.13E-01 |
| D4AD01 | 0.1422 | 2.92E-01 | 0.2352 | 6.19E-02 |
| Q63678 | 0.1739 | 3.47E-01 | 0.3603 | 5.68E-02 |
| D3ZU85 | -0.3448 | 3.75E-02 | -0.0001 | 9.99E-01 |
| D4A4Z9 | 0.0855 | 5.25E-01 | 0.2496 | 3.99E-02 |
| Q5M860 | 0.2324 | 4.18E-01 | 0.5279 | 4.72E-02 |
| F1LQ29 | -0.1573 | 3.80E-01 | -0.3309 | 5.14E-02 |
| Q6MGB5 | -0.1639 | 5.58E-01 | -0.6023 | 4.00E-02 |
| F1M8B7 | -0.1601 | 2.32E-01 | -0.2226 | 7.89E-02 |
| Q63623 | -0.1763 | 1.47E-01 | -0.1801 | 1.13E-01 |
| P23965 | 0.3687 | 3.77E-02 | 0.0821 | 6.81E-01 |
| P36953 | 0.5472 | 1.45E-01 | 0.5664 | 1.27E-01 |
| M0R5N4 | -0.1559 | 5.63E-02 | -0.0728 | 3.83E-01 |
| Q62736 | -0.1874 | 6.69E-02 | -0.1067 | 3.05E-01 |
| B4F786 | -0.1275 | 4.61E-01 | 0.3120 | 4.37E-02 |
| A0A0G2JVM2 | -0.1679 | 3.52E-02 | 0.0018 | 9.85E-01 |
| P16617 | -0.3115 | 3.77E-02 | -0.0602 | 7.24E-01 |
| P19944 | 0.3442 | 2.95E-01 | 0.5781 | 6.57E-02 |
| P14046 | 0.1674 | 3.93E-01 | 0.3585 | 5.29E-02 |
| F1M9N8 | -0.0549 | 8.35E-01 | -0.5731 | 3.82E-02 |
| P17209 | -0.3605 | 6.54E-02 | -0.2042 | 3.23E-01 |
| P70623 | 0.1431 | 5.35E-01 | 0.4446 | 4.31E-02 |
| P41350 | -0.2113 | 5.74E-02 | -0.0985 | 3.92E-01 |
| P25236 | 0.2656 | 2.85E-01 | 0.4328 | 7.20E-02 |
| G3V6C3 | 0.1115 | 7.55E-01 | 0.6674 | 3.65E-02 |
| Q7TQ77 | 0.0936 | 6.72E-01 | 0.4030 | 3.81E-02 |
| Q499V6 | 0.4762 | 4.54E-02 | 0.0705 | 7.37E-01 |
| P09605 | -0.1794 | 4.69E-01 | -0.4462 | 4.81E-02 |
| P48037 | 0.1983 | 1.28E-01 | 0.1849 | 1.58E-01 |
| Q32KK2 | 0.1645 | 3.99E-01 | -0.3700 | 4.97E-02 |
| P29410 | -0.4684 | 4.43E-02 | -0.0343 | 8.71E-01 |
| D3ZWS6 | -0.1948 | 3.76E-01 | -0.3947 | 5.94E-02 |
| P13221 | 0.4763 | 4.54E-02 | 0.1202 | 6.28E-01 |
| P14668 | 0.1361 | 3.78E-01 | 0.2798 | 5.95E-02 |
| A0A0G2K1A1 | -0.2224 | 4.76E-02 | -0.0644 | 5.80E-01 |
| Q6TA25 | -0.0512 | 8.58E-01 | -0.4782 | 3.51E-02 |
| Q01986 | 0.1613 | 9.51E-02 | 0.1187 | 2.23E-01 |
| Q91V33 | -0.1110 | 4.17E-01 | -0.2492 | 5.61E-02 |
| B2RYV8 | 0.2570 | 3.94E-02 | -0.0204 | 8.88E-01 |
| A0A0G2K9Y9 | -0.3243 | 7.64E-02 | -0.1663 | 2.69E-01 |
| Q9Z1H9 | -0.2140 | 7.21E-02 | 0.1218 | 3.20E-01 |
| D3ZE49 | 0.4530 | 3.31E-02 | 0.2428 | 6.49E-01 |
| Q6P799 | -0.1814 | 1.21E-01 | 0.1605 | 1.68E-01 |
| Q6AY57 | -0.3410 | 5.39E-02 | 0.1202 | 4.85E-01 |
| A0A0G2K8A6 | -0.4115 | 1.24E-01 | -0.3729 | 1.44E-01 |
| D3ZSN4 | -0.0352 | 7.79E-01 | -0.2325 | 3.96E-02 |
| D4A7L4 | -0.1818 | 2.53E-01 | -0.2961 | 7.60E-02 |
| Q63356 | 0.3592 | 2.02E-01 | 0.4550 | 1.05E-01 |
| P42335 | -0.3178 | 7.30E-02 | -0.1866 | 3.04E-01 |
| Q498E0 | 0.1933 | 1.74E-01 | 0.2142 | 1.34E-01 |
| D4A4W9 | -0.4611 | 1.16E-01 | -0.3663 | 2.09E-01 |
| M0R469 | -0.3364 | 1.44E-01 | -0.3167 | 1.53E-01 |
| D4A4L4 | -0.5282 | 8.35E-02 | -0.3133 | 2.66E-01 |
| Q5XIF4 | -0.3109 | 7.90E-02 | -0.1693 | 3.34E-01 |
| P11915 | 0.0021 | 9.90E-01 | -0.2842 | 3.91E-02 |
| M0RD63 | 0.2325 | 8.69E-02 | 0.1414 | 3.02E-01 |
| Q5U2R7 | -0.1245 | 3.90E-01 | -0.2499 | 6.80E-02 |
| Q63570 | 0.1268 | 6.81E-01 | 0.5868 | 4.78E-02 |
| D3ZAF9 | -0.0101 | 9.65E-01 | 0.3941 | 4.12E-02 |
| P31211 | 0.0428 | 8.80E-01 | 0.4940 | 4.09E-02 |
| E9PTI6 | 0.0732 | 7.16E-01 | 0.3605 | 4.51E-02 |
| B1WC56 | 0.3500 | 1.49E-01 | 0.3296 | 1.57E-01 |
| Q499N6 | -0.1690 | 1.37E-01 | -0.1464 | 1.85E-01 |
| Q63627 | 0.1582 | 5.55E-01 | 0.4996 | 5.37E-02 |
| P42123 | 0.4540 | 1.16E-01 | 0.3505 | 2.23E-01 |
| F6Q5K7 | -0.0792 | 7.26E-01 | 0.3713 | 4.37E-02 |
| D4A531 | 0.0726 | 5.21E-01 | 0.2025 | 5.63E-02 |
| Q6AYB3 | -0.0451 | 7.32E-01 | 0.2406 | 4.62E-02 |
| B2GV06 | -0.3878 | 6.36E-02 | -0.1480 | 4.83E-01 |
| Q9JIX3 | -0.0443 | 8.41E-01 | 0.4412 | 4.78E-02 |
| Q5XIB1 | -0.1714 | 9.07E-02 | -0.1058 | 2.87E-01 |
| M0RCY2 | 0.2305 | 4.56E-02 | 0.0184 | 8.93E-01 |
| Q4V8G6 | -0.1289 | 5.83E-01 | -0.3654 | 4.78E-02 |
| P50123 | 0.3647 | 7.17E-02 | -0.1488 | 4.24E-01 |
| B5DFL5 | -0.0584 | 7.58E-01 | -0.3544 | 4.79E-02 |
| D4A7B1 | -0.3575 | 6.91E-02 | -0.1429 | 4.51E-01 |
| P12368 | -0.3843 | 1.65E-01 | -0.3830 | 1.59E-01 |
| D4A4L2 | -0.1889 | 4.73E-02 | 0.0035 | 9.76E-01 |
| E9PSY8 | 0.2030 | 1.25E-01 | 0.1686 | 2.19E-01 |
| Q04940 | 0.7085 | 6.43E-02 | 0.1711 | 5.96E-01 |
| D3ZQ57 | 0.0852 | 3.54E-01 | 0.1479 | 7.89E-02 |
| D3Z9D2 | -0.1788 | 1.49E-01 | -0.1654 | 1.84E-01 |
| F1LW07 | -0.1595 | 2.42E-01 | -0.2097 | 1.13E-01 |
| D3ZTK5 | -0.3297 | 1.73E-01 | -0.3439 | 1.59E-01 |
| A0A0G2K8K0 | 0.1145 | 5.17E-01 | 0.3059 | 6.16E-02 |
| Q80X08 | 0.0673 | 4.98E-01 | 0.1712 | 6.36E-02 |
| Q5XJW2 | -0.1378 | 5.44E-01 | -0.3883 | 5.96E-02 |
| D3Z994 | 0.3993 | 7.12E-02 | 0.1471 | 4.70E-01 |
| D3ZUL1 | -0.1768 | 1.66E-01 | -0.1730 | 1.69E-01 |
| P47853 | 0.1663 | 3.15E-01 | 0.2725 | 9.33E-02 |
| Q5I0E1 | -0.0214 | 9.02E-01 | 0.3380 | 5.11E-02 |
| D4A7Q6 | -0.1462 | 4.60E-01 | -0.3284 | 6.55E-02 |
| Q5XI81 | -0.1039 | 3.69E-01 | -0.1868 | 7.99E-02 |
| M0R9L3 | -0.2219 | 2.50E-01 | -0.2944 | 1.13E-01 |
| M0R7Y5 | -0.3774 | 2.43E-01 | -0.4904 | 1.18E-01 |
| P02091 | 0.6047 | 1.36E-01 | -0.5127 | 2.15E-01 |
| F7FJQ3 | 0.4763 | 7.38E-02 | 0.2108 | 4.54E-01 |
| A0A0G2K6T9 | -0.2457 | 9.02E-02 | -0.1418 | 3.45E-01 |
| Q9WVK7 | -0.2232 | 1.80E-01 | -0.2275 | 1.60E-01 |
| B5DF74 | 0.1725 | 4.25E-01 | 0.3835 | 6.98E-02 |
| D3Z8P5 | 0.2949 | 6.39E-02 | -0.0734 | 6.37E-01 |
| Q6JE36 | 0.5393 | 8.05E-02 | 0.2345 | 4.03E-01 |
| Q9Z0V5 | 0.0294 | 8.83E-01 | 0.3404 | 4.80E-02 |
| Q4V898 | -0.2670 | 1.85E-01 | -0.2802 | 1.57E-01 |
| A0A0G2K3Z9 | -0.0597 | 7.07E-01 | 0.2745 | 5.39E-02 |
| A0A0G2K8N9 | 0.0764 | 5.44E-01 | 0.2108 | 6.32E-02 |
| F1M5Q4 | -0.0452 | 8.45E-01 | 0.3806 | 4.91E-02 |
| B2RYP4 | -0.2593 | 9.71E-02 | -0.1176 | 3.57E-01 |
| D4ADX8 | 0.3860 | 7.28E-02 | 0.1290 | 5.16E-01 |
| Q10728 | -0.1055 | 1.97E-01 | -0.1144 | 1.54E-01 |
| Q5M865 | -0.5220 | 1.48E-01 | -0.4619 | 1.77E-01 |
| F1M110 | -0.1518 | 2.92E-01 | -0.2241 | 1.08E-01 |
| P97839 | -0.1989 | 2.59E-01 | -0.2509 | 1.13E-01 |
| Q6IE52 | 0.3327 | 1.38E-01 | 0.2677 | 2.24E-01 |
| D3ZY96 | -0.4656 | 1.66E-01 | 0.4886 | 1.58E-01 |
| A0A0G2K896 | 0.1199 | 4.65E-01 | 0.2756 | 7.45E-02 |
| Q9Z2S9 | -0.1349 | 4.09E-01 | -0.2592 | 8.05E-02 |
| E9PTX9 | -0.2415 | 9.39E-02 | -0.1202 | 3.81E-01 |
| D3ZZ21 | -0.3373 | 2.04E-01 | -0.3882 | 1.32E-01 |
| P62634 | -0.0436 | 6.36E-01 | -0.1555 | 5.99E-02 |
| P12847 | -0.2358 | 1.73E-01 | -0.2257 | 1.84E-01 |
| E9PSX8 | 0.2688 | 2.44E-01 | 0.3682 | 1.14E-01 |
| P47858 | -0.5001 | 5.80E-02 | 0.0151 | 9.60E-01 |
| Q6MFY6 | -0.3095 | 9.78E-02 | -0.1451 | 3.45E-01 |
| D3ZT71 | -0.3675 | 2.06E-01 | -0.4050 | 1.53E-01 |
| M0R6Z9 | -0.0441 | 7.36E-01 | -0.2241 | 5.68E-02 |
| D3ZPK4 | 0.2814 | 5.87E-02 | -0.0211 | 8.99E-01 |
| F1LMZ8 | -0.2932 | 1.16E-01 | -0.1919 | 2.95E-01 |
| O35165 | 0.2060 | 2.69E-01 | 0.2797 | 1.17E-01 |
| Q9EST6 | 0.1798 | 2.62E-01 | 0.2452 | 1.27E-01 |
| P08289 | 0.2066 | 4.14E-01 | -0.4636 | 8.28E-02 |
| Q68FR9 | 0.1307 | 4.75E-01 | 0.3079 | 7.82E-02 |
| Q498D0 | -0.2578 | 3.29E-01 | -0.4656 | 9.82E-02 |
| P83883 | -0.3052 | 9.14E-02 | -0.1497 | 4.13E-01 |
| D3ZU55 | 0.3240 | 7.64E-02 | 0.0797 | 6.15E-01 |
| O08719 | 0.2897 | 3.83E-01 | 0.5337 | 9.05E-02 |
| O88761 | -0.1105 | 2.36E-01 | -0.1286 | 1.40E-01 |
| B1WC34 | 0.0611 | 6.40E-01 | 0.2259 | 6.51E-02 |
| P07151 | -0.2877 | 1.80E-01 | -0.2906 | 1.88E-01 |
| Q62908 | 0.2901 | 8.52E-02 | 0.1183 | 4.83E-01 |
| P13668 | -0.0417 | 7.69E-01 | 0.2318 | 5.92E-02 |
| P52164 | -0.2931 | 5.77E-02 | -0.0422 | 8.51E-01 |
| Q5RK09 | -0.1883 | 9.59E-02 | 0.0966 | 4.06E-01 |
| D4ADF6 | 0.1911 | 6.61E-02 | 0.0330 | 7.66E-01 |
| Q9QZK5 | 0.3281 | 1.04E-01 | 0.1882 | 3.39E-01 |
| M0RCH6 | 0.1941 | 1.38E-01 | 0.1452 | 2.64E-01 |
| A0A140UHX6 | -0.2466 | 2.35E-01 | -0.2882 | 1.45E-01 |
| P14604 | -0.0633 | 7.72E-01 | -0.3713 | 6.17E-02 |
| Q63042 | -0.0358 | 8.74E-01 | -0.3831 | 5.99E-02 |
| P17431 | 0.8622 | 1.38E-01 | 0.6493 | 2.36E-01 |
| Q5XIA2 | -0.0880 | 1.84E-01 | -0.0836 | 1.96E-01 |
| Q62733 | -0.1342 | 1.76E-01 | 0.1272 | 2.07E-01 |
| D3ZE00 | 0.3932 | 2.39E-01 | 0.4803 | 1.52E-01 |
| D3ZZT3 | -0.0182 | 9.69E-01 | 0.3541 | 4.01E-02 |
| Q8CGU6 | 0.4238 | 2.93E-01 | 0.6239 | 1.13E-01 |
| P38062 | -0.1051 | 4.60E-01 | -0.2283 | 8.64E-02 |
| Q9JHZ4 | -0.1481 | 1.74E-01 | -0.1355 | 2.10E-01 |
| P81828 | -0.0879 | 6.01E-01 | -0.4434 | 8.48E-02 |
| Q9R1E9 | 0.1856 | 3.68E-01 | 0.3474 | 9.81E-02 |
| Q5BJN8 | 0.0029 | 9.86E-01 | 0.2919 | 6.42E-02 |
| P05544 | 0.2015 | 5.33E-01 | 0.5249 | 8.05E-02 |
| B2RZD6 | -0.1613 | 2.92E-01 | 0.2278 | 1.32E-01 |
| F1LNH3 | -0.2987 | 1.35E-01 | -0.2026 | 2.93E-01 |
| Q68FR2 | 0.9956 | 9.78E-02 | 0.4311 | 4.58E-01 |
| Q3KR59 | -0.1672 | 8.47E-02 | -0.0586 | 5.55E-01 |
| G3V8D4 | 0.0594 | 8.70E-01 | 0.5776 | 6.30E-02 |
| Q63528 | 0.0413 | 8.13E-01 | 0.3340 | 7.20E-02 |
| D4A4Z0 | 0.2564 | 1.66E-01 | 0.2512 | 2.07E-01 |
| B2RYF1 | -0.5065 | 1.42E-01 | -0.3693 | 2.75E-01 |
| A0A0G2K012 | 0.2550 | 7.34E-02 | -0.0371 | 8.03E-01 |
| Q9JJW3 | -0.2431 | 7.16E-02 | 0.0447 | 7.71E-01 |
| Q63207 | 0.2992 | 1.89E-01 | 0.2826 | 2.10E-01 |
| Q7TP34 | 0.0777 | 6.18E-01 | 0.2542 | 7.59E-02 |
| Q62622 | 0.2445 | 6.91E-02 | -0.0152 | 9.19E-01 |
| Q66H71 | -0.1164 | 5.68E-01 | -0.3229 | 8.05E-02 |
| A0A096MIX2 | -0.2917 | 1.03E-01 | 0.1375 | 4.51E-01 |
| P27321 | 0.0309 | 6.69E-01 | 0.1110 | 7.24E-02 |
| Q5PQQ8 | 0.2731 | 2.34E-01 | 0.3000 | 1.74E-01 |
| D2XV59 | -0.1688 | 3.76E-01 | -0.2869 | 1.14E-01 |
| Q99N37 | 0.0694 | 5.88E-01 | 0.2046 | 8.19E-02 |
| M0R3L1 | -0.0967 | 3.83E-01 | -0.1701 | 1.13E-01 |
| P22062 | -0.2530 | 7.34E-02 | 0.0202 | 9.01E-01 |
| Q8K1P7 | -0.2124 | 2.60E-01 | -0.2803 | 1.50E-01 |
| D3ZYR1 | -0.2927 | 1.54E-01 | 0.2291 | 2.62E-01 |
| Q63190 | 0.0671 | 6.02E-01 | 0.2009 | 8.40E-02 |
| G3V9L1 | -0.2580 | 1.80E-01 | -0.2261 | 2.19E-01 |
| A0A0G2JZL4 | -0.1396 | 3.54E-01 | -0.2160 | 1.29E-01 |
| F1M3H8 | -0.1607 | 3.62E-01 | 0.2598 | 1.27E-01 |
| P29457 | 0.2403 | 2.82E-01 | 0.3038 | 1.59E-01 |
| D4AEJ0 | -0.0203 | 9.49E-01 | 0.4685 | 7.34E-02 |
| D4A471 | -0.1978 | 7.90E-02 | 0.0031 | 9.82E-01 |
| G3V9G5 | 0.1887 | 1.35E-01 | 0.1157 | 3.69E-01 |
| O55043 | 0.1693 | 5.77E-01 | -0.5118 | 9.46E-02 |
| P06765 | -0.4390 | 1.83E-01 | -0.3839 | 2.49E-01 |
| D3ZPL1 | 0.0405 | 8.53E-01 | 0.3260 | 7.29E-02 |
| D3ZAS9 | 0.1008 | 6.10E-01 | 0.3092 | 9.03E-02 |
| Q58FK9 | 0.1870 | 5.15E-01 | -0.4174 | 1.01E-01 |
| Q5U2U2 | -0.2617 | 9.30E-02 | -0.0733 | 6.58E-01 |
| D4A3V5 | -0.1415 | 4.72E-01 | -0.3019 | 1.05E-01 |
| D3ZUI9 | -0.1779 | 1.21E-01 | -0.0888 | 4.43E-01 |
| D4A9N5 | -0.2713 | 1.50E-01 | 0.2016 | 2.97E-01 |
| P01041 | -0.1083 | 5.77E-01 | -0.3118 | 9.79E-02 |
| Q32KJ6 | 0.2574 | 5.77E-01 | 0.7091 | 9.63E-02 |
| D3ZET9 | 0.1265 | 4.64E-01 | 0.2565 | 1.13E-01 |
| Q5U300 | -0.0660 | 8.79E-01 | 0.6921 | 7.94E-02 |
| A1A5P0 | -0.3252 | 1.04E-01 | -0.0821 | 6.46E-01 |
| Q6AYQ8 | -0.1710 | 4.77E-01 | -0.3511 | 1.11E-01 |
| P84903 | -0.2970 | 8.95E-02 | -0.0479 | 7.98E-01 |
| Q6PCU2 | -0.0512 | 7.88E-01 | 0.2927 | 8.17E-02 |
| P08592 | 0.2178 | 1.19E-01 | -0.1066 | 4.77E-01 |
| A0A0G2K6E8 | 0.1199 | 5.04E-01 | 0.2648 | 1.10E-01 |
| P80067 | -0.1930 | 2.12E-01 | 0.1815 | 2.34E-01 |
| A9UMW3 | -0.3752 | 4.06E-01 | -0.6327 | 1.30E-01 |
| Q63118 | -0.2717 | 9.16E-02 | -0.0375 | 8.27E-01 |
| Q9QXU8 | -0.0296 | 8.35E-01 | 0.2232 | 8.43E-02 |
| Q5U1Z8 | -0.3544 | 4.13E-01 | -0.6284 | 1.32E-01 |
| Q68FT1 | -0.2866 | 8.67E-02 | 0.0096 | 9.61E-01 |
| Q8K3X8 | -0.3194 | 1.21E-01 | -0.1438 | 4.94E-01 |
| P80432 | -0.2017 | 1.74E-01 | 0.1640 | 3.07E-01 |
| D4A321 | -0.1770 | 1.80E-01 | -0.1389 | 2.91E-01 |
| Q5XIU5 | -0.1299 | 5.77E-01 | -0.3393 | 1.03E-01 |
| A0A0G2K6H7 | -0.1253 | 5.88E-01 | -0.3504 | 1.02E-01 |
| P06302 | 0.0955 | 5.99E-01 | 0.2714 | 1.02E-01 |
| D3ZGS7 | 0.3444 | 2.22E-01 | 0.3328 | 2.36E-01 |
| G3V8L1 | 0.1788 | 3.32E-01 | 0.2468 | 1.64E-01 |
| P11507 | -0.1631 | 8.95E-02 | 0.0180 | 8.73E-01 |
| A5HKJ3 | 0.4241 | 1.39E-01 | -0.2545 | 3.92E-01 |
| B5DF60 | 0.2386 | 1.74E-01 | 0.1631 | 3.30E-01 |
| D4A7Z2 | 0.1741 | 2.80E-01 | 0.2177 | 1.98E-01 |
| Q9JHW0 | 0.1463 | 2.70E-01 | 0.1636 | 2.07E-01 |
| Q9Z1E1 | -0.1705 | 2.52E-01 | -0.1760 | 2.23E-01 |
| Q712U5 | -0.0234 | 7.83E-01 | -0.1227 | 9.03E-02 |
| B0BN18 | -0.0787 | 1.85E-01 | 0.0595 | 3.13E-01 |
| G3V7Q7 | 0.0108 | 9.61E-01 | -0.3897 | 9.84E-02 |
| D3ZF45 | 0.0883 | 6.55E-01 | 0.2860 | 1.02E-01 |
| Q9Z0J5 | 0.3679 | 1.25E-01 | 0.1465 | 5.41E-01 |
| Q62806 | 0.1181 | 6.28E-01 | -0.3784 | 1.08E-01 |
| Q9QZA2 | 0.1570 | 3.12E-01 | -0.1979 | 1.85E-01 |
| Q5XI68 | -0.2637 | 1.04E-01 | -0.0550 | 7.55E-01 |
| Q62947 | -0.2006 | 1.15E-01 | -0.0598 | 6.55E-01 |
| Q9JI51 | 0.2887 | 1.24E-01 | -0.0810 | 6.38E-01 |
| Q925G1 | 0.3265 | 2.77E-01 | 0.3567 | 2.17E-01 |
| A0A0G2K8W9 | -0.1398 | 1.85E-01 | -0.1018 | 3.41E-01 |
| D3ZUU5 | 0.4488 | 1.41E-01 | 0.2091 | 4.90E-01 |
| Q5XIF6 | -0.1485 | 4.17E-01 | 0.2618 | 1.55E-01 |
| B0BNE6 | -0.3727 | 1.37E-01 | -0.1694 | 5.12E-01 |
| Q5XHY7 | 0.0934 | 6.81E-01 | 0.3243 | 1.08E-01 |
| Q6PEC4 | -0.0884 | 5.02E-01 | -0.1869 | 1.33E-01 |
| Q5I0H3 | -0.2268 | 1.73E-01 | -0.1463 | 3.84E-01 |
| A0A0G2K5M1 | -0.2600 | 1.21E-01 | -0.0705 | 6.86E-01 |
| P25409 | 0.4747 | 1.14E-01 | -0.0413 | 8.90E-01 |
| Q5M8C3 | 0.1945 | 2.93E-01 | 0.2201 | 2.17E-01 |
| Q5U317 | 0.0766 | 4.73E-01 | 0.1463 | 1.42E-01 |
| Q62910 | 0.0833 | 5.03E-01 | 0.1695 | 1.35E-01 |
| Q5XIJ6 | 0.0653 | 8.41E-01 | 0.4783 | 1.05E-01 |
| P61980 | -0.0323 | 7.67E-01 | 0.1583 | 1.05E-01 |
| Q3ZAV8 | 0.0926 | 5.10E-01 | 0.1903 | 1.34E-01 |
| Q5FVC2 | 0.2638 | 2.54E-01 | 0.2650 | 2.38E-01 |
| O88794 | -0.2877 | 1.57E-01 | -0.1564 | 4.55E-01 |
| Q6YDN7 | 0.1376 | 4.63E-01 | 0.2490 | 1.46E-01 |
| Q5HZA4 | -0.5477 | 1.65E-01 | -0.3060 | 4.16E-01 |
| D3ZG88 | 0.1534 | 1.27E-01 | 0.0472 | 6.46E-01 |
| B0BN78 | -0.2182 | 1.95E-01 | -0.1614 | 3.45E-01 |
| D3ZZZ9 | -0.0594 | 6.17E-01 | 0.1609 | 1.18E-01 |
| B2GV94 | 0.4071 | 1.77E-01 | 0.2823 | 3.82E-01 |
| E9PSN4 | 0.0514 | 7.69E-01 | 0.2746 | 1.14E-01 |
| A0A0G2K7P6 | -0.3523 | 2.75E-01 | -0.3769 | 2.27E-01 |
| P40112 | 0.2276 | 1.14E-01 | 0.0167 | 9.11E-01 |
| D3ZD19 | 0.1509 | 4.29E-01 | 0.2510 | 1.58E-01 |
| D4AEL2 | -0.2894 | 2.63E-01 | -0.3190 | 2.36E-01 |
| A0A0G2JTK4 | -0.1345 | 4.28E-01 | -0.2279 | 1.62E-01 |
| Q7TP65 | 0.3499 | 1.87E-01 | 0.2217 | 3.63E-01 |
| D4A883 | 0.2594 | 1.76E-01 | 0.1618 | 4.05E-01 |
| A0A0G2K046 | -0.1200 | 3.40E-01 | -0.1549 | 2.01E-01 |
| D3ZYM5 | 0.2518 | 1.42E-01 | -0.1101 | 5.42E-01 |
| D3ZUP5 | 0.0131 | 8.96E-01 | -0.1389 | 1.02E-01 |
| Q7TP99 | -0.2834 | 1.95E-01 | -0.1838 | 3.49E-01 |
| D4A3E2 | 0.2247 | 3.92E-01 | -0.3300 | 1.76E-01 |
| D3ZM69 | 0.0788 | 6.15E-01 | 0.2138 | 1.24E-01 |
| M0RBX6 | -0.3417 | 3.28E-01 | -0.4156 | 2.10E-01 |
| Q8K1Q0 | 0.1311 | 2.08E-01 | 0.0954 | 3.37E-01 |
| Q5XID1 | -0.0591 | 7.14E-01 | -0.2277 | 1.14E-01 |
| B5DFD8 | 0.1296 | 2.79E-01 | 0.1332 | 2.49E-01 |
| D3ZTN1 | -0.1065 | 5.76E-01 | 0.2967 | 1.38E-01 |
| A1L1K8 | 0.0060 | 9.56E-01 | -0.1443 | 1.04E-01 |
| Q9JK25 | -0.0748 | 2.50E-01 | -0.0682 | 2.87E-01 |
| Q64268 | -0.1841 | 3.63E-01 | -0.2451 | 1.88E-01 |
| Q4QQW3 | -0.1483 | 1.80E-01 | -0.0940 | 4.11E-01 |
| M0R907 | -0.2413 | 4.70E-01 | -0.4449 | 1.58E-01 |
| Q9JJ19 | -0.0942 | 3.12E-01 | 0.1065 | 2.32E-01 |
| Q08415 | 0.3275 | 1.23E-01 | -0.0506 | 8.21E-01 |
| P80254 | 0.0324 | 8.74E-01 | -0.2717 | 1.07E-01 |
| A0A0G2K2J9 | -0.1906 | 1.27E-01 | -0.0334 | 7.93E-01 |
| Q9R1T1 | -0.3713 | 2.44E-01 | -0.3358 | 2.99E-01 |
| Q3SWT0 | 0.1613 | 1.98E-01 | 0.1097 | 3.80E-01 |
| Q6TQE1 | 0.2335 | 2.33E-01 | 0.1949 | 3.17E-01 |
| D3ZH28 | -0.0007 | 9.94E-01 | -0.1612 | 1.08E-01 |
| Q66HA4 | -0.0617 | 6.58E-01 | 0.1903 | 1.28E-01 |
| P16086 | 0.1444 | 2.43E-01 | 0.1199 | 3.09E-01 |
| E9PT23 | 0.1405 | 5.32E-01 | 0.2994 | 1.50E-01 |
| B1H245 | -0.1247 | 2.90E-01 | -0.1200 | 2.55E-01 |
| A7VJC2 | -0.1831 | 1.92E-01 | -0.1153 | 4.07E-01 |
| Q63358 | -0.0188 | 9.30E-01 | 0.3133 | 1.20E-01 |
| O55035 | -0.2194 | 2.62E-01 | 0.2021 | 2.89E-01 |
| P50408 | 0.0513 | 8.54E-01 | 0.3810 | 1.14E-01 |
| P0C5H9 | -0.1108 | 3.68E-01 | -0.1488 | 2.08E-01 |
| Q91Y78 | 0.1987 | 3.15E-01 | 0.2126 | 2.36E-01 |
| B2GV38 | -0.0895 | 5.79E-01 | -0.2192 | 1.43E-01 |
| P18421 | 0.1594 | 2.69E-01 | 0.1524 | 2.85E-01 |
| F1LT35 | -0.1377 | 2.43E-01 | 0.1121 | 3.19E-01 |
| P70615 | -0.1208 | 3.80E-01 | 0.1725 | 2.04E-01 |
| P63004 | 0.2498 | 1.59E-01 | 0.1061 | 5.53E-01 |
| Q62745 | 0.2352 | 6.53E-01 | 0.7164 | 1.36E-01 |
| P18292 | -0.0354 | 8.96E-01 | 0.3776 | 1.15E-01 |
| D4ADB4 | 0.2418 | 5.29E-01 | 0.5074 | 1.55E-01 |
| D3ZUI1 | -0.1499 | 5.08E-01 | -0.2918 | 1.60E-01 |
| D3ZFU9 | -0.2044 | 1.25E-01 | 0.0030 | 9.85E-01 |
| A0A0G2JZ48 | 0.0128 | 8.23E-01 | 0.0750 | 1.17E-01 |
| Q8K4K7 | 0.1600 | 6.36E-01 | -0.4684 | 1.39E-01 |
| Q9QXQ0 | -0.2461 | 1.27E-01 | 0.0049 | 9.79E-01 |
| Q6MG08 | 0.0319 | 8.93E-01 | 0.3166 | 1.15E-01 |
| D3ZUM4 | -0.1774 | 1.70E-01 | -0.0817 | 5.22E-01 |
| F1LNI5 | 0.0876 | 6.94E-01 | 0.2999 | 1.32E-01 |
| Q9Z1Y3 | 0.0124 | 9.39E-01 | -0.2068 | 1.16E-01 |
| Q9ESS6 | -0.1803 | 1.57E-01 | 0.0663 | 6.08E-01 |
| Q6P7C7 | 0.2679 | 4.17E-01 | 0.3864 | 1.94E-01 |
| P05545 | 0.2030 | 3.91E-01 | 0.2865 | 2.09E-01 |
| Q62835 | 0.0546 | 6.39E-01 | 0.1539 | 1.41E-01 |
| G3V6B8 | -0.1013 | 4.15E-01 | -0.1437 | 1.98E-01 |
| F1LN75 | -0.0160 | 9.19E-01 | 0.2053 | 1.18E-01 |
| D3ZRE7 | -0.1069 | 3.67E-01 | 0.1454 | 2.25E-01 |
| Q6PCT3 | 0.0869 | 4.92E-01 | 0.1609 | 1.74E-01 |
| Q3MHU5 | -0.0205 | 9.33E-01 | 0.3072 | 1.19E-01 |
| Q505I4 | -0.0967 | 5.83E-01 | -0.2302 | 1.55E-01 |
| G3V7U9 | -0.0450 | 6.82E-01 | -0.1457 | 1.39E-01 |
| Q5PQQ2 | -0.1281 | 2.84E-01 | 0.1214 | 2.96E-01 |
| Q5PQK2 | -0.0179 | 8.79E-01 | 0.1550 | 1.23E-01 |
| F1M4M5 | 0.1441 | 7.24E-01 | 0.5494 | 1.39E-01 |
| Q9EPX0 | 0.0934 | 5.42E-01 | -0.2093 | 1.69E-01 |
| F1LTJ5 | 0.0620 | 5.26E-01 | 0.1219 | 1.69E-01 |
| A0A0G2JVW3 | -0.4205 | 4.10E-01 | -0.6093 | 2.10E-01 |
| P24268 | -0.2171 | 5.80E-01 | -0.4971 | 1.59E-01 |
| D3ZUD3 | -0.6877 | 1.52E-01 | -0.0982 | 8.30E-01 |
| A2RRU1 | 0.2083 | 7.89E-01 | 1.0472 | 1.37E-01 |
| P60192 | 0.1287 | 6.04E-01 | 0.3157 | 1.55E-01 |
| Q5M823 | -0.3178 | 2.52E-01 | -0.3186 | 3.29E-01 |
| P04639 | 0.2370 | 4.17E-01 | 0.3492 | 2.10E-01 |
| Q68FS3 | -0.0512 | 7.76E-01 | -0.4158 | 1.62E-01 |
| Q8VHK7 | 0.1355 | 2.52E-01 | 0.1073 | 3.57E-01 |
| Q63862 | -0.2085 | 1.43E-01 | -0.0254 | 8.70E-01 |
| Q08326 | 0.1537 | 5.05E-01 | -0.2863 | 1.82E-01 |
| D3ZN69 | 0.1110 | 5.15E-01 | 0.2144 | 1.79E-01 |
| Q6MG75 | -0.1797 | 3.68E-01 | -0.1847 | 2.29E-01 |
| F1LWN5 | -0.1982 | 1.87E-01 | -0.1067 | 5.08E-01 |
| C0KUC6 | -0.2144 | 2.45E-01 | -0.1662 | 3.69E-01 |
| D3ZCV0 | -0.1925 | 1.44E-01 | -0.0287 | 8.45E-01 |
| Q6AYD3 | 0.1922 | 2.54E-01 | 0.1688 | 3.42E-01 |
| Q6AYU3 | -0.0474 | 8.63E-01 | -0.3570 | 1.32E-01 |
| F1M2S2 | 0.2834 | 3.61E-01 | 0.3353 | 2.50E-01 |
| Q63644 | 0.1889 | 4.45E-01 | 0.3090 | 2.06E-01 |
| P62907 | -0.3832 | 4.17E-01 | -0.5457 | 2.20E-01 |
| A0A0G2K089 | -0.1399 | 4.41E-01 | -0.2203 | 2.10E-01 |
| D3ZFY8 | -0.1813 | 1.72E-01 | -0.0671 | 6.39E-01 |
| Q6AXS3 | -0.1130 | 3.57E-01 | -0.1266 | 2.64E-01 |
| A0A0G2K419 | -0.1499 | 1.56E-01 | -0.0241 | 8.27E-01 |
| Q00657 | -0.1874 | 1.70E-01 | -0.0558 | 6.91E-01 |
| A0A0G2K2F5 | -0.3886 | 2.86E-01 | 0.3339 | 3.35E-01 |
| A0A0G2K8R3 | 0.0411 | 6.65E-01 | 0.1197 | 1.59E-01 |
| Q505J8 | -0.2392 | 1.91E-01 | 0.1085 | 5.60E-01 |
| F1LZC5 | -0.3022 | 1.74E-01 | -0.0700 | 7.33E-01 |
| D4A1B2 | -0.0585 | 6.54E-01 | -0.1796 | 1.69E-01 |
| P30904 | 0.2614 | 3.25E-01 | -0.2848 | 2.99E-01 |
| Q9JIL3 | -0.3059 | 1.99E-01 | -0.1580 | 5.25E-01 |
| D3ZKN0 | 0.1070 | 4.77E-01 | -0.1784 | 2.11E-01 |
| A0A0G2K1V7 | -0.2562 | 1.77E-01 | -0.0653 | 7.27E-01 |
| Q1JU68 | 0.0439 | 7.00E-01 | 0.1457 | 1.60E-01 |
| Q5U313 | -0.0544 | 8.52E-01 | 0.3535 | 1.45E-01 |
| A0A0G2JTD1 | 0.1916 | 3.03E-01 | 0.1703 | 3.21E-01 |
| G3V7T6 | 0.0460 | 7.02E-01 | 0.1540 | 1.61E-01 |
| G3V8K8 | -0.1067 | 5.72E-01 | 0.2483 | 1.88E-01 |
| B0BN72 | 0.0240 | 7.55E-01 | 0.0991 | 1.57E-01 |
| Q9Z2J5 | 0.0772 | 6.51E-01 | 0.2121 | 1.72E-01 |
| B2GUZ5 | -0.2721 | 1.74E-01 | -0.0468 | 8.10E-01 |
| Q66HS7 | -0.0588 | 5.73E-01 | -0.1258 | 1.88E-01 |
| D3ZL88 | -0.0509 | 8.01E-01 | 0.2469 | 1.53E-01 |
| Q3T1K5 | -0.4287 | 1.78E-01 | -0.1280 | 7.01E-01 |
| D4A3T3 | -0.1107 | 1.80E-01 | -0.0372 | 6.72E-01 |
| Q6DGF4 | -0.0962 | 5.90E-01 | -0.2131 | 1.84E-01 |
| Q642B4 | -0.2031 | 2.66E-01 | -0.1642 | 3.83E-01 |
| A0A0G2K8M7 | -0.2496 | 2.93E-01 | -0.2151 | 3.56E-01 |
| A0A0G2K0C9 | 0.1855 | 7.48E-01 | 0.7036 | 1.59E-01 |
| Q99J86 | 0.1049 | 3.63E-01 | 0.1223 | 2.86E-01 |
| Q9JMB5 | 0.2394 | 2.72E-01 | 0.1864 | 3.89E-01 |
| Q63610 | -0.0126 | 9.31E-01 | 0.1753 | 1.47E-01 |
| Q66HL2 | -0.0722 | 2.34E-01 | -0.0418 | 4.77E-01 |
| D3ZPJ9 | -0.0475 | 8.18E-01 | 0.2705 | 1.61E-01 |
| B1H2A6 | -0.2453 | 1.96E-01 | -0.0986 | 6.09E-01 |
| P15650 | 0.1965 | 2.75E-01 | 0.1545 | 3.86E-01 |
| A0A0G2K781 | 0.2741 | 3.48E-01 | 0.2939 | 2.97E-01 |
| A0A0G2JYA4 | -0.1937 | 1.76E-01 | 0.0456 | 7.60E-01 |
| Q5BJP2 | -0.1174 | 1.66E-01 | 0.0001 | 9.99E-01 |
| O35783 | 0.0165 | 8.74E-01 | 0.1275 | 1.52E-01 |
| D4A986 | -0.0279 | 8.50E-01 | -0.1868 | 1.58E-01 |
| Q8K4S7 | 0.2385 | 1.97E-01 | 0.2285 | 5.38E-01 |
| B1WBX6 | -0.0298 | 8.21E-01 | 0.1584 | 1.59E-01 |
| Q91Y81 | -0.2420 | 3.17E-01 | -0.2233 | 3.38E-01 |
| A0A0G2K3H5 | 0.0656 | 7.41E-01 | 0.2417 | 1.69E-01 |
| Q6PCT5 | 0.1986 | 1.74E-01 | 0.0441 | 7.93E-01 |
| M0RA26 | -0.4040 | 1.80E-01 | -0.0831 | 7.94E-01 |
| Q62785 | -0.0658 | 5.55E-01 | -0.1272 | 2.07E-01 |
| Q7TP47 | -0.2904 | 2.22E-01 | -0.1439 | 5.46E-01 |
| P02600 | -0.1490 | 4.46E-01 | -0.2069 | 2.49E-01 |
| D3ZBJ0 | 0.0581 | 7.54E-01 | -0.2185 | 1.71E-01 |
| P97710 | 0.1750 | 3.74E-01 | 0.1837 | 3.06E-01 |
| Q8JZQ0 | 0.0498 | 7.69E-01 | 0.2190 | 1.82E-01 |
| Q9Z2X5 | 0.0245 | 8.76E-01 | 0.1894 | 1.63E-01 |
| P61959 | -0.1174 | 2.52E-01 | -0.0744 | 4.79E-01 |
| Q4QQV1 | -0.2244 | 2.88E-01 | -0.1765 | 4.07E-01 |
| M0RD14 | -0.2072 | 2.60E-01 | -0.1168 | 4.79E-01 |
| D3ZYL0 | -0.3080 | 2.43E-01 | 0.1708 | 5.18E-01 |
| Q6P686 | 0.0636 | 4.99E-01 | 0.1077 | 2.44E-01 |
| P07340 | -0.2248 | 1.77E-01 | -0.0152 | 9.36E-01 |
| D3ZGN7 | -0.0614 | 8.35E-01 | -0.3433 | 1.73E-01 |
| A0A0G2K350 | 0.0272 | 9.02E-01 | 0.2641 | 1.72E-01 |
| Q1AAU6 | 0.4370 | 2.06E-01 | 0.0926 | 7.81E-01 |
| D3ZT94 | -0.1429 | 4.65E-01 | 0.2365 | 2.64E-01 |
| D4ADT3 | 0.0641 | 6.78E-01 | -0.1837 | 2.03E-01 |
| A0A0G2K712 | 0.0706 | 7.48E-01 | 0.2690 | 1.95E-01 |
| P61972 | 0.3462 | 2.33E-01 | -0.1712 | 5.87E-01 |
| G3V6G1 | 0.1181 | 5.36E-01 | 0.2075 | 2.40E-01 |
| P55053 | -0.2609 | 2.28E-01 | -0.1207 | 6.08E-01 |
| D3Z853 | 0.1833 | 3.82E-01 | -0.1991 | 3.30E-01 |
| P15337 | 0.1720 | 2.06E-01 | 0.0434 | 7.58E-01 |
| Q62952 | 0.0413 | 8.41E-01 | -0.2395 | 1.87E-01 |
| D3ZKG6 | 0.2778 | 4.41E-01 | 0.3611 | 2.91E-01 |
| F1LQ48 | -0.0043 | 9.86E-01 | 0.2953 | 1.75E-01 |
| P04762 | -0.2546 | 1.91E-01 | 0.0109 | 9.61E-01 |
| B0BN56 | 0.2893 | 1.91E-01 | 0.0030 | 9.92E-01 |
| P28480 | -0.2752 | 4.12E-01 | 0.3256 | 3.08E-01 |
| Q62651 | 0.1458 | 3.50E-01 | 0.1327 | 3.74E-01 |
| Q5PQJ5 | -0.2255 | 2.37E-01 | -0.1407 | 5.78E-01 |
| P23347 | -0.4147 | 2.23E-01 | -0.1171 | 7.27E-01 |
| P06685 | -0.1966 | 1.95E-01 | -0.0064 | 9.70E-01 |
| D3ZYT2 | -0.1378 | 6.81E-01 | -0.3769 | 2.10E-01 |
| A0A0G2JX72 | -0.2036 | 2.03E-01 | -0.0377 | 8.27E-01 |
| F1M7E5 | -0.1921 | 2.08E-01 | -0.0416 | 7.96E-01 |
| D4A9Q3 | -0.1595 | 4.67E-01 | -0.2288 | 2.85E-01 |
| F1LW69 | 0.1947 | 5.22E-01 | 0.3187 | 2.57E-01 |
| Q9Z270 | -0.1522 | 3.18E-01 | -0.1188 | 4.24E-01 |
| Q5FVK6 | 0.0379 | 7.79E-01 | 0.1516 | 1.97E-01 |
| P62902 | -0.1264 | 5.70E-01 | -0.2349 | 2.45E-01 |
| P83732 | 0.1569 | 3.14E-01 | -0.1201 | 4.41E-01 |
| P63324 | -0.1743 | 2.06E-01 | -0.0241 | 8.71E-01 |
| P36202 | -0.0309 | 7.99E-01 | -0.1376 | 1.97E-01 |
| D3ZZM3 | -0.1258 | 6.96E-01 | -0.3709 | 2.17E-01 |
| A0A0G2JW03 | -0.0767 | 8.73E-01 | 0.6185 | 2.06E-01 |
| Q6P734 | 0.1319 | 4.86E-01 | 0.1951 | 2.86E-01 |
| Q5M951 | 0.0915 | 4.94E-01 | 0.1373 | 2.82E-01 |
| Q8R5M4 | -0.0104 | 9.42E-01 | -0.1565 | 1.88E-01 |
| E9PTR4 | 0.0385 | 5.77E-01 | 0.0690 | 2.45E-01 |
| Q5FVF9 | -0.3047 | 2.51E-01 | 0.1204 | 6.37E-01 |
| Q5XI28 | -0.0651 | 7.54E-01 | -0.2194 | 2.04E-01 |
| P49134 | 0.0549 | 6.30E-01 | 0.1251 | 2.35E-01 |
| P26453 | 0.0559 | 5.33E-01 | 0.0940 | 2.68E-01 |
| Q5PQT2 | -0.0289 | 9.07E-01 | -0.2804 | 1.95E-01 |
| Q6P7A2 | -0.1409 | 4.17E-01 | -0.1599 | 3.37E-01 |
| Q9QZ86 | -0.2416 | 3.56E-01 | -0.2233 | 4.07E-01 |
| P63055 | -0.4321 | 3.77E-01 | 0.3998 | 3.81E-01 |
| Q08420 | -0.1281 | 4.18E-01 | -0.1448 | 3.45E-01 |
| D3ZAH3 | -0.0449 | 8.74E-01 | 0.3107 | 2.08E-01 |
| D3ZTE0 | -0.2927 | 2.27E-01 | 0.0141 | 9.54E-01 |
| A0A0G2K0D3 | -0.1028 | 4.13E-01 | -0.1121 | 3.52E-01 |
| Q9JHL4 | -0.0982 | 2.71E-01 | 0.0530 | 5.75E-01 |
| B4F7F3 | -0.0028 | 9.93E-01 | -0.3965 | 2.07E-01 |
| Q8CF97 | 0.1046 | 4.35E-01 | 0.1211 | 3.45E-01 |
| F1M1E4 | 0.1176 | 6.88E-01 | 0.3151 | 2.41E-01 |
| Q9JMC1 | -0.2113 | 2.59E-01 | -0.0776 | 6.81E-01 |
| Q4V8B0 | -0.2040 | 2.25E-01 | 0.0117 | 9.49E-01 |
| D4A900 | 0.1965 | 3.67E-01 | 0.1759 | 4.18E-01 |
| D4ABS5 | 0.2166 | 4.84E-01 | -0.2859 | 3.16E-01 |
| D4A0U3 | -0.1596 | 2.48E-01 | -0.0471 | 7.37E-01 |
| P97541 | -0.1233 | 5.86E-01 | -0.2317 | 2.69E-01 |
| G3V6A2 | -0.0392 | 6.93E-01 | -0.1044 | 2.38E-01 |
| Q9ESV1 | 0.1361 | 2.33E-01 | 0.0221 | 8.56E-01 |
| F1LPJ1 | -0.1226 | 5.55E-01 | -0.2060 | 2.85E-01 |
| Q7TP52 | 0.2927 | 2.27E-01 | -0.0165 | 9.51E-01 |
| Q9WUC8 | 0.0709 | 7.41E-01 | 0.2260 | 2.30E-01 |
| D3ZCP9 | 0.2152 | 2.50E-01 | 0.0687 | 7.30E-01 |
| M0R9T2 | -0.0433 | 7.84E-01 | 0.1637 | 2.22E-01 |
| A0A0G2JVQ1 | 0.0092 | 9.61E-01 | -0.1972 | 2.09E-01 |
| D4AB03 | -0.0667 | 7.54E-01 | 0.2208 | 2.32E-01 |
| D4A7G9 | -0.1234 | 3.59E-01 | -0.0976 | 4.55E-01 |
| D3ZWL9 | -0.1415 | 2.36E-01 | 0.0043 | 9.74E-01 |
| P13832 | -0.1356 | 5.24E-01 | 0.1923 | 3.07E-01 |
| A1XIQ3 | 0.4267 | 3.58E-01 | 0.3267 | 4.62E-01 |
| F1LP26 | 0.0704 | 4.03E-01 | -0.0714 | 3.99E-01 |
| A0A0G2K098 | 0.0550 | 8.21E-01 | 0.2381 | 2.29E-01 |
| P11762 | -0.0623 | 5.87E-01 | -0.1169 | 2.85E-01 |
| F1M293 | -0.2855 | 3.58E-01 | -0.2271 | 4.64E-01 |
| G3V9R2 | -0.0582 | 7.69E-01 | 0.2144 | 2.37E-01 |
| D4AA63 | -0.1343 | 2.86E-01 | -0.0622 | 6.26E-01 |
| Q5XID6 | -0.1299 | 2.46E-01 | -0.0243 | 8.40E-01 |
| Q62871 | 0.1922 | 3.39E-01 | 0.1263 | 5.07E-01 |
| P13852 | 0.0607 | 7.50E-01 | -0.1987 | 2.44E-01 |
| P12001 | -0.1931 | 3.57E-01 | -0.1483 | 4.77E-01 |
| B5DFK6 | 0.2829 | 3.57E-01 | 0.2147 | 4.79E-01 |
| P68035 | -0.0647 | 7.72E-01 | 0.2305 | 2.39E-01 |
| F7FEM5 | 0.0408 | 8.84E-01 | -0.2959 | 2.33E-01 |
| Q62894 | 0.0818 | 3.56E-01 | 0.0629 | 4.83E-01 |
| Q5XI34 | -0.1482 | 7.96E-01 | 0.7232 | 2.52E-01 |
| Q4QQV8 | 0.0255 | 7.84E-01 | -0.0967 | 2.42E-01 |
| P63029 | 0.0615 | 8.32E-01 | 0.2931 | 2.36E-01 |
| A0A0G2K904 | -0.0963 | 5.39E-01 | -0.1463 | 3.21E-01 |
| A0A0G2K1P8 | -0.0488 | 8.43E-01 | -0.2556 | 2.36E-01 |
| Q6AXU4 | -0.0105 | 9.54E-01 | 0.1890 | 2.29E-01 |
| Q01750 | 0.1515 | 6.74E-01 | 0.3572 | 2.71E-01 |
| D3ZVW3 | -0.0799 | 3.66E-01 | -0.0617 | 4.82E-01 |
| P97546 | 0.0613 | 7.84E-01 | 0.2374 | 2.55E-01 |
| A0A0G2K916 | -0.1453 | 5.53E-01 | 0.2276 | 3.20E-01 |
| P10252 | -0.3613 | 2.72E-01 | 0.0737 | 8.20E-01 |
| D4ABH1 | 0.0333 | 7.98E-01 | 0.1380 | 2.49E-01 |
| D4A9L2 | 0.1188 | 2.82E-01 | 0.0435 | 7.06E-01 |
| P63045 | -0.2176 | 6.17E-01 | -0.4125 | 2.99E-01 |
| Q8R490 | -0.1001 | 2.84E-01 | -0.0341 | 7.20E-01 |
| D3ZYS7 | -0.1524 | 2.82E-01 | 0.0528 | 7.22E-01 |
| Q32KJ5 | 0.1422 | 5.60E-01 | 0.2253 | 3.23E-01 |
| Q5XIE0 | -0.0253 | 8.94E-01 | 0.1953 | 2.40E-01 |
| A0A0G2K5Z4 | 0.2084 | 2.92E-01 | -0.0815 | 7.01E-01 |
| F1LW22 | 0.0984 | 8.36E-01 | -0.4786 | 2.52E-01 |
| Q5XIH6 | 0.2101 | 3.40E-01 | 0.1055 | 5.97E-01 |
| P07154 | 0.0323 | 7.75E-01 | 0.1127 | 2.62E-01 |
| Q8K4V4 | -0.0488 | 6.93E-01 | -0.1183 | 2.84E-01 |
| O89046 | 0.1496 | 6.31E-01 | 0.2914 | 3.02E-01 |
| P10818 | 0.0712 | 7.69E-01 | -0.2381 | 2.64E-01 |
| O55156 | 0.0139 | 9.65E-01 | 0.3033 | 2.42E-01 |
| B2RYI2 | 0.1973 | 3.15E-01 | -0.0729 | 6.88E-01 |
| Q5XIS7 | 0.1327 | 3.95E-01 | 0.1082 | 4.81E-01 |
| P48679 | -0.0013 | 9.94E-01 | 0.2089 | 2.44E-01 |
| Q9WTP0 | -0.2467 | 3.67E-01 | -0.1575 | 5.41E-01 |
| P69897 | 0.0146 | 9.49E-01 | 0.2198 | 2.48E-01 |
| Q68FY1 | -0.2032 | 3.12E-01 | 0.0720 | 7.11E-01 |
| A0JPP1 | -0.1846 | 3.90E-01 | -0.1357 | 4.99E-01 |
| A0A096MKC0 | -0.0866 | 8.49E-01 | 0.4805 | 2.71E-01 |
| Q4V7E8 | -0.0805 | 4.42E-01 | -0.0811 | 4.31E-01 |
| F1MA59 | 0.0238 | 8.80E-01 | 0.1531 | 2.53E-01 |
| D3ZN95 | 0.0684 | 5.60E-01 | 0.1023 | 3.48E-01 |
| F1LT49 | -0.1274 | 5.07E-01 | -0.1659 | 3.82E-01 |
| D3ZAF6 | -0.0631 | 6.51E-01 | -0.1357 | 3.12E-01 |
| P05765 | -0.0939 | 3.61E-01 | -0.0580 | 5.64E-01 |
| Q6MGB8 | -0.1559 | 3.77E-01 | 0.1096 | 5.32E-01 |
| Q9QYU4 | 0.1232 | 5.77E-01 | -0.1966 | 3.45E-01 |
| Q6YH22 | -0.0100 | 9.38E-01 | -0.1202 | 2.55E-01 |
| B5DF55 | -0.0397 | 8.41E-01 | 0.1872 | 2.64E-01 |
| D4A259 | -0.1804 | 5.63E-01 | 0.2674 | 3.56E-01 |
| Q4FZT0 | 0.3387 | 3.06E-01 | 0.0837 | 7.96E-01 |
| B4F7B2 | -0.1575 | 3.47E-01 | -0.0775 | 6.31E-01 |
| D4A7X5 | 0.0236 | 9.45E-01 | -0.3196 | 2.58E-01 |
| D3ZFT1 | 0.1609 | 6.96E-01 | -0.3802 | 3.03E-01 |
| P10719 | 0.0130 | 9.11E-01 | 0.1122 | 2.58E-01 |
| Q498R7 | 0.0546 | 6.93E-01 | 0.1313 | 3.02E-01 |
| A0A0G2JV04 | -0.0268 | 8.69E-01 | 0.1538 | 2.63E-01 |
| A0A0G2JXI1 | -0.3537 | 4.34E-01 | 0.3222 | 4.62E-01 |
| P04797 | -0.0858 | 5.50E-01 | 0.1322 | 3.69E-01 |
| Q63228 | -0.0868 | 6.65E-01 | -0.1977 | 3.17E-01 |
| Q6AYK3 | 0.1675 | 3.60E-01 | 0.0953 | 5.97E-01 |
| P37397 | -0.0779 | 6.10E-01 | 0.1250 | 3.35E-01 |
| D3ZUV9 | 0.0835 | 6.03E-01 | 0.1385 | 3.41E-01 |
| E9PST5 | -0.0587 | 3.07E-01 | 0.0165 | 7.82E-01 |
| G3V7Z8 | -0.0690 | 4.77E-01 | -0.0727 | 4.27E-01 |
| P21531 | 0.3638 | 3.00E-01 | -0.0373 | 9.14E-01 |
| D4AEK9 | 0.1634 | 4.69E-01 | -0.1614 | 4.43E-01 |
| A0A0G2K9J2 | 0.0472 | 8.57E-01 | -0.2433 | 2.84E-01 |
| Q9WU82 | -0.1674 | 3.27E-01 | -0.0568 | 7.43E-01 |
| Q496Z1 | -0.1852 | 3.48E-01 | 0.0827 | 6.75E-01 |
| P53534 | -0.2525 | 3.04E-01 | 0.0481 | 8.54E-01 |
| Q9R1D1 | -0.1473 | 4.37E-01 | 0.1256 | 4.83E-01 |
| M0R515 | 0.2106 | 3.53E-01 | -0.1052 | 6.49E-01 |
| Q920P6 | -0.0994 | 5.97E-01 | 0.1620 | 3.58E-01 |
| Q6AYS7 | 0.1539 | 5.29E-01 | -0.1898 | 3.99E-01 |
| Q5U2Z3 | -0.3313 | 3.65E-01 | -0.1865 | 6.18E-01 |
| D3ZI68 | 0.1939 | 4.17E-01 | 0.1588 | 5.16E-01 |
| O08730 | -0.1301 | 5.19E-01 | -0.1592 | 4.09E-01 |
| F1LT30 | -0.1681 | 3.02E-01 | -0.0124 | 9.41E-01 |
| G3V6K6 | 0.1112 | 5.53E-01 | 0.1519 | 3.89E-01 |
| P02651 | -0.0445 | 9.39E-01 | 0.5082 | 2.75E-01 |
| B5DFC9 | -0.1110 | 3.80E-01 | 0.0669 | 5.97E-01 |
| D3Z9E1 | -0.0373 | 5.53E-01 | -0.0509 | 3.90E-01 |
| A0A0G2JWH3 | 0.1420 | 4.56E-01 | -0.1326 | 4.78E-01 |
| Q5EIC4 | -0.0167 | 9.61E-01 | 0.3174 | 2.84E-01 |
| Q62667 | 0.1253 | 5.78E-01 | 0.1803 | 3.78E-01 |
| D4ABK7 | -0.3247 | 3.45E-01 | -0.1121 | 7.38E-01 |
| Q6P7Q4 | 0.0422 | 8.85E-01 | -0.2792 | 2.86E-01 |
| Q5I0M3 | 0.1845 | 4.61E-01 | 0.1771 | 4.77E-01 |
| Q04753 | -0.0448 | 7.33E-01 | 0.1211 | 3.18E-01 |
| Q5U312 | 0.1293 | 3.61E-01 | 0.0606 | 6.64E-01 |
| P07171 | -0.3472 | 3.79E-01 | -0.1923 | 6.29E-01 |
| B2RYS3 | 0.1866 | 4.56E-01 | 0.1600 | 4.92E-01 |
| D4ACK1 | 0.2018 | 3.77E-01 | 0.1022 | 6.40E-01 |
| Q4VBH2 | -0.2770 | 3.39E-01 | 0.0723 | 8.06E-01 |
| P07936 | -0.1470 | 6.17E-01 | 0.2434 | 3.69E-01 |
| Q9JIL9 | -0.1805 | 6.26E-01 | 0.2975 | 3.66E-01 |
| Q5EAJ6 | -0.0939 | 6.28E-01 | 0.1586 | 3.69E-01 |
| D3ZDN6 | 0.1100 | 6.16E-01 | 0.1784 | 3.72E-01 |
| D4A987 | 0.2038 | 4.70E-01 | 0.1884 | 4.83E-01 |
| Q5U318 | -0.0881 | 3.15E-01 | 0.0112 | 9.10E-01 |
| P12711 | -0.0778 | 7.55E-01 | 0.2532 | 3.32E-01 |
| P23785 | 0.0776 | 6.25E-01 | 0.1313 | 3.71E-01 |
| M0RC54 | 0.0110 | 9.47E-01 | 0.1399 | 2.93E-01 |
| Q6P6S4 | -0.0630 | 8.94E-01 | 0.4210 | 3.09E-01 |
| D4A9W1 | -0.0883 | 7.59E-01 | 0.2462 | 3.26E-01 |
| F1M9I4 | 0.1079 | 5.21E-01 | 0.1254 | 4.51E-01 |
| Q03626 | 0.0180 | 9.47E-01 | 0.2321 | 2.99E-01 |
| P35565 | -0.1410 | 3.26E-01 | -0.0167 | 9.14E-01 |
| B5DEQ4 | 0.0844 | 8.42E-01 | 0.3843 | 3.20E-01 |
| Q5XHY0 | -0.1830 | 3.93E-01 | 0.1203 | 6.29E-01 |
| D3ZUU6 | 0.1491 | 6.20E-01 | -0.2353 | 3.89E-01 |
| E9PT53 | 0.1669 | 3.80E-01 | 0.0751 | 7.06E-01 |
| Q6GMN2 | -0.0402 | 8.62E-01 | 0.2093 | 3.18E-01 |
| Q9Z1K9 | -0.1696 | 4.47E-01 | 0.1344 | 5.46E-01 |
| Q9WUL0 | -0.1917 | 3.59E-01 | -0.0596 | 7.77E-01 |
| E9PTJ1 | -0.2841 | 3.86E-01 | -0.1203 | 7.02E-01 |
| D3ZH40 | 0.0157 | 9.59E-01 | -0.2643 | 3.13E-01 |
| F1LZJ4 | 0.0546 | 7.84E-01 | -0.1684 | 3.33E-01 |
| B1WC33 | 0.0555 | 6.54E-01 | -0.0995 | 3.81E-01 |
| D4A3P1 | 0.1192 | 3.47E-01 | 0.0241 | 8.56E-01 |
| Q99MA0 | 0.5319 | 3.54E-01 | -0.0603 | 9.14E-01 |
| B0BNB4 | -0.1482 | 3.47E-01 | 0.0238 | 8.88E-01 |
| Q4V886 | 0.0242 | 9.14E-01 | -0.2164 | 3.32E-01 |
| F1MAH5 | -0.0390 | 7.13E-01 | 0.0875 | 3.58E-01 |
| E9PT85 | -0.0743 | 7.18E-01 | 0.1769 | 3.62E-01 |
| D4AD33 | 0.1567 | 3.71E-01 | 0.0558 | 7.52E-01 |
| Q2KN99 | -0.1563 | 5.10E-01 | -0.1568 | 4.88E-01 |
| Q7TPB1 | 0.0842 | 8.31E-01 | 0.3336 | 3.35E-01 |
| Q6MGC4 | -0.0814 | 4.13E-01 | -0.0469 | 6.37E-01 |
| A0A0G2JVP9 | -0.1378 | 3.95E-01 | 0.0599 | 6.94E-01 |
| P13437 | 0.1657 | 3.56E-01 | -0.0292 | 8.73E-01 |
| Q561R9 | -0.0961 | 6.54E-01 | -0.1597 | 3.91E-01 |
| O35370 | -0.1121 | 7.01E-01 | -0.2362 | 3.77E-01 |
| P23680 | 0.1452 | 7.28E-01 | -0.3600 | 3.71E-01 |
| Q5PPI3 | 0.1529 | 6.08E-01 | -0.2207 | 4.30E-01 |
| A0A0G2JXZ5 | -0.0697 | 6.36E-01 | 0.1127 | 4.09E-01 |
| D3ZJ92 | 0.0394 | 7.48E-01 | 0.0989 | 3.63E-01 |
| M0RB65 | 0.2974 | 5.19E-01 | -0.2888 | 4.99E-01 |
| P69736 | 0.0336 | 7.76E-01 | 0.0951 | 3.55E-01 |
| P10817 | 0.1148 | 5.17E-01 | 0.1152 | 5.02E-01 |
| Q5BJT0 | 0.1389 | 4.29E-01 | 0.0830 | 6.31E-01 |
| M0R919 | -0.3514 | 3.69E-01 | -0.0341 | 9.28E-01 |
| G3V656 | -0.2590 | 3.73E-01 | 0.0907 | 7.85E-01 |
| Q99MZ8 | 0.0635 | 5.72E-01 | 0.0763 | 4.71E-01 |
| Q6AYC4 | 0.1067 | 7.33E-01 | 0.2405 | 3.80E-01 |
| P06214 | 0.0436 | 8.07E-01 | -0.1381 | 3.56E-01 |
| O88989 | 0.1163 | 4.71E-01 | 0.0876 | 5.85E-01 |
| A0A0G2K0W5 | 0.1075 | 6.09E-01 | 0.1507 | 4.55E-01 |
| P16290 | 0.2270 | 4.13E-01 | 0.1004 | 7.23E-01 |
| A0A0G2K7N7 | -0.0518 | 6.93E-01 | -0.0988 | 4.04E-01 |
| Q5BJK8 | 0.1420 | 4.32E-01 | 0.0758 | 6.74E-01 |
| D3ZM20 | -0.0345 | 8.39E-01 | -0.1323 | 3.59E-01 |
| A0A0G2K261 | 0.3518 | 3.77E-01 | 0.0017 | 9.97E-01 |
| Q5U4E6 | -0.0306 | 8.06E-01 | -0.0980 | 3.69E-01 |
| P55260 | 0.1004 | 7.69E-01 | -0.2587 | 3.86E-01 |
| F1LP90 | -0.0188 | 9.47E-01 | 0.2102 | 3.59E-01 |
| B0K015 | 0.2088 | 5.94E-01 | 0.2541 | 4.79E-01 |
| G3V829 | -0.0965 | 5.85E-01 | -0.1195 | 4.83E-01 |
| Q3KRD5 | -0.0900 | 5.88E-01 | -0.1121 | 4.79E-01 |
| P28042 | -0.0357 | 8.80E-01 | -0.1873 | 3.59E-01 |
| P49791 | 0.0238 | 8.53E-01 | 0.1035 | 3.68E-01 |
| O70593 | 0.1103 | 4.40E-01 | 0.0603 | 6.82E-01 |
| Q78ZR5 | -0.1218 | 3.94E-01 | -0.0296 | 8.44E-01 |
| P20767 | -0.1708 | 5.78E-01 | 0.1945 | 4.91E-01 |
| Q63910 | -0.1091 | 5.12E-01 | -0.1021 | 5.71E-01 |
| Q6AYA8 | -0.0725 | 8.25E-01 | 0.5089 | 4.05E-01 |
| D3ZXS8 | 0.0067 | 9.65E-01 | -0.1204 | 3.59E-01 |
| Q3MHS9 | -0.0761 | 8.79E-01 | 0.3896 | 3.69E-01 |
| B2GV74 | -0.0166 | 8.89E-01 | -0.0976 | 3.69E-01 |
| Q0ZFS8 | 0.0979 | 5.02E-01 | -0.0748 | 5.96E-01 |
| B5DFN4 | -0.1122 | 3.94E-01 | -0.0174 | 9.01E-01 |
| D3ZIY3 | -0.1026 | 4.18E-01 | -0.0358 | 7.80E-01 |
| Q5FVC5 | 0.0938 | 6.21E-01 | 0.1272 | 4.82E-01 |
| Q6TMG5 | -0.0164 | 8.58E-01 | -0.0684 | 3.73E-01 |
| D3ZYK9 | -0.1915 | 4.57E-01 | -0.1083 | 6.81E-01 |
| Q6IN36 | -0.0436 | 8.01E-01 | -0.1273 | 3.90E-01 |
| O70257 | -0.0660 | 5.88E-01 | -0.0761 | 5.04E-01 |
| O08815 | 0.0370 | 8.50E-01 | 0.1489 | 3.81E-01 |
| Q6AYD5 | -0.2723 | 4.09E-01 | -0.0358 | 9.13E-01 |
| Q5XI77 | -0.0245 | 9.19E-01 | 0.1897 | 3.81E-01 |
| P02454 | -0.0509 | 6.93E-01 | 0.0865 | 4.43E-01 |
| Q5RKI6 | 0.1222 | 7.31E-01 | 0.2691 | 4.38E-01 |
| D3ZGY1 | -0.0203 | 8.13E-01 | -0.0669 | 3.99E-01 |
| G3V9R8 | -0.1383 | 6.03E-01 | 0.1661 | 5.01E-01 |
| Q66H09 | -0.0935 | 5.22E-01 | -0.0804 | 5.96E-01 |
| P13264 | -0.1319 | 4.74E-01 | -0.0679 | 6.99E-01 |
| Q62638 | 0.0140 | 9.56E-01 | 0.1845 | 3.85E-01 |
| A0A0G2K0C5 | 0.1479 | 4.92E-01 | -0.0905 | 6.60E-01 |
| D4AE49 | 0.4100 | 4.12E-01 | -0.0351 | 9.47E-01 |
| B2GUV5 | 0.1161 | 4.13E-01 | -0.0160 | 9.13E-01 |
| M0RBE8 | 0.1458 | 4.65E-01 | -0.0662 | 7.28E-01 |
| P19804 | -0.0034 | 9.90E-01 | -0.2173 | 3.82E-01 |
| P10111 | -0.0723 | 4.77E-01 | -0.0427 | 6.81E-01 |
| P97536 | 0.0763 | 6.58E-01 | -0.1470 | 4.86E-01 |
| Q4KM35 | -0.2666 | 4.22E-01 | 0.0519 | 8.78E-01 |
| F1LMV6 | -0.1451 | 4.77E-01 | -0.0880 | 6.81E-01 |
| D3ZCL3 | -0.0956 | 5.25E-01 | -0.0703 | 6.08E-01 |
| A0A0G2JZV7 | -0.1995 | 4.29E-01 | -0.0436 | 8.58E-01 |
| Q8CGS5 | -0.0407 | 8.39E-01 | -0.1428 | 4.00E-01 |
| D3ZZ51 | 0.0742 | 6.01E-01 | 0.0840 | 5.21E-01 |
| D4A7U6 | -0.1602 | 4.22E-01 | 0.0281 | 8.93E-01 |
| P83871 | -0.1494 | 4.18E-01 | 0.0179 | 9.24E-01 |
| Q66H76 | -0.0432 | 5.78E-01 | -0.0436 | 5.50E-01 |
| Q6AYK8 | -0.0396 | 8.41E-01 | -0.1430 | 4.05E-01 |
| P62628 | -0.0702 | 6.74E-01 | -0.1063 | 4.76E-01 |
| P51583 | -0.0957 | 5.98E-01 | -0.1041 | 5.32E-01 |
| P01836 | -0.2994 | 5.28E-01 | 0.2399 | 6.27E-01 |
| D4A3M7 | -0.0586 | 7.07E-01 | -0.1045 | 4.59E-01 |
| P62832 | -0.1126 | 4.43E-01 | -0.0351 | 8.15E-01 |
| Q63692 | 0.1699 | 6.59E-01 | 0.2392 | 4.93E-01 |
| P40190 | -0.1562 | 6.20E-01 | 0.1888 | 5.24E-01 |
| F1M8A5 | 0.0440 | 6.55E-01 | -0.0620 | 4.95E-01 |
| P02767 | 0.1867 | 4.29E-01 | -0.0277 | 9.11E-01 |
| F1LQH9 | -0.1181 | 4.29E-01 | 0.0071 | 9.63E-01 |
| G3V8E4 | 0.1979 | 5.92E-01 | 0.2050 | 5.53E-01 |
| Q5U2M7 | 0.1452 | 4.80E-01 | -0.0702 | 7.41E-01 |
| Q9Z1W6 | -0.0353 | 8.73E-01 | 0.1523 | 4.12E-01 |
| Q9Z244 | 0.2557 | 6.07E-01 | -0.2775 | 5.47E-01 |
| Q8K3X0 | 0.0445 | 6.93E-01 | 0.0719 | 4.83E-01 |
| Q5XI17 | -0.0309 | 7.39E-01 | 0.0665 | 4.68E-01 |
| Q4KLZ3 | -0.1805 | 4.90E-01 | -0.0894 | 7.28E-01 |
| P62804 | -0.0676 | 8.79E-01 | -0.3088 | 4.17E-01 |
| P43244 | -0.0678 | 6.23E-01 | -0.0809 | 5.41E-01 |
| Q4KM75 | 0.1421 | 5.86E-01 | 0.1377 | 5.82E-01 |
| A0A0G2K548 | -0.0896 | 7.55E-01 | -0.1855 | 4.62E-01 |
| Q7TT49 | -0.0969 | 5.66E-01 | -0.0819 | 6.09E-01 |
| B2RYF7 | 0.0127 | 9.70E-01 | 0.2401 | 4.13E-01 |
| B5DES0 | -0.1087 | 4.56E-01 | 0.0275 | 8.60E-01 |
| G3V7Q4 | 0.1130 | 6.32E-01 | 0.1341 | 5.51E-01 |
| A0A0G2KAP8 | -0.1269 | 5.65E-01 | -0.1152 | 6.29E-01 |
| D4AA11 | -0.1328 | 4.18E-01 | -0.0202 | 9.52E-01 |
| A0A0G2K5D7 | 0.1340 | 4.56E-01 | -0.0259 | 8.92E-01 |
| G3V624 | 0.0903 | 8.09E-01 | 0.2588 | 4.54E-01 |
| Q99PS2 | -0.1379 | 4.88E-01 | 0.0576 | 7.75E-01 |
| Q63347 | -0.0258 | 9.06E-01 | 0.1574 | 4.31E-01 |
| O88600 | 0.0247 | 8.35E-01 | 0.0805 | 4.48E-01 |
| Q5U2V4 | 0.1415 | 4.70E-01 | 0.0257 | 8.88E-01 |
| P50463 | 0.1074 | 4.52E-01 | 0.0062 | 9.69E-01 |
| A0A0U1RRW3 | -0.0157 | 9.61E-01 | -0.2097 | 4.33E-01 |
| Q6URK4 | -0.0896 | 4.70E-01 | -0.0243 | 8.52E-01 |
| P28023 | 0.0433 | 7.79E-01 | 0.1024 | 4.72E-01 |
| Q63364 | -0.0353 | 7.36E-01 | -0.0643 | 4.91E-01 |
| Q8K5B3 | -0.0279 | 8.52E-01 | -0.0989 | 4.50E-01 |
| P15473 | -0.0051 | 9.70E-01 | 0.0958 | 4.32E-01 |
| D3ZZ38 | -0.0941 | 4.99E-01 | -0.0361 | 7.92E-01 |
| Q6AXW2 | -0.0593 | 7.04E-01 | 0.0973 | 5.16E-01 |
| Q32PX2 | 0.1409 | 5.50E-01 | -0.0863 | 6.94E-01 |
| F7F350 | -0.0851 | 5.22E-01 | -0.0491 | 7.27E-01 |
| Q63797 | -0.0401 | 7.66E-01 | -0.0818 | 4.86E-01 |
| F1M6B3 | 0.0085 | 9.60E-01 | -0.1071 | 4.44E-01 |
| D3ZTL0 | -0.1440 | 4.99E-01 | -0.0324 | 8.73E-01 |
| Q5BJP3 | -0.0915 | 5.49E-01 | 0.0591 | 7.01E-01 |
| P18437 | 0.0041 | 9.92E-01 | 0.2549 | 4.48E-01 |
| D3ZWA1 | -0.2284 | 4.90E-01 | 0.0435 | 9.00E-01 |
| P35213 | 0.0916 | 6.74E-01 | 0.1169 | 5.70E-01 |
| D4A7H9 | 0.0437 | 8.24E-01 | 0.1188 | 4.85E-01 |
| D3ZXH7 | 0.0458 | 6.25E-01 | 0.0443 | 6.14E-01 |
| B0K019 | 0.1423 | 5.16E-01 | 0.0579 | 8.08E-01 |
| Q99PD6 | -0.0147 | 9.49E-01 | 0.1426 | 4.63E-01 |
| Q5XI72 | 0.0047 | 9.69E-01 | 0.0769 | 4.62E-01 |
| Q6AY71 | -0.0859 | 4.94E-01 | -0.0084 | 9.49E-01 |
| A4L9P7 | 0.3204 | 6.78E-01 | 0.3910 | 5.82E-01 |
| D4A6G6 | 0.2253 | 6.39E-01 | 0.2209 | 6.16E-01 |
| D4A427 | -0.0134 | 9.23E-01 | -0.0871 | 4.76E-01 |
| D3ZJR1 | -0.0122 | 8.35E-01 | 0.0363 | 4.95E-01 |
| D4A9P7 | -0.0518 | 7.37E-01 | 0.0834 | 5.41E-01 |
| P54290 | -0.0821 | 5.10E-01 | 0.0106 | 9.31E-01 |
| Q80WL2 | -0.0697 | 7.54E-01 | 0.1311 | 5.51E-01 |
| Q9WVC0 | 0.0102 | 9.45E-01 | 0.0875 | 4.81E-01 |
| B2RZ74 | -0.0599 | 6.33E-01 | -0.0558 | 6.45E-01 |
| D3ZBT2 | -0.0444 | 8.15E-01 | -0.1085 | 5.16E-01 |
| E9PTG8 | 0.0524 | 8.32E-01 | 0.1431 | 5.12E-01 |
| P63018 | -0.0711 | 5.55E-01 | 0.0309 | 8.00E-01 |
| P04638 | 0.2203 | 5.88E-01 | -0.1368 | 7.31E-01 |
| Q9ESN0 | 0.0321 | 8.83E-01 | -0.1299 | 5.04E-01 |
| F7FD47 | -0.2272 | 6.28E-01 | -0.1759 | 6.87E-01 |
| M0R6E6 | -0.0877 | 5.36E-01 | -0.0149 | 9.14E-01 |
| D4A6C5 | 0.1195 | 6.70E-01 | -0.1194 | 6.39E-01 |
| O35987 | -0.0535 | 5.79E-01 | 0.0257 | 7.79E-01 |
| Q0ZFS5 | -0.0839 | 6.84E-01 | 0.0970 | 6.34E-01 |
| Q6P6V0 | 0.0824 | 7.07E-01 | -0.1023 | 6.16E-01 |
| B0BMY5 | -0.0332 | 9.04E-01 | -0.1550 | 5.12E-01 |
| D4A4T9 | -0.1478 | 5.95E-01 | -0.0877 | 7.51E-01 |
| F1M062 | -0.0038 | 9.84E-01 | -0.1073 | 5.14E-01 |
| G3V8R0 | -0.0407 | 7.56E-01 | -0.0640 | 5.83E-01 |
| F1MAN8 | 0.0630 | 7.28E-01 | -0.0872 | 6.06E-01 |
| Q6AY58 | 0.0172 | 9.02E-01 | -0.0776 | 5.21E-01 |
| A0A0G2JY58 | -0.0603 | 7.44E-01 | 0.1101 | 6.10E-01 |
| P07872 | -0.1002 | 8.41E-01 | -0.2699 | 5.47E-01 |
| B2RZB7 | 0.1327 | 5.84E-01 | 0.0540 | 8.18E-01 |
| F1LXD8 | -0.0075 | 9.73E-01 | 0.1294 | 5.17E-01 |
| Q99MM4 | 0.0392 | 7.55E-01 | 0.0595 | 5.97E-01 |
| Q3KRF1 | 0.0278 | 9.25E-01 | 0.1633 | 5.32E-01 |
| P47875 | 0.0485 | 6.98E-01 | 0.0531 | 6.46E-01 |
| D3ZJT4 | 0.1206 | 5.69E-01 | 0.0268 | 9.00E-01 |
| Q6AYH5 | 0.0069 | 9.66E-01 | 0.0908 | 5.25E-01 |
| P11608 | -0.1556 | 5.98E-01 | 0.0713 | 8.04E-01 |
| P16303 | 0.0568 | 7.11E-01 | 0.0656 | 6.38E-01 |
| G3V852 | 0.0091 | 9.51E-01 | 0.0795 | 5.31E-01 |
| Q4FZU4 | 0.0602 | 8.35E-01 | 0.1505 | 5.64E-01 |
| Q6U6G5 | 0.0364 | 7.47E-01 | 0.0503 | 6.14E-01 |
| F1LQ00 | 0.0013 | 9.92E-01 | -0.0610 | 5.25E-01 |
| Q66H24 | 0.1179 | 7.12E-01 | -0.1343 | 6.52E-01 |
| M0R8B5 | 0.1332 | 5.87E-01 | 0.0479 | 8.62E-01 |
| F2Z3T9 | -0.0544 | 7.34E-01 | 0.0734 | 6.29E-01 |
| Q3T1J1 | -0.0668 | 6.32E-01 | 0.0438 | 7.50E-01 |
| P70483 | -0.1585 | 5.83E-01 | 0.0239 | 9.32E-01 |
| P49242 | -0.0992 | 6.16E-01 | -0.0464 | 8.04E-01 |
| Q4QRB2 | 0.0060 | 9.67E-01 | 0.0785 | 5.45E-01 |
| Q4QQW8 | -0.1802 | 6.74E-01 | -0.1422 | 7.18E-01 |
| A0A0G2JSK1 | 0.3613 | 7.04E-01 | 0.3717 | 6.76E-01 |
| A0A0G2JUG7 | -0.1458 | 5.86E-01 | 0.0060 | 9.83E-01 |
| P97697 | 0.0779 | 7.25E-01 | -0.0909 | 6.60E-01 |
| Q6JHU9 | -0.0503 | 8.24E-01 | -0.1008 | 6.03E-01 |
| Q62848 | -0.0587 | 7.21E-01 | 0.0669 | 6.66E-01 |
| P29266 | -0.1998 | 6.01E-01 | 0.0364 | 9.20E-01 |
| P38650 | -0.0688 | 8.23E-01 | -0.1457 | 6.03E-01 |
| P50878 | 0.0156 | 9.19E-01 | -0.0772 | 5.65E-01 |
| O08949 | 0.0114 | 9.65E-01 | -0.1278 | 5.66E-01 |
| Q9R037 | -0.0349 | 6.87E-01 | -0.0285 | 7.27E-01 |
| P70478 | 0.0817 | 6.99E-01 | 0.0734 | 7.20E-01 |
| F7EY92 | -0.1126 | 6.54E-01 | 0.0635 | 7.96E-01 |
| D4A720 | 0.1494 | 6.06E-01 | -0.0029 | 9.92E-01 |
| Q6AY02 | -0.0490 | 9.07E-01 | 0.1987 | 5.97E-01 |
| Q5RK17 | -0.0153 | 9.54E-01 | -0.1291 | 5.88E-01 |
| Q497B0 | 0.0977 | 6.65E-01 | 0.0575 | 7.89E-01 |
| A0A0G2JY08 | -0.0492 | 7.41E-01 | 0.0548 | 6.91E-01 |
| D3ZDZ1 | 0.0948 | 6.70E-01 | 0.0599 | 7.90E-01 |
| Q66H11 | -0.0193 | 9.54E-01 | 0.1505 | 5.92E-01 |
| P00786 | -0.1475 | 7.93E-01 | -0.2316 | 6.55E-01 |
| D3ZKQ4 | -0.0470 | 7.06E-01 | -0.0361 | 7.37E-01 |
| B2RYW9 | -0.0560 | 8.16E-01 | -0.1030 | 6.42E-01 |
| Q6P685 | 0.0335 | 8.55E-01 | 0.0797 | 6.21E-01 |
| G3V953 | -0.0851 | 6.36E-01 | 0.0147 | 9.23E-01 |
| P25113 | -0.0180 | 9.36E-01 | -0.0976 | 6.14E-01 |
| D3Z8E6 | 0.0964 | 6.46E-01 | -0.0249 | 9.07E-01 |
| D3ZVQ0 | -0.2705 | 6.51E-01 | -0.1217 | 8.72E-01 |
| A0A0G2K1B6 | 0.0501 | 7.97E-01 | -0.0716 | 6.83E-01 |
| Q63768 | 0.1199 | 6.65E-01 | -0.0512 | 8.54E-01 |
| D3ZUX7 | -0.0052 | 9.86E-01 | -0.1391 | 6.09E-01 |
| D3ZW57 | 0.0961 | 6.56E-01 | 0.0288 | 8.92E-01 |
| Q7TP40 | -0.0351 | 7.70E-01 | 0.0418 | 7.01E-01 |
| M0R4L7 | -0.0809 | 7.05E-01 | -0.0549 | 7.84E-01 |
| Q7TP05 | 0.0013 | 9.94E-01 | -0.1066 | 6.14E-01 |
| Q5U211 | 0.0073 | 9.66E-01 | -0.0785 | 6.19E-01 |
| A0A0G2JZZ4 | 0.1203 | 6.70E-01 | 0.0456 | 8.64E-01 |
| Q5U3Y8 | 0.0022 | 9.81E-01 | 0.0416 | 6.19E-01 |
| P85972 | -0.0260 | 7.97E-01 | -0.0366 | 6.88E-01 |
| Q6YDN8 | -0.0458 | 8.52E-01 | 0.0961 | 6.59E-01 |
| Q6MG49 | 0.0184 | 8.85E-01 | 0.0545 | 6.39E-01 |
| Q63632 | 0.0686 | 6.59E-01 | -0.0131 | 9.28E-01 |
| P32038 | -0.0545 | 7.01E-01 | 0.0346 | 8.00E-01 |
| O88828 | -0.0918 | 7.59E-01 | -0.0907 | 7.32E-01 |
| Q6P6U6 | -0.0362 | 9.02E-01 | -0.1200 | 6.37E-01 |
| P68511 | 0.0577 | 7.75E-01 | 0.0697 | 7.12E-01 |
| A0A0G2JSL0 | -0.0481 | 6.81E-01 | -0.0222 | 8.48E-01 |
| Q99068 | -0.0256 | 7.84E-01 | -0.0334 | 7.06E-01 |
| Q4KLG9 | 0.0782 | 6.55E-01 | 0.0044 | 9.81E-01 |
| O35986 | 0.0060 | 9.65E-01 | -0.0594 | 6.32E-01 |
| Q9Z1Z9 | -0.0266 | 8.34E-01 | 0.0459 | 6.81E-01 |
| P63255 | 0.0258 | 8.57E-01 | 0.0561 | 6.67E-01 |
| D4A7F2 | -0.1269 | 6.65E-01 | -0.0041 | 9.90E-01 |
| Q5M7V8 | 0.0162 | 9.02E-01 | 0.0533 | 6.48E-01 |
| Q09167 | -0.0319 | 6.65E-01 | 0.0048 | 9.50E-01 |
| D4A5I4 | -0.0146 | 9.69E-01 | 0.1508 | 6.40E-01 |
| D4AE79 | -0.0909 | 6.81E-01 | 0.0206 | 9.22E-01 |
| Q6RJR6 | -0.0660 | 7.79E-01 | 0.0762 | 7.43E-01 |
| Q5BK07 | 0.0385 | 6.74E-01 | -0.0009 | 9.93E-01 |
| D4A772 | -0.0979 | 7.12E-01 | 0.0513 | 8.48E-01 |
| P31232 | -0.0776 | 7.26E-01 | -0.0472 | 8.23E-01 |
| A0A0G2KAI2 | -0.0588 | 6.81E-01 | 0.0057 | 9.70E-01 |
| M0RA08 | 0.0680 | 7.09E-01 | 0.0307 | 8.62E-01 |
| Q157S1 | 0.0545 | 8.23E-01 | -0.0786 | 7.26E-01 |
| D4A4Q7 | 0.0047 | 9.54E-01 | 0.0312 | 6.61E-01 |
| Q5U2U7 | 0.1662 | 8.09E-01 | 0.2005 | 7.43E-01 |
| D3ZSY8 | 0.0437 | 8.18E-01 | 0.0582 | 7.41E-01 |
| A0JN30 | -0.0555 | 7.07E-01 | -0.0131 | 9.25E-01 |
| Q66H91 | 0.0834 | 7.72E-01 | 0.0666 | 8.00E-01 |
| O35274 | -0.0204 | 8.67E-01 | 0.0423 | 7.12E-01 |
| O08557 | -0.0361 | 7.96E-01 | 0.0418 | 7.77E-01 |
| D4A0D9 | -0.0587 | 7.90E-01 | 0.0567 | 7.81E-01 |
| Q5PQK4 | -0.0468 | 8.49E-01 | 0.0726 | 7.37E-01 |
| F1M6T6 | 0.0272 | 8.89E-01 | -0.0840 | 7.27E-01 |
| Q3ZB99 | -0.0267 | 9.45E-01 | -0.1248 | 7.01E-01 |
| Q63081 | 0.0352 | 8.22E-01 | -0.0472 | 7.68E-01 |
| P09527 | -0.0586 | 8.13E-01 | -0.0658 | 7.78E-01 |
| P19814 | -0.0525 | 8.41E-01 | -0.0739 | 7.52E-01 |
| O88321 | -0.0335 | 7.83E-01 | -0.0273 | 8.11E-01 |
| O55004 | 0.0330 | 9.06E-01 | -0.0928 | 7.18E-01 |
| Q6P4Z9 | -0.0630 | 7.58E-01 | -0.0327 | 8.69E-01 |
| P62994 | -0.0460 | 9.09E-01 | -0.1201 | 7.38E-01 |
| D3ZY35 | -0.0752 | 7.97E-01 | -0.0572 | 8.47E-01 |
| P56603 | 0.0213 | 9.30E-01 | -0.0676 | 7.37E-01 |
| P11517 | 0.0764 | 8.74E-01 | -0.1304 | 7.72E-01 |
| G3V992 | -0.1081 | 7.67E-01 | 0.0262 | 9.39E-01 |
| B1WC84 | 0.0211 | 8.49E-01 | 0.0273 | 7.96E-01 |
| D4A2P1 | 0.0234 | 9.02E-01 | 0.0538 | 7.63E-01 |
| P04166 | -0.0876 | 8.03E-01 | -0.0518 | 8.72E-01 |
| Q4G079 | -0.0030 | 9.76E-01 | -0.0323 | 7.46E-01 |
| F1M6F4 | -0.0640 | 7.72E-01 | 0.0094 | 9.64E-01 |
| B2GV37 | -0.0539 | 7.69E-01 | -0.0003 | 9.99E-01 |
| Q6PDU1 | 0.0532 | 7.84E-01 | 0.0215 | 9.11E-01 |
| D3ZN76 | -0.0012 | 9.93E-01 | -0.0386 | 7.47E-01 |
| Q9JM80 | -0.0172 | 9.42E-01 | -0.0617 | 7.58E-01 |
| A0A0G2K4U7 | 0.0323 | 9.31E-01 | 0.1008 | 7.66E-01 |
| A0A0G2JYC4 | -0.0054 | 9.83E-01 | 0.0678 | 7.55E-01 |
| Q9ET50 | -0.0089 | 9.73E-01 | -0.0813 | 7.63E-01 |
| M0R5F8 | -0.0438 | 7.78E-01 | 0.0015 | 9.92E-01 |
| Q499T2 | 0.0507 | 8.50E-01 | 0.0556 | 8.27E-01 |
| B2RYS9 | 0.0413 | 7.84E-01 | -0.0053 | 9.71E-01 |
| D3ZLX2 | 0.0522 | 8.18E-01 | -0.0246 | 9.01E-01 |
| Q3KR55 | 0.0122 | 9.49E-01 | 0.0479 | 7.81E-01 |
| B2RZ77 | 0.0435 | 8.92E-01 | -0.0718 | 8.12E-01 |
| B2RYU7 | 0.0420 | 8.62E-01 | 0.0461 | 8.35E-01 |
| D3ZHB3 | -0.0551 | 8.57E-01 | -0.0534 | 8.53E-01 |
| P06759 | -0.1224 | 8.21E-01 | -0.0559 | 9.15E-01 |
| F1LV37 | -0.0133 | 8.82E-01 | 0.0176 | 8.27E-01 |
| Q6IUP3 | -0.0822 | 8.05E-01 | 0.0055 | 9.87E-01 |
| Q62627 | -0.0368 | 8.55E-01 | 0.0293 | 8.64E-01 |
| D4A9M4 | 0.0164 | 9.54E-01 | 0.0587 | 8.12E-01 |
| A0JPJ7 | -0.0410 | 8.58E-01 | 0.0334 | 8.91E-01 |
| D4A779 | -0.0547 | 8.47E-01 | 0.0282 | 9.16E-01 |
| Q6MG66 | 0.0279 | 9.23E-01 | -0.0526 | 8.37E-01 |
| M0R6B9 | -0.0025 | 9.86E-01 | -0.0349 | 8.26E-01 |
| F1LML7 | -0.0048 | 9.84E-01 | -0.0356 | 8.25E-01 |
| Q642A5 | 0.0240 | 9.02E-01 | 0.0264 | 8.71E-01 |
| Q99M64 | -0.0258 | 9.31E-01 | -0.0489 | 8.53E-01 |
| D4ABD7 | -0.0207 | 9.19E-01 | 0.0330 | 8.58E-01 |
| Q9QVC8 | 0.0207 | 8.93E-01 | -0.0205 | 8.91E-01 |
| D3ZXI2 | 0.0342 | 8.82E-01 | -0.0253 | 9.07E-01 |
| B2RYP6 | -0.0206 | 8.73E-01 | -0.0105 | 9.33E-01 |
| A0A0G2K8Q8 | 0.0422 | 8.81E-01 | -0.0251 | 9.24E-01 |
| D3ZDK7 | -0.0208 | 9.06E-01 | -0.0216 | 9.00E-01 |
| B2RYB8 | -0.0126 | 9.51E-01 | 0.0280 | 8.71E-01 |
| B2RZ08 | -0.0227 | 9.02E-01 | 0.0165 | 9.21E-01 |
| Q2THW7 | -0.0097 | 9.47E-01 | -0.0129 | 9.14E-01 |
| P35435 | -0.0092 | 9.70E-01 | -0.0275 | 9.06E-01 |
| P83868 | 0.0124 | 9.61E-01 | 0.0260 | 9.12E-01 |
| A0A0G2JW94 | -0.0113 | 9.30E-01 | -0.0082 | 9.44E-01 |
| Q7TQ94 | -0.0128 | 9.70E-01 | 0.0324 | 9.20E-01 |
| F1LXP8 | 0.0015 | 9.93E-01 | -0.0171 | 9.15E-01 |
| D3ZZR9 | -0.0152 | 9.47E-01 | -0.0068 | 9.74E-01 |
| D3ZAY8 | -0.0094 | 9.73E-01 | 0.0103 | 9.69E-01 |
| Q99JE6 | -0.0023 | 9.93E-01 | 0.0124 | 9.63E-01 |
| B2GV58 | -0.0031 | 9.73E-01 | -0.0010 | 9.92E-01 |
| P24050 | 0.0018 | 9.93E-01 | 0.0056 | 9.78E-01 |
| Q4KMA2 | 0.0020 | 9.79E-01 | 0.0006 | 9.93E-01 |
| O35814 | 0.0002 | 9.97E-01 | 0.0014 | 9.86E-01 |
